# Shared Gene Structures and Clusters of Mutually Exclusive Spliced Exons within the Metazoan Muscle Myosin Heavy Chain Genes

**DOI:** 10.1371/journal.pone.0088111

**Published:** 2014-02-03

**Authors:** Martin Kollmar, Klas Hatje

**Affiliations:** Group Systems Biology of Motor Proteins, Department of NMR-based Structural Biology, Max-Planck-Institute for Biophysical Chemistry, Göttingen, Germany; University of Vienna, Max F. Perutz Laboratories, Austria

## Abstract

Multicellular animals possess two to three different types of muscle tissues. Striated muscles have considerable ultrastructural similarity and contain a core set of proteins including the muscle myosin heavy chain (*Mhc*) protein. The ATPase activity of this myosin motor protein largely dictates muscle performance at the molecular level. Two different solutions to adjusting myosin properties to different muscle subtypes have been identified so far: Vertebrates and nematodes contain many independent differentially expressed *Mhc* genes while arthropods have single *Mhc* genes with clusters of mutually exclusive spliced exons (MXEs). The availability of hundreds of metazoan genomes now allowed us to study whether the ancient bilateria already contained MXEs, how MXE complexity subsequently evolved, and whether additional scenarios to control contractile properties in different muscles could be proposed, By reconstructing the *Mhc* genes from 116 metazoans we showed that all intron positions within the motor domain coding regions are conserved in all bilateria analysed. The last common ancestor of the bilateria already contained a cluster of MXEs coding for part of the loop-2 actin-binding sequence. Subsequently the protostomes and later the arthropods gained many further clusters while MXEs got completely lost independently in several branches (vertebrates and nematodes) and species (for example the annelid *Helobdella robusta* and the salmon louse *Lepeophtheirus salmonis*). Several bilateria have been found to encode multiple *Mhc* genes that might all or in part contain clusters of MXEs. Notable examples are a cluster of six tandemly arrayed *Mhc* genes, of which two contain MXEs, in the owl limpet *Lottia gigantea* and four *Mhc* genes with three encoding MXEs in the predatory mite *Metaseiulus occidentalis*. Our analysis showed that similar solutions to provide different myosin isoforms (multiple genes or clusters of MXEs or both) have independently been developed several times within bilaterian evolution.

## Introduction

Alternative splicing of mutually exclusive exons (MXEs) is an important mechanism to increase the protein diversity in eukaryotes [1]. MXEs are neighboring exons that are spliced in a mutually exclusive manner into the mature transcript. In addition to identical reading frames and splice site patterns, these exons in almost all cases have similar lengths and show sequence similarity [2]. In vertebrates, MXEs have only been found in pairs. In contrast, larger clusters have been found in many insect genes [2]–[4] with even more than 50 MXEs per cluster in *Drosophila Dscam* genes [5]. In addition, genes can contain several clusters of MXEs giving rise to remarkable numbers of potential transcripts [4], [5]. As implied by the characteristics of MXEs, the resulting protein structures are identical except for the small regions, in which the different MXEs are incorporated to fine-tune protein function.

The *Drosophila melanogaster* muscle myosin heavy chain (*Mhc*) gene is a well-analysed example for a gene with multiple clusters of MXEs [6]–[9]. Four of its five clusters of MXEs encode parts of the myosin motor domain. Through specific combinations of MXEs the mechanochemical properties of the *Mhc's* are changed and adjusted to the needs of the different muscle types in a spatiotemporal manner. This is in contrast to other organisms of the metazoan lineage, which have a family of muscle myosin heavy chain genes with each gene coding for a protein specialized for a functional niche [10]–[12].

The muscle myosin heavy chain genes of 22 arthropod species ranging from waterflea to wasp and *Drosophila* have been annotated [4]. The analysis of the gene structures allowed the reconstruction of an ancient arthropod muscle myosin heavy chain gene and showed that during evolution of the arthropods introns have mainly been lost in these genes although intron gain might have happened in a few cases. Compared to the well-studied gene of *Drosophila melanogaster* other arthropod genes might contain up to four additional alternatively spliced exons encoding part of the motor domain. This considerably extends the possibilities of other arthropod species to fine-tune myosin and thus muscle characteristics.

Based on recently finished genome assemblies of many arthropods and other metazoan species we have analysed the evolution of the *Mhc* gene across metazoans with a focus on those encoding clusters of MXEs. 116 species have been analysed, the respective *Mhc* genes identified and reconstructed and the mutually exclusive splicing pattern elucidated, if such splice variants existed. Examples of Lepidoptera, Diptera, and Hymenoptera *Mhc* genes have already been analysed and described in detail elsewhere [4] and we will therefore focus on recently sequenced species and new clusters of MXEs.

## Results and Discussion

### Assembly of sequences and tree generation

**Figure 1:**
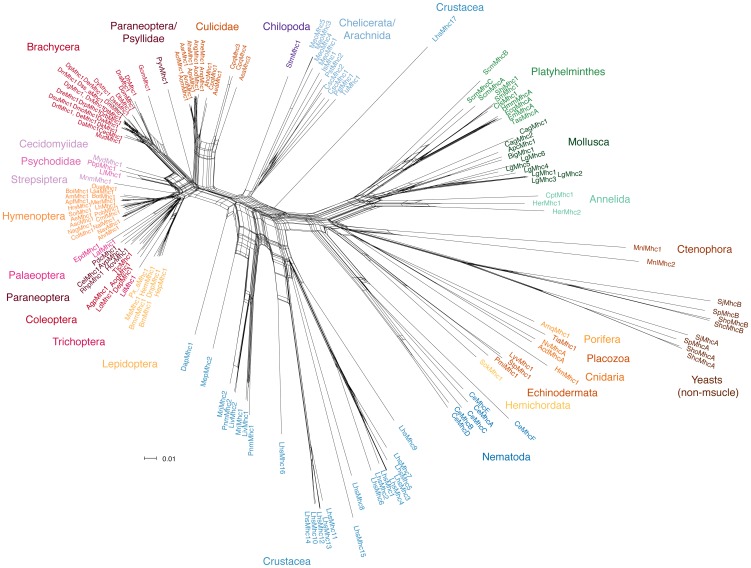
The unrooted phylogenetic split network was generated with SplitsTree using the NeighborNet method. The network presents alternative splits in the evolution of the muscle myosin heavy chain (*Mhc*) proteins. The *Schizosaccharomyces* non-muscle *Mhc* proteins have been used as outgroup. The phylogenetic trees based on the same data using three different methods are shown in Figure S1.

The muscle myosin heavy chain genes belong to the class-II myosins. At the sequence level, muscle myosin subtypes can only be distinguished from the non-muscle myosin isoforms if homologs from closely related species are available. To ensure that we did not miss duplicates or divergent homologs, we first identified and assembled all class-II myosins in the analysed metazoans and then verified muscle and non-muscle subtypes by phylogenetic grouping with known examples obtained from [13]. The muscle myosins were collected and a comparative phylogenetic analysis was performed using the Neighbour-Joining (NJ), Maximum-Likelihood (ML), Bayesian and split network approaches (Figure 1, Figure S1). As outgroup we choose the non-muscle myosins from four *Schizosaccharomyces* species. The topologies of the trees are similar and in accordance with recent species phylogenies, grouping for example nematode sequences closest to arthropod sequences and Platyhelminths within the Lophotrochozoa. These trees were therefore used as basis for the analysis of MXE cluster gain and loss events along the metazoan history.

**Figure 2:**
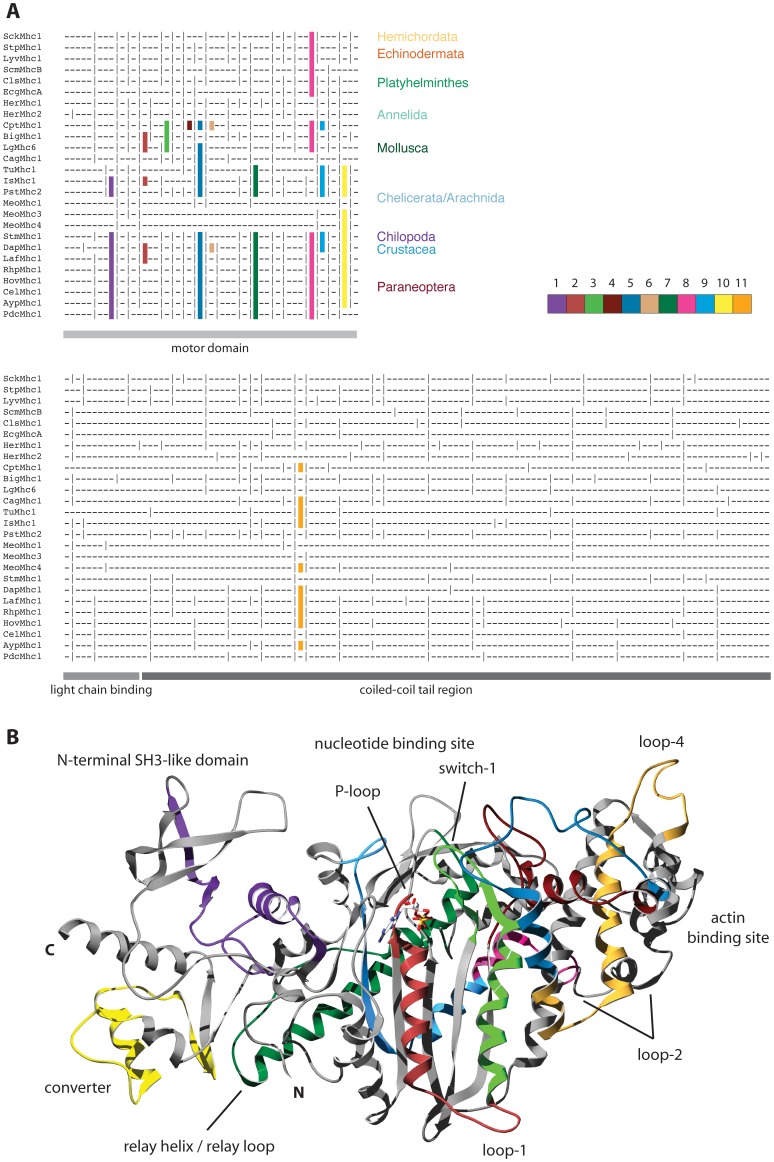
Conserved intron positions and location of MXE encoded regions. A) The gene structure alignment was generated with Genepainter [58] by mapping intron positions obtained from the gene structure reconstructions onto the protein multiple sequence alignment. Genepainter requires intron positions not only conserved at the amino-acid level but also at the nucleotide level (codons might be split differently). Hyphens “-” represent coding regions and vertical bars “|” denote intron positions. Common intron positions in the gene structure alignment are conserved down to the nucleotide level. Conserved clusters of MXEs are colour coded and numbered from N- to C-terminus (see legend). The same colour coding and numbering scheme will be used throughout this analysis for all MXEs. Some branch names are given for better orientation. B) The structure of the motor domain of the non-muscle class-II myosin of *Dictyostelium discoideum* [59] has been used to highlight the regions encoded by alternatively spliced exons. For colouring the regions encoded by MXEs the same colours have been used as for the gene structures in A). The clusters of MXEs not described so far code for the light-green (cluster-3) and the dark-brown (cluster-4) part of the structure.

Clusters of MXEs within the muscle myosin genes were predicted with WebScipio [5], which determines MXEs based on reading frame conservation, sequence similarity, and lengths constraints. Mutually exclusive inclusion of these exons in transcripts could be shown for many genes based on EST/cDNA data available at GenBank. EST/cDNA data was also used to confirm many of the differentially included C-terminal exons. The gene structures of the *Mhc* genes were compared at the base-pair level to reveal intron positions and clusters of MXEs conserved between branches (Figure 2A). It has already been pointed out in a previous comparison of the gene structures of 25 arthropod *Mhc* genes that in general introns had been lost during evolution and not gained [4]. Within the motor domain coding region, all intron positions were found to be conserved in at least two of the sequences, while there were still many unique intron positions in the coiled-coil tail region. The motor domain coding region of the proposed ancient arthropod *Mhc* gene was predicted to resemble the *Daphnia Mhc1* gene [4]. Proposed common exons of the coiled-coil tail coding region of the ancient *Mhc* gene would have been two to three times longer than common exons coding for the motor domain [4]. Our analysis here shows that all intron positions within the motor domain coding regions of the analysed *Mhc* genes are conserved across the bilateria and must have therefore been present in the ancient bilaterian *Mhc* gene (Figure 2A). The only exception is the intron following MXE cluster-6, which is shifted by 1 bp in arthropods. The other positions that do not seem to be shared in the scheme are located after loop-1 and within loop-2 where the protein sequence alignment is ambiguous. The gene structure alignment also shows that most of the intron positions within the coiled-coil tail region are conserved between at least two of the sequences shown (almost all positions are conserved across all 116 species of this analysis; data not shown) implicating that these were all present in the ancient bilaterian muscle *Mhc* gene (Figure 2A). This strongly supports our previous notion that the ancient *Mhc* gene was intron-rich and that most of its introns got lost during subsequent evolution.

### Location of the MXEs within the myosin motor domain

The locations and potential mechanochemical functions of the alternatively spliced exons in the motor domain of *Drosophila melanogaster Mhc1* and those of newly predicted exons in *Daphnia pulex Mhc1* have already been described in detail elsewhere ([4], [14], Figure 2B). Briefly, the MXEs of cluster-1 encode the transition of the N-terminal SH3-like domain to the myosin motor domain, have been shown to be highly conserved between arthropods [4], and influence the maximum power generation [6]. Except for *Daphnia*, cluster-2 (coding for the P-loop, the subsequent α-helix and loop-1) has been described as alternatively spliced exon in the scallops *Argopecten irradians* [15] and *Placopecten magellanicus* [16] although genomic sequence data is only available for the coiled-coil tail region of *Argopecten*. *Argopecten* and *Placopecten* have also been shown to contain two MXEs within cluster-3, which comprises the exon following cluster-2 (coding for the region from the end of loop-1 until the end of switch-1). By alternative encoding of clusters-2 and -3 the entire region from the P-loop over loop-1 to switch-1 can be adjusted (Figure 2B). However, the main differences between the scallop MXEs are in the loop-1 coding region that has been shown to effect ADP release kinetics [17]–[19]. Longer loop-1 regions lead to higher ADP release rates and an increase in actin sliding velocity. The annelids contain an annelid-specific new cluster of MXEs: cluster-4 (Figure 2B), which encodes a central part of the upper 50 kDa domain. To our knowledge, mutants within the cluster-4 region have not been studied so far. The region encoded by cluster-5 MXEs seems to affect muscle fiber kinetics [20]. The region of the motor domain encoded by cluster-6 MXEs has not been investigated so far and therefore functional consequences of differences in the two variants cannot be drawn. Loop-4 has been postulated to be important for the proper localization of class-I myosins containing elongated loops that might sterically interact with actin-binding proteins [36]. However, the loop-4 sequences of the *Daphnia Dap*Mhc1 and *Capitella Cpt*Mhc1 cluster-6 variants are almost identical implying that the MXEs modulate a different property of the motor domain. The MXEs of cluster-7 encode the relay-helix and relay-loop, which transform the movement of switch-2 into the rotation of the converter and the lever arm [21], [22]. The region encoded by cluster-8 MXEs comprises the C-terminal part of loop-2 and the beginning of the subsequent α-helix (Figure 2B). Studies of the *Dictyostelium discoideum* class-2 myosin with its loop-2 replaced by the analogous loop from four other myosins with different enzymatic activities showed that loop-2 is involved in the weak and the strong binding interactions with actin [23]. It also plays an important role in the rate-limiting step of P_i_ release [24], [25]. The MXE cluster-9 that was unique to *Daphnia* so far [4] has been identified in many other lophotrochozoan and arthropod *Mhc* genes here. The region encoded by cluster-9 has, to our knowledge, not been investigated so far. The converter domain region encoded by MXE cluster-10 has been shown to influence the base ATPase activity and actin sliding velocity [9]. Cluster-11 locates to a hinge region in the coiled-coil tail and has been proposed to influence sarcomere lengths by forming a stable or less stable coiled-coil region [26]. The two MXEs are highly conserved between the protostomes with exon type A (5′ exon of the cluster) and type B correlating with fast and slow muscle physiological properties, respectively.

### MXE in *Mnemiopsis leidyi Mhc* genes (ctenophore)

**Figure 3:**
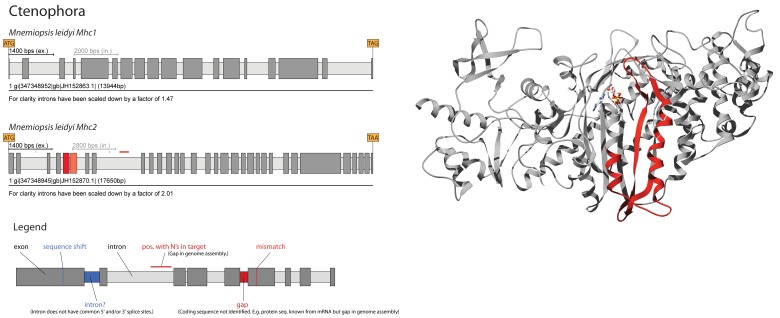
The ctenophore *Mnemiopsis leidyi* contains two muscle *Mhc* genes (left side) of which one contains a cluster of MXEs. Exons and introns are represented as dark- and light-grey bars, respectively, MXEs are shown in colour. The opacity of the colour of the 3′ of the alternative exons corresponds to the alignment score of the alternative exon to the original one (5′ exon). A legend is given explaining the colour coding of features within the gene structure schemes. On the right side, the structural region covered by the MXEs is shown mapped onto the crystal structure of the motor domain of the *Dictyostelium discoideum* non-muscle myosin protein [59].

Ctenophores are thought to form a sister-group to the bilateria, either separate to the cnidarians or together with the cnidarians forming a coelenterate clade [27]. A recent analysis of the cydippid ctenophore *Pleurobrachia pileus* revealed three paralogous class-II myosin genes of which one grouped to the non-muscle genes and the other two grouped as cluster of gene duplicates to the muscle myosin genes [28]. The draft genome of *Mnemiopsis leidyi*, the only ctenophore sequenced so far, also contains three *Mhc* genes with two grouping to the muscle *Mhc* genes (Figure 1 and Figure 3). The *Mhc1* gene corresponds to the *Pleurobrachia* “*PpiMHCIIb1*” gene and the *Mhc2* gene is the ortholog of “*PpiMHCIIb2*”, which is only present as short C-terminal fragment in the available EST data. Localization studies suggest that “*PpiMHCIIb2*” has strictly non-muscular expression [28]. This is very difficult to interpret, as this would be the only *Mhc* gene of the striated muscle *Mhc* gene branch not being present in muscle structures. Because the “*PpiMHCIIb2*” gene fragment only covers some part of the coiled-coil tail domain it is not known whether this gene also contains a cluster of MXEs coding for part of the motor domain like the orthologous *Mhc2* gene from *Mnemiopsis* (Figure 3). The MXEs code for the region starting within the α-helix after the P-loop, covering loop-1 and switch-1, and ending with the loop succeeding the following β-strand. The main differences between the translations of the two MXEs are in loop-1, which is nine residues longer in the 3′ exon, and the short loop after the β-strand. As indicated above, loop-1 is influencing access to the nucleotide-binding site with longer loops leading to lower ADP affinities. Thus, the two *Mhc2* isoforms are predicted to show remarkably different ADP release rates while the remaining mechanochemical properties like actin-binding or the potential size of the power stroke are unaffected.

### MXEs in lophotrochozoan *Mhc* genes

**Figure 4:**
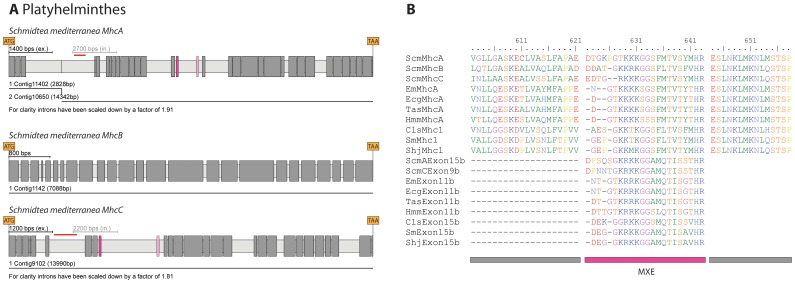
Examples of Platyhelminthes *Mhc* genes. A) The freshwater planarian *Schmidtea mediterranea* contains three *Mhc* genes. In all gene structure schemes exons and introns are represented as dark- and light-grey bars, MXEs are shown in colour. The opacity of the colour of the 3′ of the alternative exons corresponds to the alignment score of the alternative exon to the original one (5′ exon). B) Sequence alignment of the myosin proteins of the analysed Platyhelminthes around the loop-2 region. The part of loop-2, which is encoded by MXEs, is indicated. The sequences of the 5′ exons are very similar across the Platyhelminthes, as are the 3′ exons, implying that the ancestor of the Platyhelminthes already contained this cluster of MXEs.

The Platyhelminthes *Hymenolepsis*, *Echinococcus*, *Taenia*, *Schmidtea*, *Clonorchis*, and *Schistosoma* contain two MXEs in cluster-8 of their *Mhc* genes (Figure 4A, Figure S2). Across the species, these two exons are highly conserved implying that the last common ancestor of the Platyhelminthes already had this cluster of MXEs. The exons of cluster-8 encode different versions of loop-2 [4], which comprises an important part of the actin-binding site, and the Platyhelminthes can thus express muscle myosins with modulated actin-binding properties. So far, only muscle myosins of the cestode parasite *Taenia solium* have been investigated biochemically [29], [30]. *Taenia* exists in two developmental stages, cysticerci (larvae) and tapeworms (adults). Myosins were extracted from both stages and their ATPase activity determined in the presence of actin showing a higher activity in the tapeworm sample [29]. These experimental results can now be interpreted in terms of the sequence data. The sequence data suggest.two myosin isoforms with different loop-2 regions and thus different actin-activated ATPase activity. In addition, the experimental data indicates that the inclusion of the MXEs into the final transcript is developmentally regulated in Platyhelmintes. Proposed additional smaller isoforms in the experimental study [29] are most probably artefacts from proteolysis. The transcript sequence determined from a muscle myosin from adult *Schistosoma mansoni* [31] is identical to the sequence derived from genomic DNA as reported here. Mutually exclusive exon A (5′ exon of the cluster) is included in this sequence implying that exon B is the version spliced into the larval *Mhc* transcript. The sequence similarity of the MXEs of the Platyhelminthes *Mhc* genes (Figure 4B) suggests that the MXE-splicing in *Schistosoma* can be transferred to *Taenia* and accounts for all Platyhelmintes. The Platyhelmintes *Mhc* isoforms including exon B (3′ exon of the cluster) would thus be the isoforms with the lower ATPase activity. The freshwater planarian *Schmidtea mediterranea* (*Scm*) is different to the other Platyhelminthes as its genome contains three different muscle *Mhc* genes, of which two contain MXE cluster-8 (Figure 4A). The three genes are not ordered in tandem in the genome, but *ScmMhcA* and *ScmMhcC* are closely related (Figure 1) and therefore most probably the result of a recent gene duplication.

**Figure 5:**
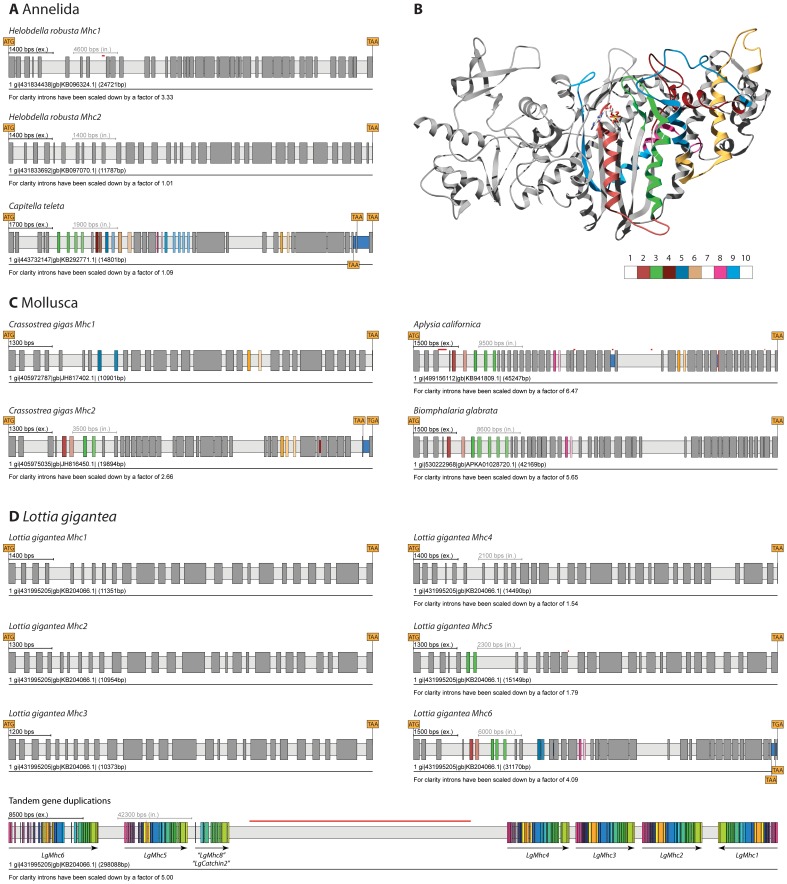
Examples of annelid and mollusc *Mhc* genes, and location of the MXE coding regions within the motor domain. A) The annelid *Helobdella robusta* contains two *Mhc* genes without any clusters of MXEs, while the annelid *Capitella teleta* contains one *Mhc* gene with many clusters of MXEs and three differentially included exons at the C-terminus. B) The structural regions covered by MXEs present in lophotrochozoans are shown mapped onto the crystal structure of the motor domain of the *Dictyostelium discoideum* non-muscle myosin protein [59]. C) Examples of representative mollusc *Mhc* genes showing the divergence in MXE clusters in the respective subphyla. D) Gene structures of the muscle *Mhc* genes in the owl limpet *Lottia gigantea*. The scheme at the bottom shows the genomic region of the cluster of *Mhc* genes including the *Mhc8* gene that encodes only part of the coiled-coil tail region. Reading direction is designated by arrows. Colours of exons in the *Mhc* gene cluster represent exons coding for a similar part of the protein. In all gene structure schemes exons and introns are represented as dark- and light-grey bars, MXEs are shown in colour. The opacity of the colour of the 3′ of the alternative exons corresponds to the alignment score of the alternative exon to the original one (5′ exon). The vertical red line in the genomic region scheme at the bottom represents a region of unknown sequence (“N”s). The complete list of lophotrochozoan *Mhc* genes is shown in Figure S2.

Two annelids have been sequenced so far, the freshwater leech *Helobdella robusta* [32] and the marine polychaete *Capitella teleta* [32]. *Helobdella* contains two muscle myosin heavy chain genes, which both do not contain any clusters of MXEs (Figure 5A). They are not organized in tandem but are most probably the result of a species-specific or leech branch-specific gene duplication after the ancient gene lost the MXE clusters. In contrast, the *Capitella Mhc* gene contains seven clusters of MXEs and three differentially included C-terminal exons (evidence by EST data; Figure 5A) providing the potential for many alternatively spliced transcripts. The MXEs are distributed in clusters-3, -4, -5, -6, -8, -9, and -11. So far, the *Capitella* MXE cluster-9 is the only cluster-9 with more than two MXEs. The cluster-9 exons encode a β-strand of the central β-sheet of the motor domain (Figure 5B). In addition, the *Capitella Mhc* contains a so far unique cluster, cluster-4, which is part of the upper 50 kD domain.

The sequenced molluscs show a broad variety of *Mhc* genes from single genes in the California sea hare *Aplysia californica* (Figure 5C) to clusters of *Mhc* genes in the owl limpet *Lottia gigantea* (Figure 5D). The Pacific oyster *Crassostrea gigas* (Bivalvia clade) contains two *Mhc* genes with different sets of clusters of MXEs. The *Mhc1* gene contains clusters-5 and -11, and the *Mhc2* gene includes clusters-2, -3, and -11, of which the cluster-11 is the only cluster-11 so far with more than two MXEs. The ancestral bivalvian *Mhc* gene must have had the combination of the clusters of the two *Mhc* genes, and different MXEs had subsequently been lost in the duplicated *Mhc* genes. The catch and striated adductor muscle *Mhc* isoforms of the bay scallop *Argopecten irradians* and the sea scallop *Placopecten magellanicus* have been sequenced [15], [16]. These transcripts contain the MXE cluster combinations 2a, 3b, 11b (isoform A, catch muscle) and 2b, 3a, 11a (isoform B, striated muscle), which can also be generated by alternative splicing of the *Crassostrea CagMhc2* gene (Figure 5C). For *Crassostrea* a cDNA library generated from mixed adult tissues is available. Several clones code for the MXE combination 2b, 3b, while only a single clone is available for the 2b, 3a combination and none for the combination 2a, 3b. However, most cDNA clones cover the *Mhc1* gene, which therefore seems to be the ubiquitously expressed isoform in *Crassostrea*.

The gastropods *Aplysia* and *Biomphalaria glabrata* (a neotropical snail) contain single *Mhc* genes with MXEs in clusters-2, -3, -8, and -11, and clusters-2, -3, and -8, respectively (Figure 4). *Lottia* (gastropod) contains an extended array of seven *Mhc* genes arranged in tandem, of which *Mhc8* only codes for the coiled-coil tail region of a myosin (Figure 5D). Expression of *Mhc8* is supported by many EST clones and the gene starts exactly at the same position where the alternatively spliced scallop *Mhc* isoform catchin begins [33]. However, the catchin isoforms contain a long unique N-terminal exon, that is present in *Aplysia Mhc1*, *Biomphalaria Mhc1*, *Crassostrea Mhc2*, and *Lottia Mhc6* but not present in *Lottia Mhc8* (Figure S3). Similar to catchin, a so-called myosin rod protein has been identified in *Drosophila melanogaster* as result from an alternative transcript of the myosin coiled-coil tail region [34]. This myosin rod protein is about 260 residues longer than catchin and formed by an alternative start site to the first exon following the myosin motor and light-chain binding domains (exon 12 in *D.melanogaster*). In contrast to the catchin proteins the N-termini of the myosin rod proteins are not even conserved between the *Drosophila* species and their closest relatives, the mosquitoes, or within other closely related species. For example, in the beetles *Tribolium castaneum* and *Dendroctonus ponderosae* the 5′ extensions to exon 17 and exon 18, respectively, which would correspond to the *D.melanogaster* myosin rod protein, would be 16 and 101 residues. As long as mRNA or other experimental data is missing for any myosin rod protein homolog to the *D.melanogaster* protein, these isoforms cannot reliably be predicted. The *Lottia Mhc1* gene is encoded in the opposite direction to the other genes of the cluster. The *Mhc6* gene includes MXEs in clusters-2, -3, -5, and -8, and contains three differentially included C-terminal exons (evidence by EST data). The *Mhc5* gene contains two MXEs in cluster-3, and the remaining *Mhc* genes do not have any clusters of MXEs. This is in agreement with our phylogenetic analysis (Figure 1) that shows that the *Mhc6* gene is the most ancient gene of the cluster followed by the *Mhc5* and *Mhc4* genes. Every duplicated gene in the tandem array of *Mhc* genes lost clusters of MXEs (from *Mhc6* to *Mhc5* and *Mhc4*) and introns (from *Mhc6* to *Mhc5*, from *Mhc4* to *Mhc3*, and from *Mhc2* to *Mhc1*). Five muscle tissues of mollusc from a different sub-branch, the squid *Doryteuthis pealeii* (Cephalopda clade) have been studied [35]. Although the ultrastructure and contractile properties of these tissues are significantly different, they all contain the same three myosin isoforms. These isoforms differ in the C-terminus and by the region covered by MXE cluster-3. Because both cluster-3 isoforms are present in the muscle tissues it has been argued that differences in ultrastructure and not myosin ATPase activity are crucial for tuning contractile speed in *Doryteuthis* [35]. However, different average ATPase activities could also be achieved by differences in the relative levels of the isoforms, which could control contractile properties in different muscles. Apart from tuning myosins by alternative splicing or gene duplications there might therefore be additional mechanisms triggering muscle ultrastructure and performance.

### MXEs in Chelicerata (Arachnida) and Chilopoda *Mhc* genes

**Figure 6:**
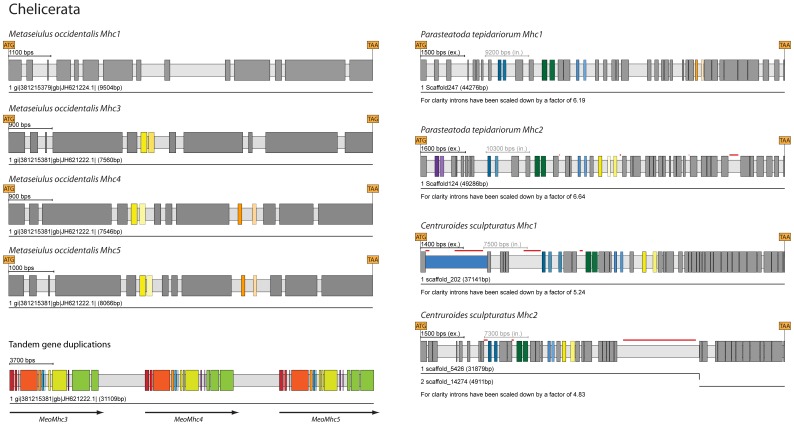
The schemes represent examples of *Mhc* genes in Chelicerata. *Metaseiulus occidentalis* contains four *Mhc* genes of which the ones with MXE clusters are arranged as tandem array of gene duplicates (*Mhc3*, *Mhc4* and *Mhc5*). *Parasteatoda tepidariorum* and *Centruroides sculpturatus* both contain two *Mhc* genes. The complete list of Chelicerata *Mhc* genes is shown in Figure S2. Exons and introns are represented as dark- and light-grey bars, MXEs are shown in colour. The opacity of the colour of the 3′ of the alternative exons corresponds to the alignment score of the alternative exon to the original one (5′ exon).

The centipede *Strigamia maritima* is the only Chilopoda sequenced so far and its *Mhc* gene contains all arthropod MXE clusters except clusters-2, -6, and -11. The sequenced Chelicerata include the red spider mite *Tetranychus urticae*, the deer tick *Ixodes scapularis*, the predatory mite *Metaseiulus occidentalis*, the common house spider *Parasteatoda tepidariorum*, and the scorpion *Centruroides sculpturatus* (Figure 6, Figure S2). The Chelicerata *Mhc* genes are characterised by many but small clusters of two to three MXEs. The *Tetranychus Mhc* gene contains two MXEs in each of the clusters-5, -7, -9, -10, and -11. The *Ixodes Mhc* gene in addition contains clusters-1 and -2. *Metaseiulus* contains four *Mhc* genes (*Mhc1*, *Mhc3*, *Mhc4*, and *Mhc5*), of which three include clusters of MXEs (Figure 6). The *Mhc3*, *Mhc4*, and *Mhc5* genes are organized in tandem and most probably appeared by recent gene duplications. *Mhc4* and *Mhc5* contain clusters-10 and -11, while *Mhc3* only contains cluster-10. *Parasteatoda* and *Centruroides* each contain two *Mhc* genes together forming two distinct subclasses (Figure 1). Although the *Mhc* genes of *Parasteatoda* and *Centruroides* are closely related they encode different types of clusters. The *Parasteatoda Mhc1* contains two MXEs in clusters-5, -7, -9, and -11, while the *Centruroides Mhc1* contains three MXEs in cluster-5 and two MXEs in clusters-7, -9, and -10 (Figure 6). EST data from tarantula skeletal muscle tissue have been obtained [36] but the assembled EST contigs were too fragmented to reveal the total number of *Mhc* genes although alternative transcripts were detected.

### MXEs in crustacean *Mhc* genes

Crustacea are a sister group to Hexapoda (Figure 1). The *Daphnia pulex* (branchiopoda branch) *Mhc* gene contains MXE clusters-1 and -2, and clusters-5 to -11, and has been described in detail elsewhere [4] (Figure S2). The other crustacean species analysed is the salmon louse *Lepeophtheirus salmonis* (copepod branch) that contains 17 muscle myosin heavy chain genes without any clusters of MXEs (Figure S2). These myosins split into two major groups of seven (*Mhc10 - Mhc16*) and nine isoforms (*Mhc1 - Mhc9*), and a more distant homolog (*Mhc17*, Figure 1). Recently, the draft genome of another copepod, the calanoid *Eurytemora affinis*, became available, which contains a similar amount of muscle myosin heavy chain genes without MXEs (data not shown). This implies that the last common ancestor of the copepods must have developed an MXE-less muscle myosin heavy chain gene followed by extensive gene duplications. Multiple *Mhc* genes have experimentally been found in shrimps [37]–[39] and gammarid amphipods [40] and some could be obtained in full-length (Figure 1). These group closer to the *Lepeophtheirus Mhc* genes than to the *Daphnia Mhc1* implying that encoding of multiple, but not alternatively spliced *Mhc* genes is a common characteristic of many crustaceans.

### MXEs in insect *Mhc* genes

**Figure 7:**
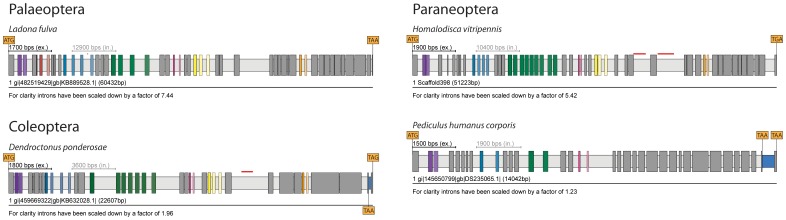
Examples of *Mhc* genes of Palaeoptera, Coleoptera and Paraneoptera. The examples have been chosen because of the unusual combinations of clusters of MXEs or because of unusual high or low numbers of MXEs within clusters. The complete list of arthropod *Mhc* genes is shown in Figure S2. Exons and introns are represented as dark- and light-grey bars, MXEs are shown in colour. The opacity of the colour of the 3′ of the alternative exons corresponds to the alignment score of the alternative exon to the original one (5′ exon).

Within the Insecta, genome assemblies are only available for species of Pterygota, which branches into Palaeoptera and Neoptera (Figure 7 and Figure S2). The insects lost MXE cluster-9 compared to Crustacea. MXE cluster-2 is currently restricted to the Palaeoptera (Figure 7) implying that it had been lost in the ancestor of the Neoptera. In the Neoptera branch, genome assemblies are now available for species of the geni Paraneoptera, Amphiesmenoptera (Lepidoptera and Trichoptera), Coleoptera, Diptera, Hymenoptera, and Strepsiptera that all contain MXE clusters-1, -5, -7, -10, and -11. Between clusters-7 and -10 there are five exons in the ancient insect gene, of which the middle exon is often mutually exclusive spliced (cluster-8). In Diptera, all five exons are fused to a single exon. In Hymneoptera, the last four exons are fused, and in Strepsiptera the first two and the last three are fused (Figure S2). Therefore, cluster-8 is missing in these genes. The Paraneoptera and Amphiesmenoptera have the five exons including MXE cluster-8, while either or both of the neighbouring exons of MXE cluster-8 are fused in the various Coleoptera. Based on the molecular phylogeny of the species (Figure 1) this implies that this full set of MXEs (clusters-1, -5, -7, -8, -10, and -11) must have been present in the last common ancestor of the Neoptera and independently been lost in Hymenoptera, Diptera, and Strepsiptera in the course of exon fusion events. Extensive exon fusions have already been reported for arthropod *Mhc* genes [4]. The Neoptera have two MXEs in clusters-1 and -11, and, in general, three or four MXEs in cluster-5, three to six MXEs in cluster-7, and three to five MXEs in cluster-10. Exceptions are the mountain pine beetle *Dendroctonus ponderosae* and the glassy-winged sharpshooter *Homalodisca vitripennis Mhc* genes that show the highest complexity having seven and nine MXEs in cluster-7, respectively, and the human body louse *Pediculus humanus corporis Mhc* gene that has the lowest complexity with only two MXEs in cluster-5 and missing cluster-10 (Figure 7).

### MXEs in deuterostomian *Mhc* genes

Three genomes are available from Echinodermata, which are all from sea urchins, and the genome of the acorn worm *Saccoglossus kowalevskii* that belongs to the Hemichordata (Figure 1 and Figure S2). These species each contain two MXEs within cluster-8. Both versions in *Strongylocentrotus purpuratus* and *Saccoglossuus kowalevskii* are supported by EST data.

### Evolution of the metazoan MXE containing *Mhc* genes

**Figure 8:**
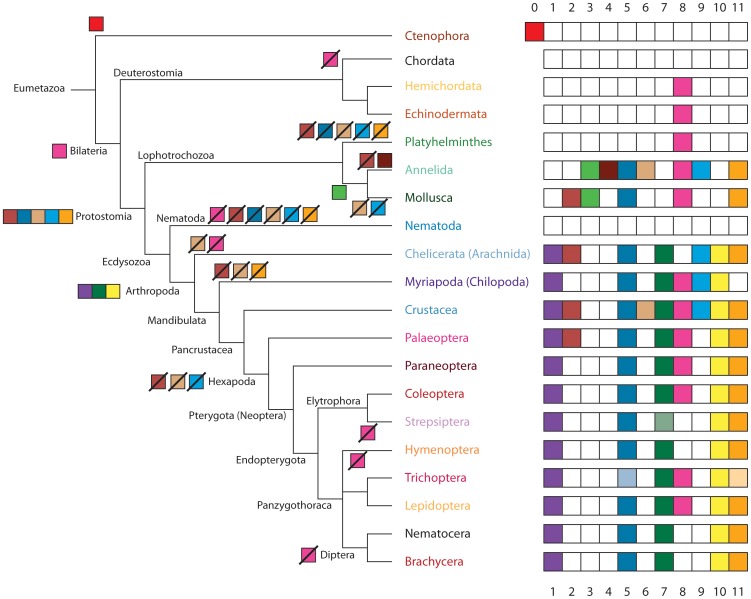
Schematic drawing of the evolution of the clusters of MXEs within eumetazoan *Mhc* genes. The only Ctenophora sequenced, *Mnemiopsis leidyi*, contains a cluster of MXEs that does not correspond to any other known cluster and has therefore been named cluster-0. The tree is shown as schematic tree representing known phylogenetic relationships to which MXE cluster loss and gain events were plotted. MXE clusters were regarded as gained in the last common ancestor of the branch, which contains species encoding these clusters. According to this scheme, five clusters have evolved in the last common ancestor of the Protostomia, and a set of three clusters later at the onset of the arthropods. There are many branches and species that completely lost all clusters of MXEs in their *Mhc* genes. Coloured boxes represent MXE cluster gain events (tree view, left side) and their presence within a certain branch (table, right side). Crossed boxes denote MXE cluster loss events. MXEs in light-colour symbolize clusters of MXEs that were supposed to be present but could not be approved because of genome assembly gaps (Figure S2).

Previously, it has been thought that there are mainly two possibilities for a species to provide different muscle myosin heavy chain genes for the different muscle types: the species could either express a set of separate *Mhc* genes or have a single gene but generate different *Mhc* transcripts by alternative splicing of mutually exclusive exons. Sets of *Mhc* genes have been found in the nematode *Caenorhabditis elegans*, the tunicate *Ciona intestinalis*, and vertebrates [13], [41], and single genes with complex patterns of clusters of MXEs covering half of the motor domain have been identified in arthropods [4]. Here, we could show that sets of *Mhc* genes are not restricted to nematodes and chordates and that *Mhc* genes with MXEs are not only found in arthropods. Instead, large sets of *Mhc* genes are found for example in crustaceans and molluscs, and MXEs have been predicted in all bilateria except chordates, and even in a sequenced ctenophore (Figure 8). Also, there are species that contain several *Mhc* genes like *Crassostrea gigas*, *Helobdella robusta*, *Lottia gigantea* and *Lepeophtheirus salmonis*. In addition, several or all of these duplicated *Mhc* genes can include clusters of MXEs, and the set of MXE clusters can either be identical or different in the duplicated genes.

To trace the evolution of MXE clusters within the bilateria we regarded every cluster of MXEs present in two species as also present in the last common ancestor of these species. This excludes the possibility that the respective cluster of MXEs could have also appeared independently in several branches. However, as all clusters except cluster-4 are present in many species from different branches a common origin is far more likely than an independent invention. Most bilateria have cluster-8 of MXEs, which therefore most probably first appeared in the last common muscle *Mhc* gene of the bilateria (Figure 8). At the onset of the Protostomia, five further clusters of MXEs, clusters-2, -5, -6, -9, and -11, have been introduced, that have subsequently been lost in the Platyhelminthes. Many analyses have shown that the phylum Platyhelminthes groups close to the Annelida and the Mollusca within the Lophotrochozoa [42], [43] although is seems unreasonable that the ancient Platyhelminthes immediately lost the five clusters of MXEs, which were just shortly derived before. However, it must have been a strong selective advantage for the ancient protostome to have many of the exons coding for the motor domain duplicated forming an extensive set of MXEs. Similarly to the Platyhelminthes, the nematodes lost all clusters of MXEs and instead developed sets of different *Mhc* genes. The ancestor of the arthropods duplicated further exons resulting in three further clusters of MXEs (Figure 8). During the subsequent evolution of the arthropods, several of the MXEs got lost independently in many sub-branches. Only the water flea *Daphnia pulex* has retained the full set of MXE clusters.

Based on the data presented here it seems that all clusters of MXEs evolved early in metazoan evolution, namely in the ancestor of the protostomes, the ancestor of the arthropods, and the last common ancestor of the annelids and molluscs. The subsequent evolution in all bilateria is characterised by branch-specific MXE cluster loss events, which happened through loss of alternative exons or by fusion of previously alternative exons with neighbouring constitutive exons. While the emergence of clusters of MXEs can be traced back to the early bilateria, the expansion of already existing clusters has been shown to be, at least in part, specific to recent branches and extant species [4]. The number of clusters together with the number of MXEs within clusters and, in many species, different *Mhc* genes allow for a wealth of expressed myosin proteins adapted to all kinds of muscle tissues. It is well known from *Drosophila* that not all possible combinations of MXEs are realized and this might also be true for the other bilateria but it seems likely that many combinations are in fact expressed although not experimentally confirmed yet. However, because most studies have focused on major muscle tissues so far, improved experimental tissue separation techniques together with single-cell sequencing are expected to reveal the entire complexity of myosin transcripts in animals.

## Materials and Methods

### Identification and annotation of the myosin heavy chain genes

The myosin heavy chain gene data from the 22 arthropods available in 2007 were obtained from [4]. The sequences were updated based on newer genome assemblies if necessary. The other myosin genes have essentially been obtained as described in [13]. Shortly, myosin genes have been identified in TBLASTN searches starting with the protein sequence of the *Drosophila melanogaster* muscle myosin heavy chain. The respective genomic regions were submitted to AUGUSTUS [44] to obtain gene predictions. However, feature sets are only available for a few arthropod species. Therefore, all hits were subsequently manually analysed at the genomic DNA level. When necessary, gene predictions were corrected by comparison with the other myosins as included in the multiple sequence alignment. Where possible, EST data have been analysed to help in the annotation process.

In the last years, genome sequencing efforts have been extended from sequencing species from new branches to sequencing closely related organisms. Here, these species include for example seven ant species, 23 *Drosophila* species, and eleven species of the *Anopheles* genus. Protein sequences from these closely related species have been obtained by using the cross-species functionality of WebScipio [45], [46]. Nevertheless, also for all these genomes TBLASTN searches have been performed. With this strategy, we sought to ensure that we would not miss more divergent myosin homologs, which might have been derived by species-specific inventions or duplications. Gene duplicates have previously been identified in *Aedes aegypti* and *Culex pipiens* [4], and were identified here in for example *Metaseiulus occidentalis*, *Helobdella robusta*, and *Lottia gigantea*.

The annotated protein sequences were subsequently used to detect mutually exclusive spliced exons by using an algorithm implemented in WebScipio [5]. Default options were used for the specificity of the prediction (length difference  =  20 aa, minimal score  =  15%). Because muscle myosin genes contain short exons, especially one spanning loop-2 and being mutually exclusive spliced in known examples [4], the search space was increased to smaller exons (minimal exon length  =  10 aa). The search was restricted to internal and surrounding MXEs. Tandem arrangement of gene duplicates was determined by gene locations on contigs and reconstructed using a plugin implemented in WebScipio [47].

All sequence related data (protein names, corresponding species, sequences, and gene structure reconstructions) and references to genome sequencing centres are available at CyMoBase (http://www.cymobase.org, [48]). A list of the analysed species, their abbreviations as used in the alignments and trees, as well as detailed information and acknowledgments of the respective sequencing centres are also available as Table S1. WebScipio [45], [46] was used for reconstruction and visualization of the gene structure (i.e. the exon/intron pattern including clusters of MXEs) of each sequence.

### Generating the multiple sequence alignment

The muscle myosin heavy chain sequences were added to the structure-guided multiple sequence alignment obtained from [4]. In detail, we first aligned every newly predicted sequence to its supposed closest relative using ClustalW [49] and added it then to the multiple sequence alignment. During the subsequent sequence validation process, we manually adjusted the obtained alignment by removing wrongly predicted sequence regions and filling gaps. Still, in those sequences derived from low-coverage genomes many gaps remained. To maintain the integrity of exons preceded or followed by gaps, gaps reflecting missing parts of the genomes were added to the multiple sequence alignment. The sequence alignment is available from CyMoBase or Dataset S1.

### Computing and visualising phylogenetic trees

As outgroup, non-muscle class II myosin sequences from *Schizosaccharomyces octosporus, Schizosaccharomyces cryophilus, Schizosaccharomyces pombe*, and *Schizosaccharomyces japonicus* were added to the multiple sequence alignment. The phylogenetic trees were generated using four different methods: Neighbour Joining, Maximum likelihood, Bayesian inference and split networks. 1. ClustalW v.2.0.10 [49] was used to calculate unrooted trees with the Neighbour Joining method. For each dataset, bootstrapping with 1,000 replicates was performed. 2. Maximum likelihood (ML) analysis with estimated proportion of invariable sites and bootstrapping (1,000 replicates) were performed using RAxML [50]. First, ProtTest v.3.2 was used to determine the most appropriate of the available 120 amino acid substitution models [51]. Within ProtTest, the tree topology was calculated with the BioNJ algorithm and both the branch lengths and the model of protein evolution were optimized simultaneously. The Akaike Information Criterion with a modification to control for small sample size (AICc, with alignment length representing sample size) identified the RtREV model [52] with gamma model of rate heterogeneity and empirical base frequencies to be the best model available in RAxML. 3. Posterior probabilities were generated using MrBayes v.3.2.1 [53]. Using the mixed amino-acid option, two independent runs with 4,000,000 generations, four chains, and a random starting tree were performed. MrBayes used the WAG model [54] for all protein alignments. Trees were sampled every 1.000th generation and the first 25% of the trees were discarded as “burn-in” before generating a consensus tree. 4. An unrooted phylogenetic split network was generated with SplitsTree v.4.13.1 [55]. The NeighborNet method as implemented in SplitsTree was used to identify alternative splits. Phylogenetic trees and networks were visualized with FigTree v.1.3.1 [56], iTOL v.2.2.2 [57] and SplitsTree, respectively, and are available as Figure S1.

## Supporting Information

Figure S1 **Phylogenetic trees.** This file contains the phylogenetic trees. The coloured, circular tree was generated with RAxML and the linear trees were generated with ClustalW, RAxML and MrBayes. Bootstrap support values and posterior probabilities are reported in absolute values (ClustalW) and relative values (RAxML and MrBayes).
(PDF)

Figure S2 ***Mhc*** **gene structure schemes.** This file displays the gene structures including clusters of predicted MXEs for all sequences analysed. Exons and introns are scaled in cases, in which the combined intronic regions are longer than the exons, such that both exons and introns represent half of the total width of the scheme. Two neighboring exons in *Lasioglossum albipes* and *Mayetiola destructor* are identical (red color) but these exons do not belong to the known clusters of MXEs. Either, these exons are derived from sequencing or assembly problems, or represent recent species-specific generations of new clusters of MXEs.
(PDF)

Figure S3 **Detailed gene structure schemes of the lophotrochozoan** ***Mhc*** **genes.** This file displays the gene structures including clusters of predicted MXEs and the alternative N-terminal exons leading to the *Mhc* genes for *Crassostrea*, *Aplysia*, *Biomphalaria*, and *Lottia*. Exons and introns are scaled, such that both exons and introns represent half of the total width of the scheme. Alternative gene start sites (methionines) and stop codons are indicated.
(PDF)

Table S1 **Species names and abbreviations, and references to genome data.**
(XLS)

Dataset S1 **Sequence alignment of the muscle myosin heavy chain proteins.**
(FAS)
