# Supplementary figures and images for: Shared Gene Structures and Clusters of Mutually Exclusive Spliced Exons within the Metazoan Muscle Myosin Heavy Chain Genes
Source: PLoS One. 2014 Feb 3;9(2):e88111. doi: 10.1371/journal.pone.0088111 (PMC3912159; doi:10.1371/journal.pone.0088111)

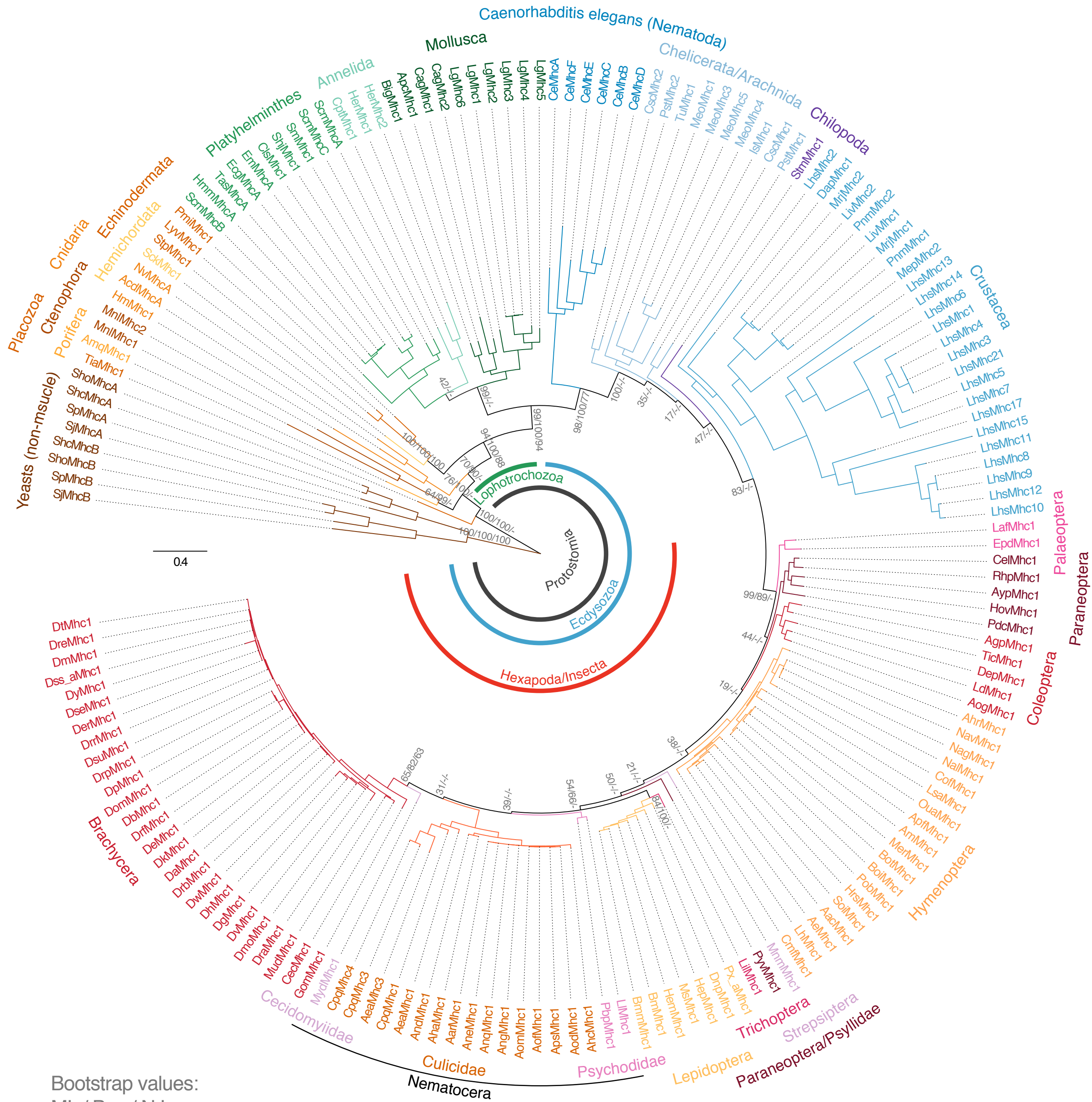

Bootstrap values:  
ML / Bay / NJ

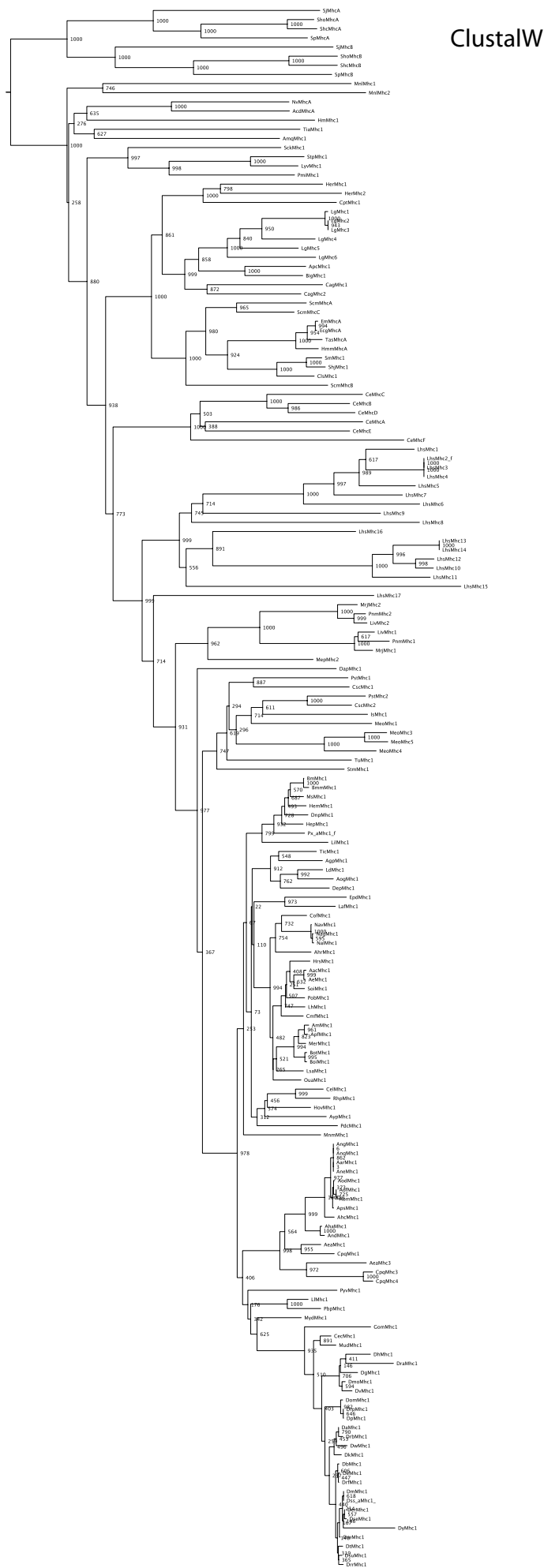

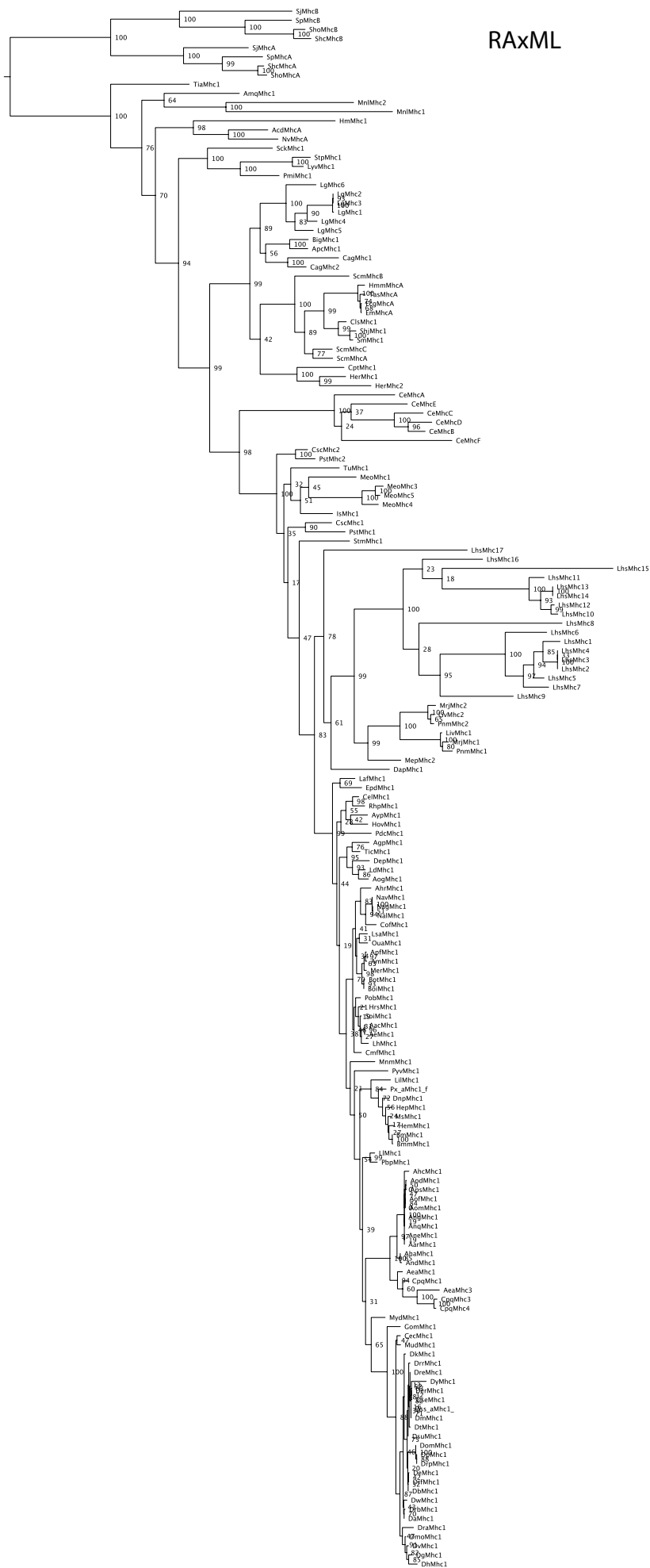

## MrBayes

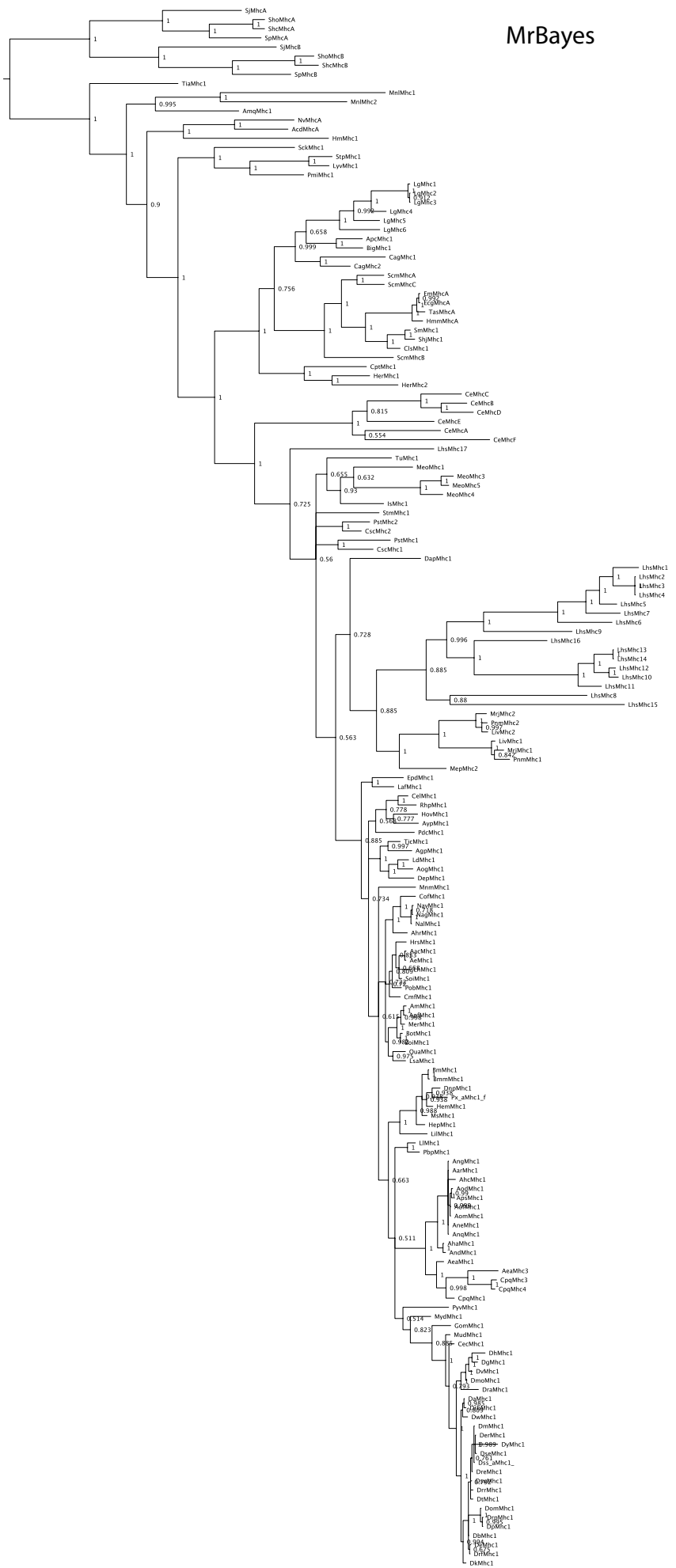

0.2

Supplement: Figure S1 — Phylogenetic trees. This file contains the phylogenetic trees. The coloured, circular tree was generated with RAxML and the linear trees were generated with ClustalW, RAxML and MrBayes. Bootstrap support values and posterior probabilities are reported in absolute values (ClustalW) and relative values (RAxML and MrBayes). (PDF) [file pone.0088111.s001.pdf]
