# Supplementary material for: Shared Gene Structures and Clusters of Mutually Exclusive Spliced Exons within the Metazoan Muscle Myosin Heavy Chain Genes
Source: PLoS One. 2014 Feb 3;9(2):e88111. doi: 10.1371/journal.pone.0088111 (PMC3912159; doi:10.1371/journal.pone.0088111)
Supplement: Figure S2 — Mhc gene structure schemes. This file displays the gene structures including clusters of predicted MXEs for all sequences analysed. Exons and introns are scaled in cases, in which the combined intronic regions are longer than the exons, such that both exons and introns represent half of the total width of the scheme. Two neighboring exons in Lasioglossum albipes and Mayetiola destructor are identical (red color) but these exons do not belong to the known clusters of MXEs. Either, these exons are derived from sequencing or assembly problems, or represent recent species-specific generations of new clusters of MXEs. (PDF) [file pone.0088111.s002.pdf]

Ctenophora

Mnemiopsis leidyi Mhc1

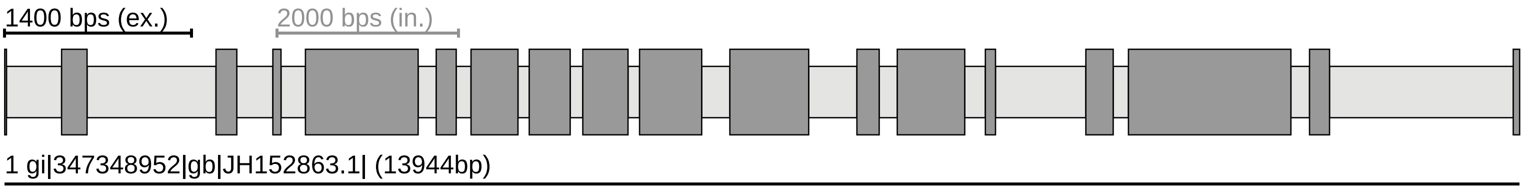

For clarity introns have been scaled down by a factor of 1.47

Mnemiopsis leidyi Mhc2

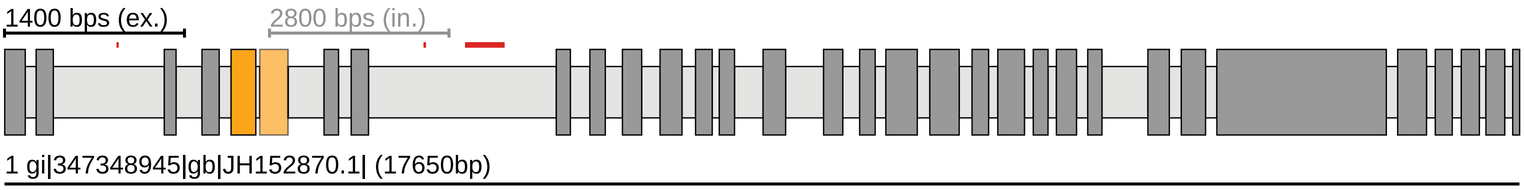

For clarity introns have been scaled down by a factor of 2.01

Echinodermata

Strongylocentrotus purpuratus

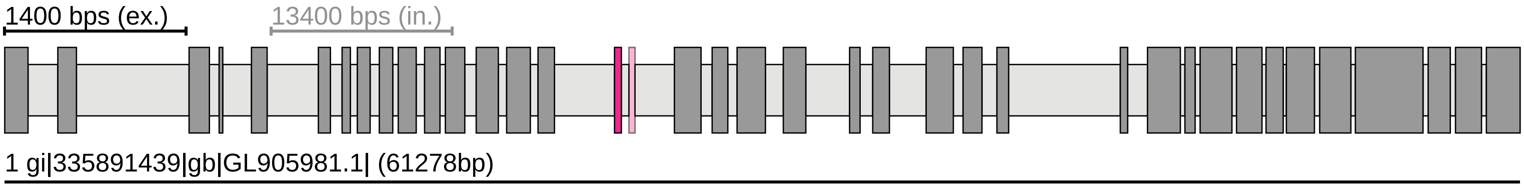

For clarity introns have been scaled down by a factor of 9.58

Patiria miniata

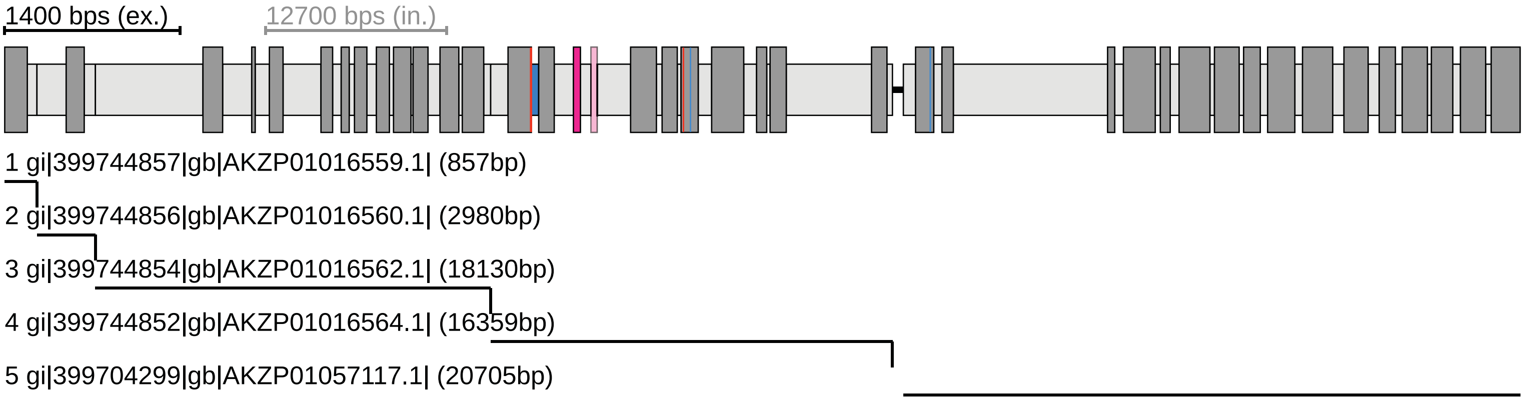

For clarity introns have been scaled down by a factor of 8.80

Lytechinus variegatus

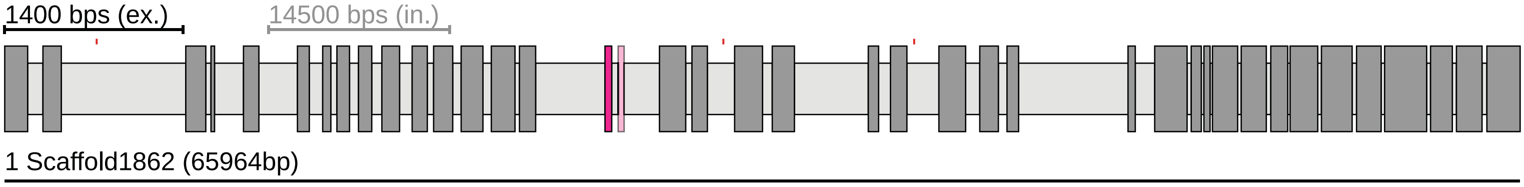

For clarity introns have been scaled down by a factor of 10.21

Hemichordata

Saccoglossus kowalevskii

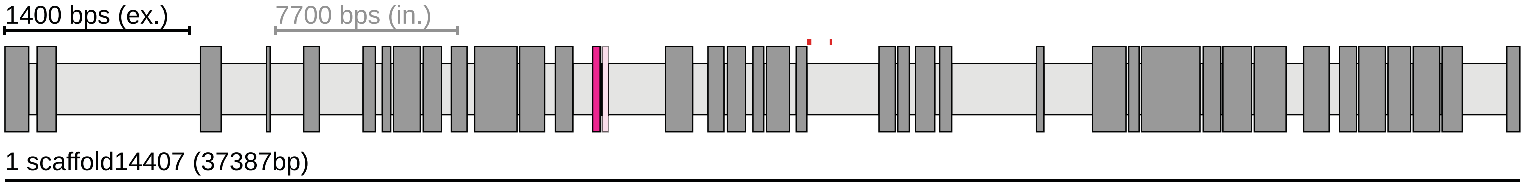

For clarity introns have been scaled down by a factor of 5.58

Platyhelminthes

Hymenolepis microstoma

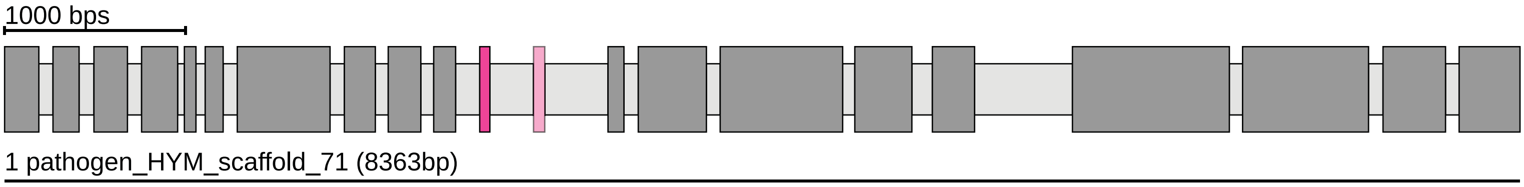

Echinococcus granulosus

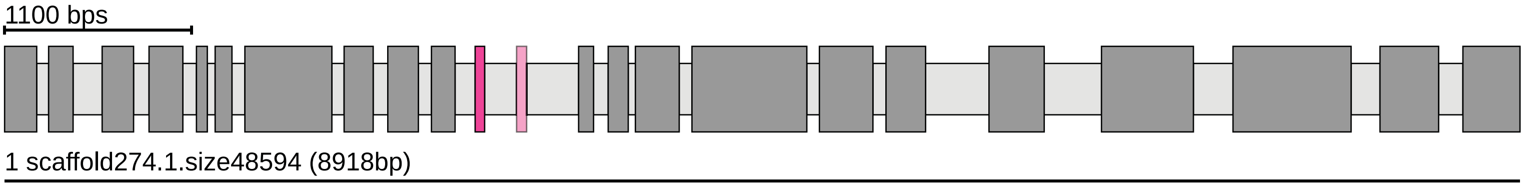

Echinococcus multilocularis

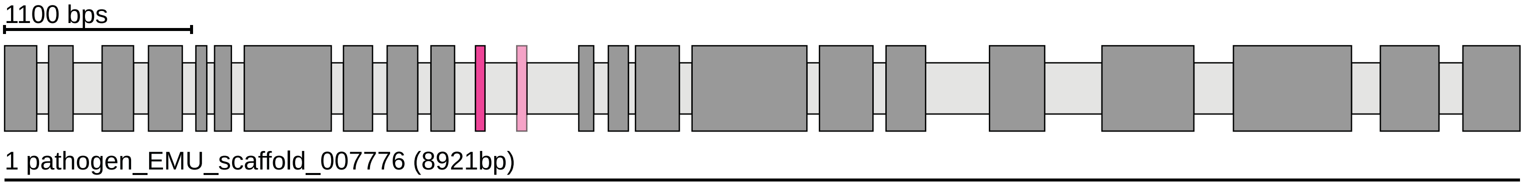

Taenia solium

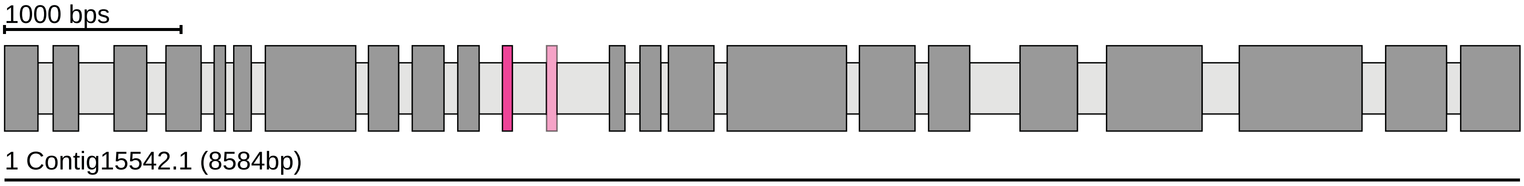

Schmidtea mediterranea MhcA

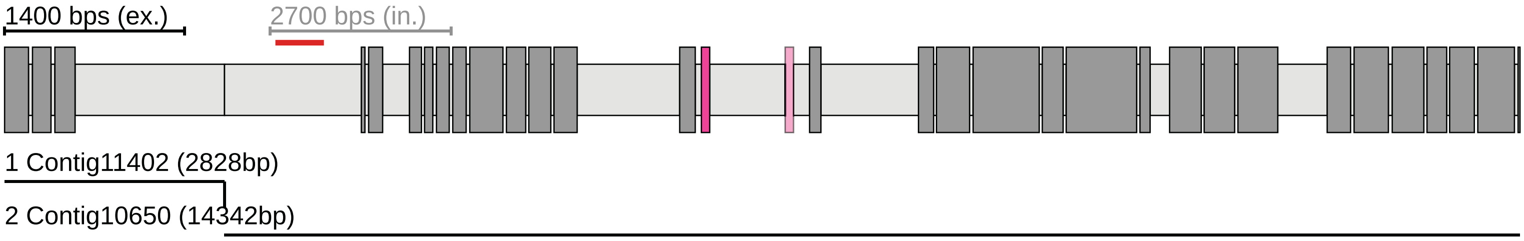

For clarity introns have been scaled down by a factor of 1.91

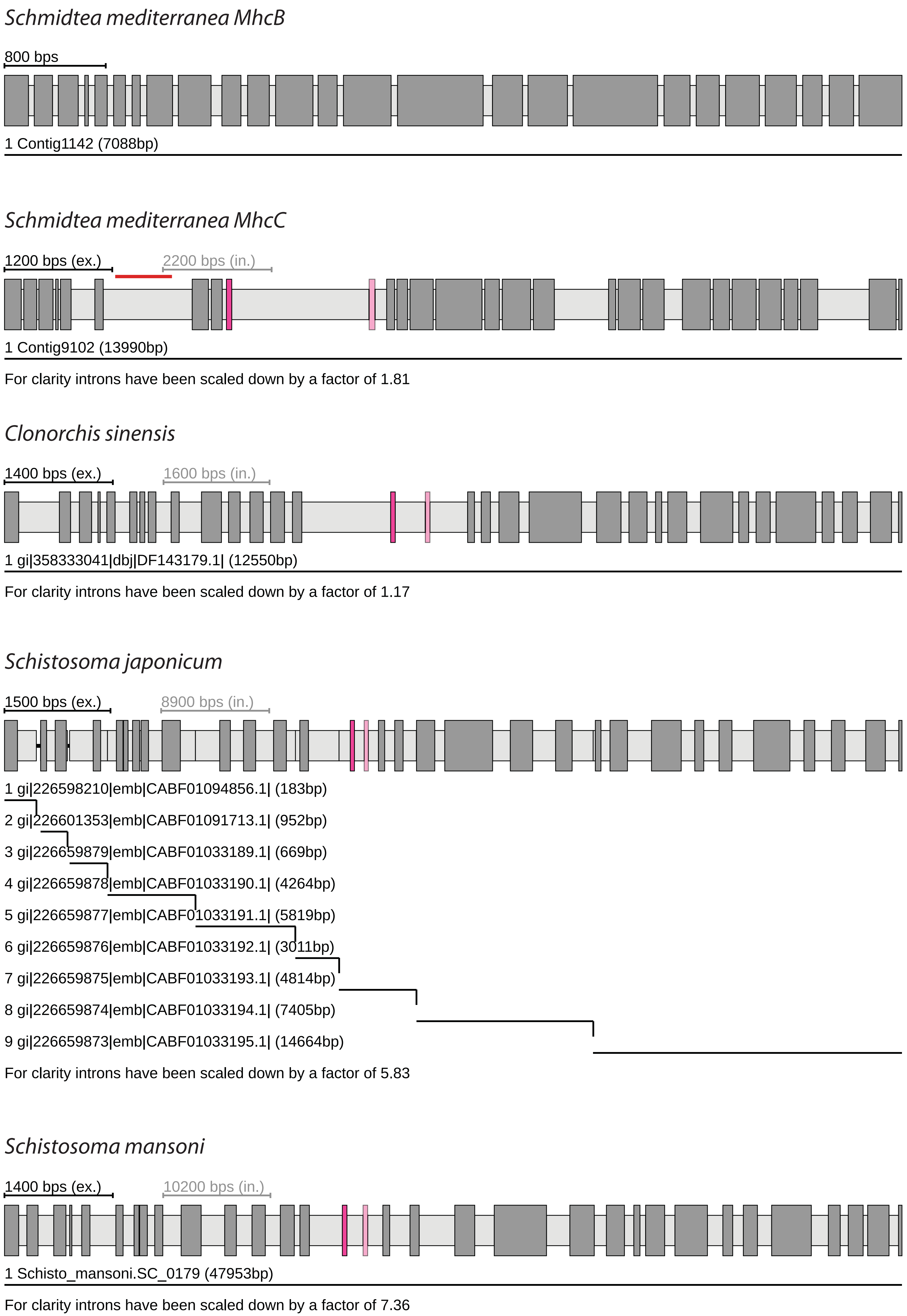
Schmidtea mediterranea MhcB
800 bps
1 Contig1142 (7088bp)
Schmidtea mediterranea MhcC
1200 bps (ex.)
2200 bps (in.)
1 Contig9102 (13990bp)
For clarity introns have been scaled down by a factor of 1.81
Clonorchis sinensis
1400 bps (ex.)
1600 bps (in.)
1 gi|358333041|dbj|DF143179.1| (12550bp)
For clarity introns have been scaled down by a factor of 1.17
Schistosoma japonicum
1500 bps (ex.)
8900 bps (in.)
1 gi|226598210|emb|CABF01094856.1| (183bp)
2 gi|226601353|emb|CABF01091713.1| (952bp)
3 gi|226659879|emb|CABF01033189.1| (669bp)
4 gi|226659878|emb|CABF01033190.1| (4264bp)
5 gi|226659877|emb|CABF01033191.1| (5819bp)
6 gi|226659876|emb|CABF01033192.1| (3011bp)
7 gi|226659875|emb|CABF01033193.1| (4814bp)
8 gi|226659874|emb|CABF01033194.1| (7405bp)
9 gi|226659873|emb|CABF01033195.1| (14664bp)
For clarity introns have been scaled down by a factor of 5.83
Schistosoma mansoni
1400 bps (ex.)
10200 bps (in.)
1 Schisto_mansoni.SC_0179 (47953bp)
For clarity introns have been scaled down by a factor of 7.36

Annelida

Helobdella robusta Mhc1

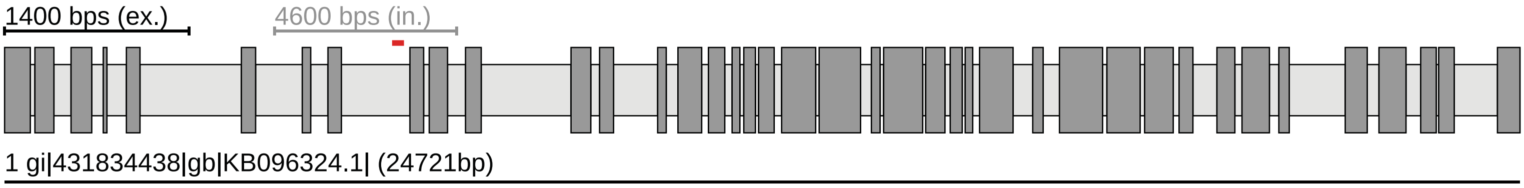

For clarity introns have been scaled down by a factor of 3.33

Helobdella robusta Mhc2

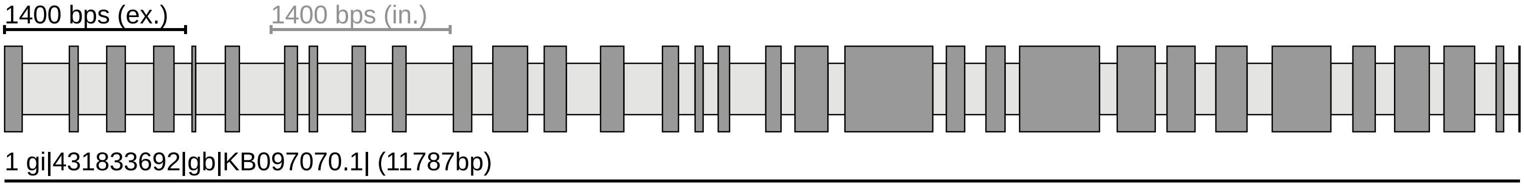

For clarity introns have been scaled down by a factor of 1.01

Capitella teleta

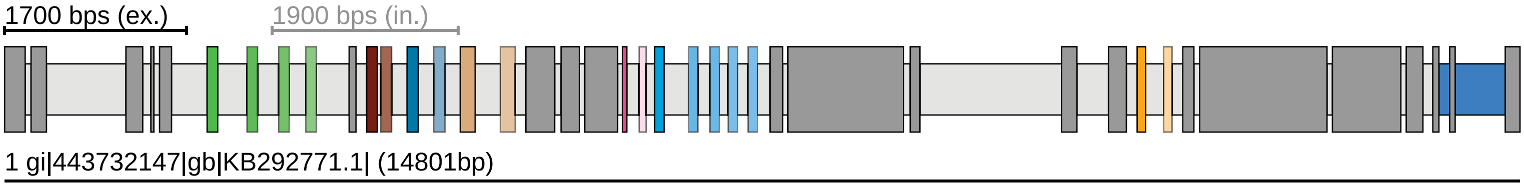

For clarity introns have been scaled down by a factor of 1.09

Mollusca

Crassostrea gigas Mhc1

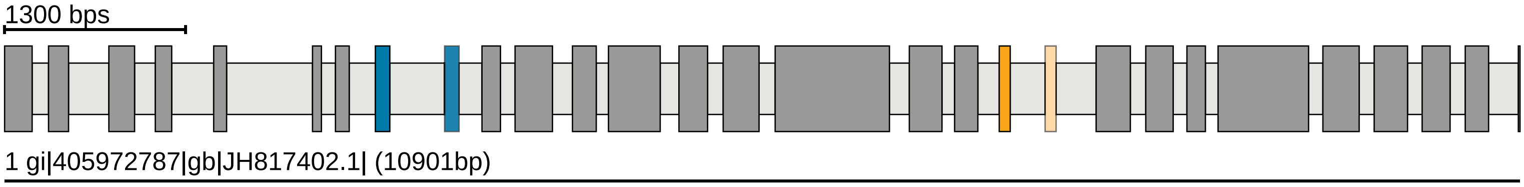

Crassostrea gigas Mhc2

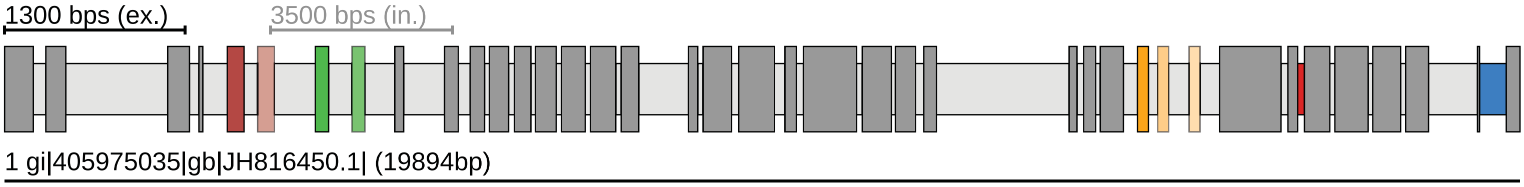

For clarity introns have been scaled down by a factor of 2.66

Aplysia californica

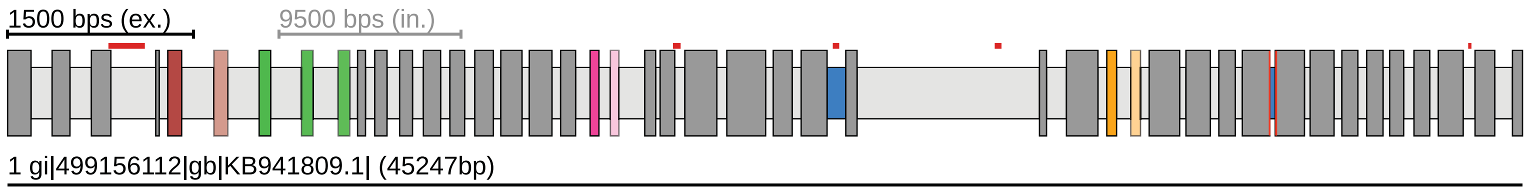

For clarity introns have been scaled down by a factor of 6.47

Biomphalaria glabrata

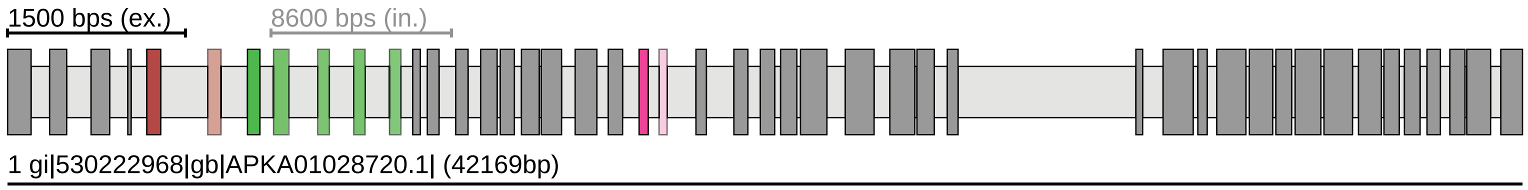

For clarity introns have been scaled down by a factor of 5.65

Lottia gigantea Mhc1

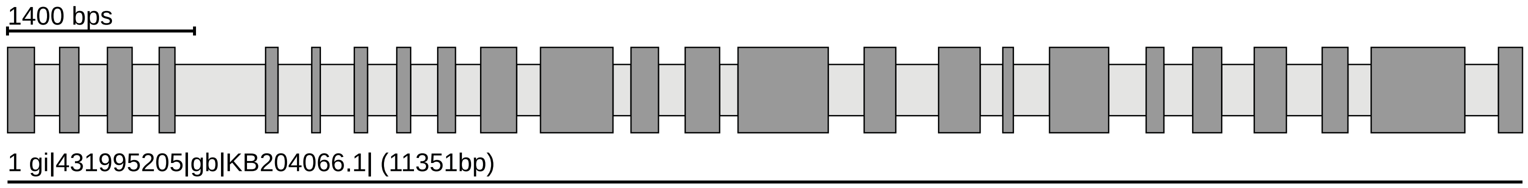

Lottia gigantea Mhc2

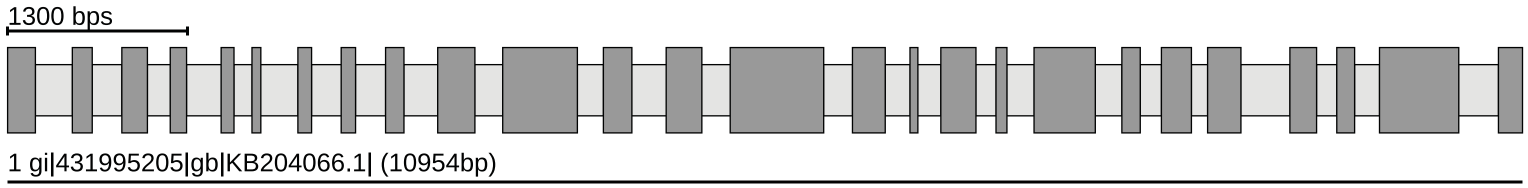

Lottia gigantea Mhc3

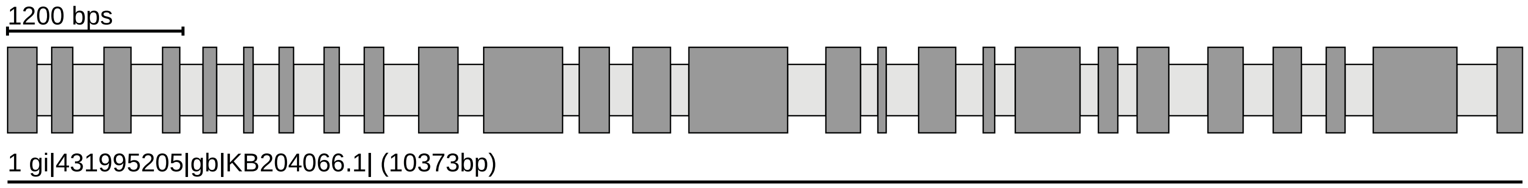

Lottia gigantea Mhc4

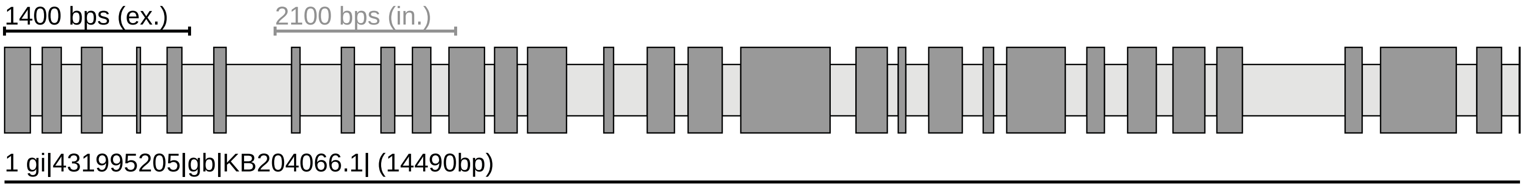

For clarity introns have been scaled down by a factor of 1.54

Lottia gigantea Mhc5

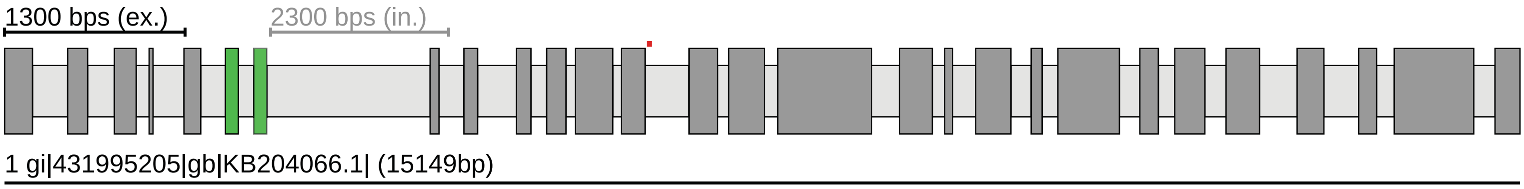

For clarity introns have been scaled down by a factor of 1.79

Lottia gigantea Mhc6

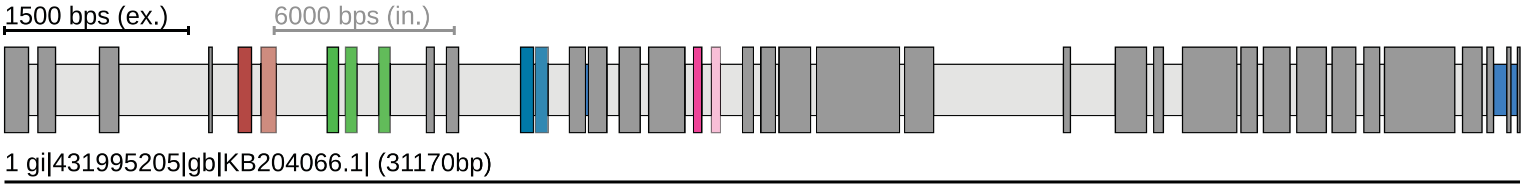

For clarity introns have been scaled down by a factor of 4.09

Chelicerata

Tetranychus urticae

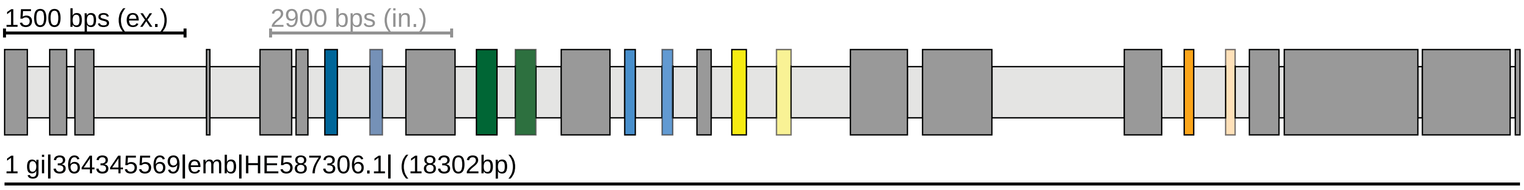

For clarity introns have been scaled down by a factor of 1.93

Ixodes scapularis

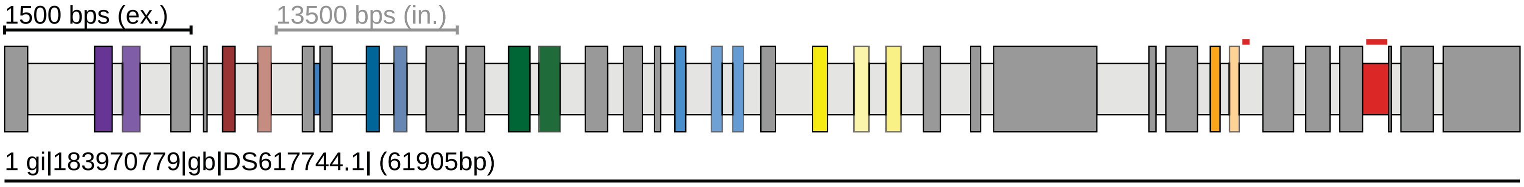

For clarity introns have been scaled down by a factor of 9.25

Metaseiulus occidentalis Mhc1

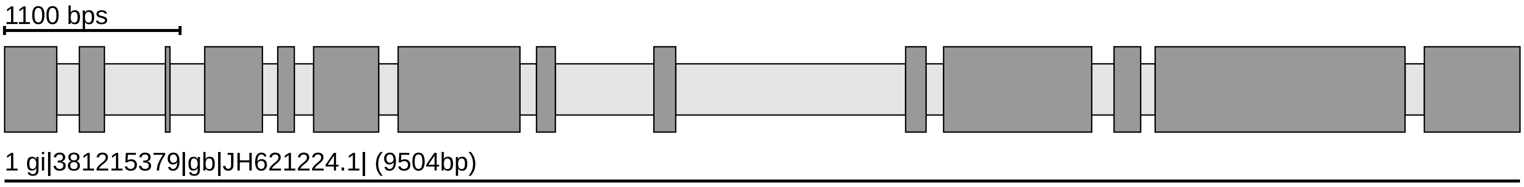

Metaseiulus occidentalis Mhc3

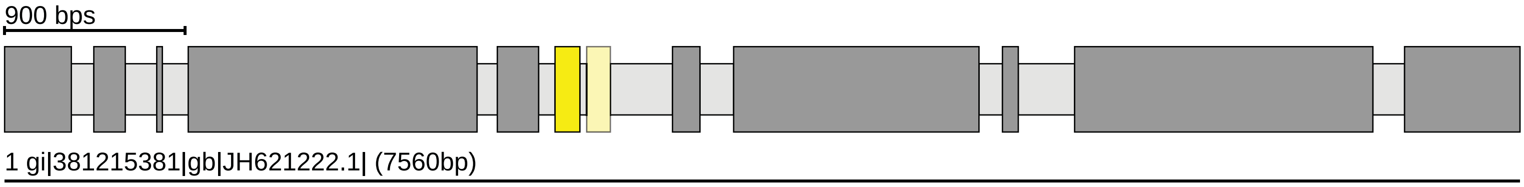

Metaseiulus occidentalis Mhc4

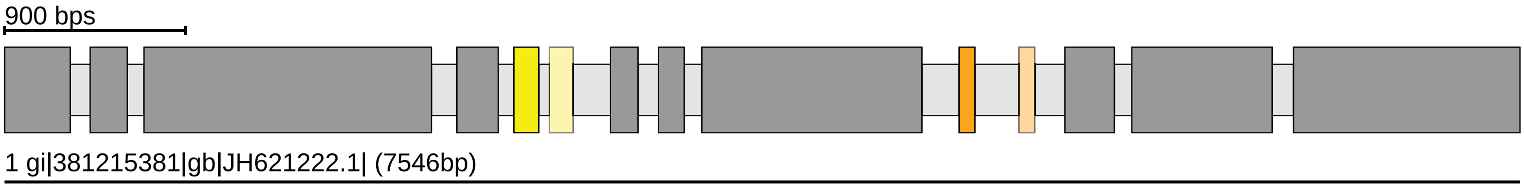

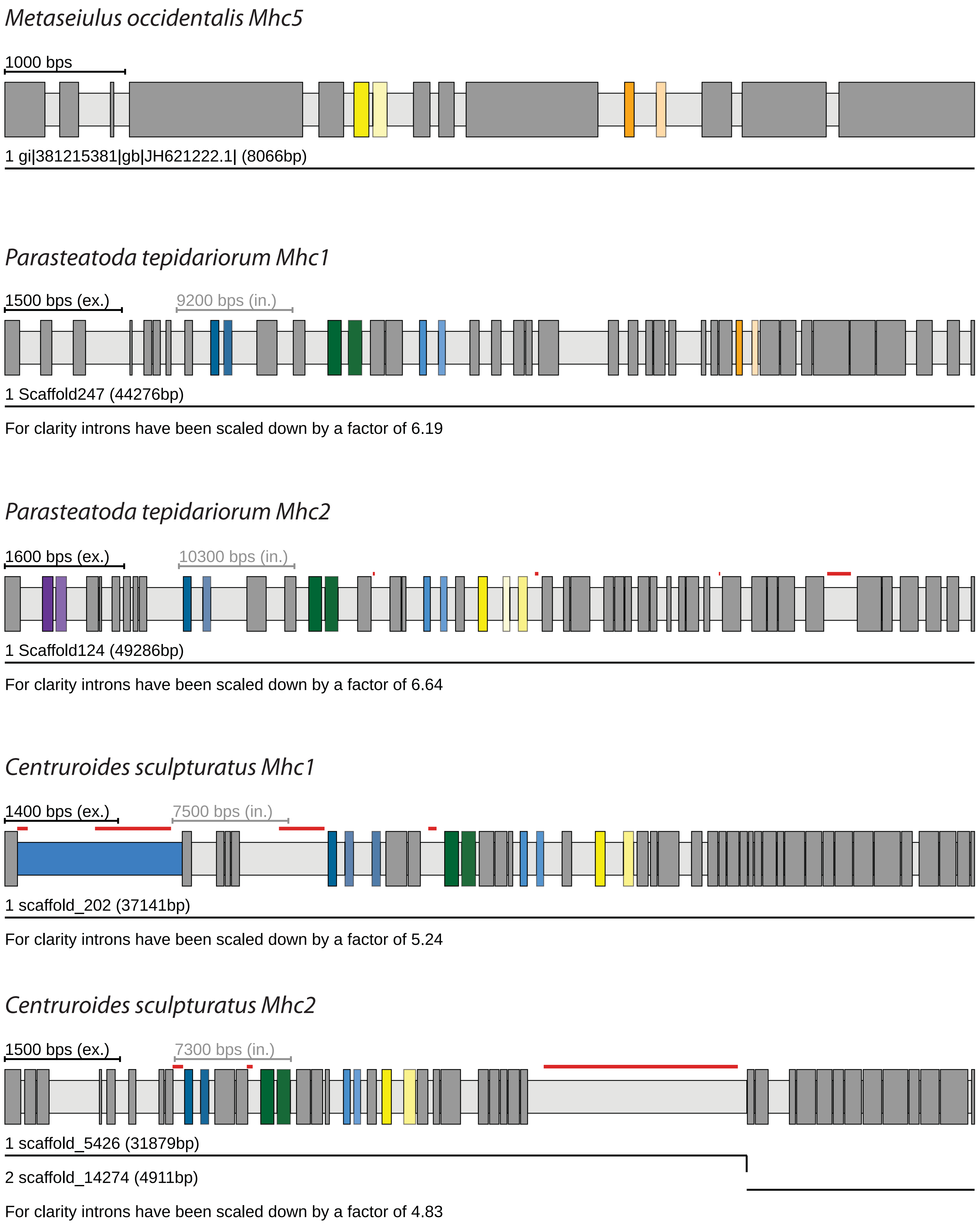
Metaseiulus occidentalis Mhc5
1000 bps
1 gi|381215381|gb|JH621222.1| (8066bp)
Parasteatoda tepidariorum Mhc1
1500 bps (ex.)
9200 bps (in.)
1 Scaffold247 (44276bp)
For clarity introns have been scaled down by a factor of 6.19
Parasteatoda tepidariorum Mhc2
1600 bps (ex.)
10300 bps (in.)
1 Scaffold124 (49286bp)
For clarity introns have been scaled down by a factor of 6.64
Centruroides sculpturatus Mhc1
1400 bps (ex.)
7500 bps (in.)
1 scaffold_202 (37141bp)
For clarity introns have been scaled down by a factor of 5.24
Centruroides sculpturatus Mhc2
1500 bps (ex.)
7300 bps (in.)
1 scaffold_5426 (31879bp)
2 scaffold_14274 (4911bp)
For clarity introns have been scaled down by a factor of 4.83

Chilopoda

Strigamia maritima

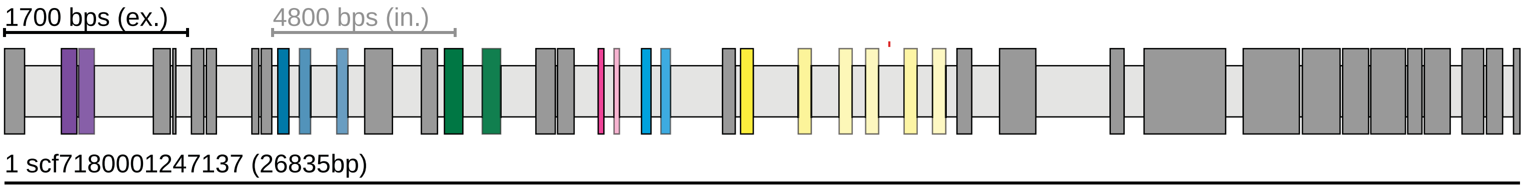

For clarity introns have been scaled down by a factor of 2.83

Crustacea

Daphnia pulex

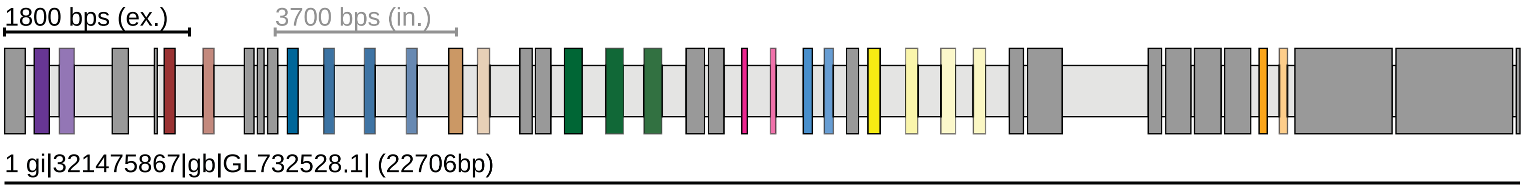

For clarity introns have been scaled down by a factor of 2.09

Lepeophtheirus salmonis Mhc1

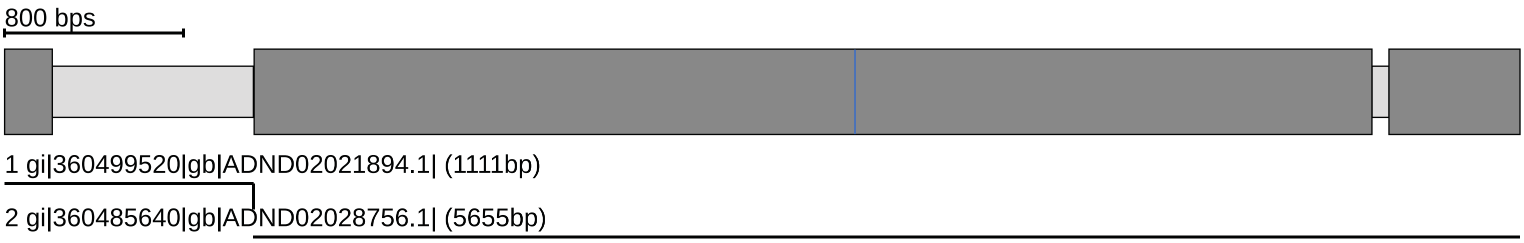

Lepeophtheirus salmonis Mhc2

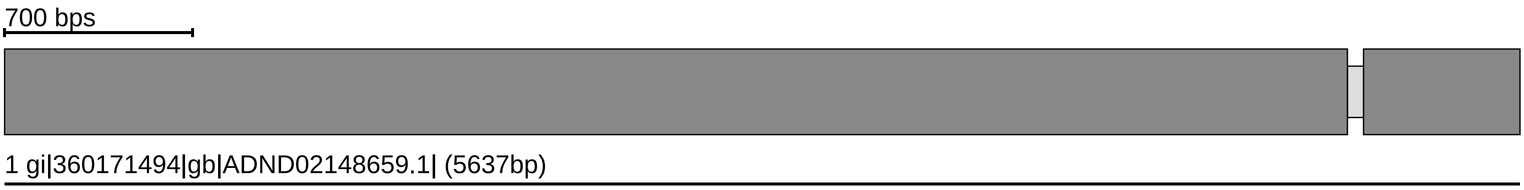

Lepeophtheirus salmonis Mhc3

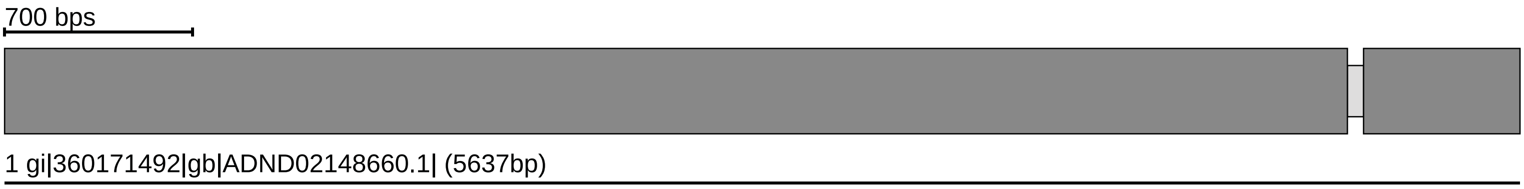

Lepeophtheirus salmonis Mhc4

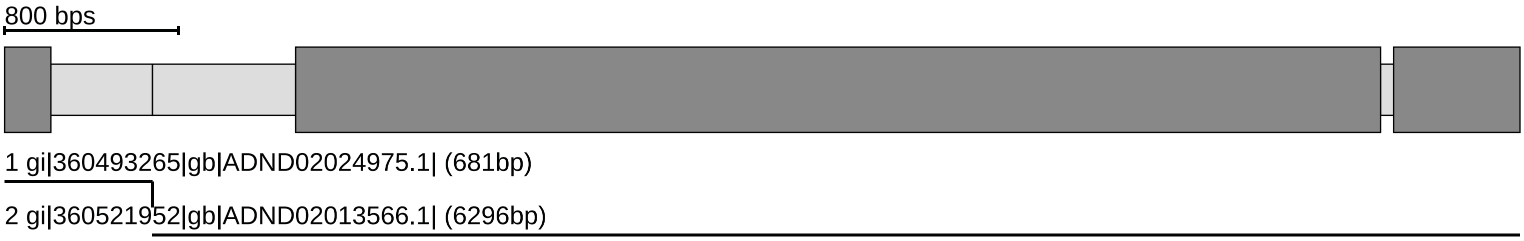

Lepeophtheirus salmonis Mhc5

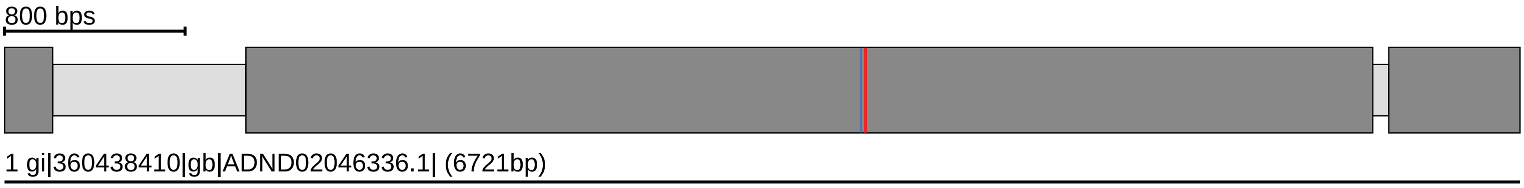

Lepeophtheirus salmonis Mhc6

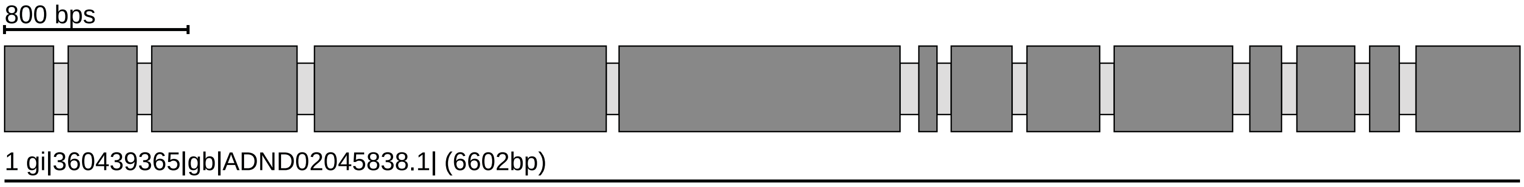

Lepeophtheirus salmonis Mhc7

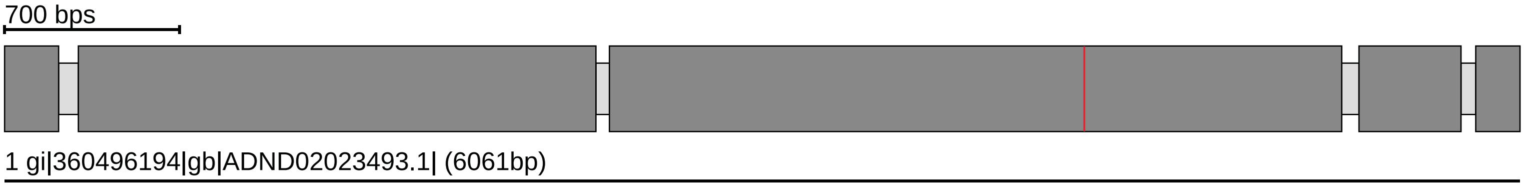

Lepeophtheirus salmonis Mhc8

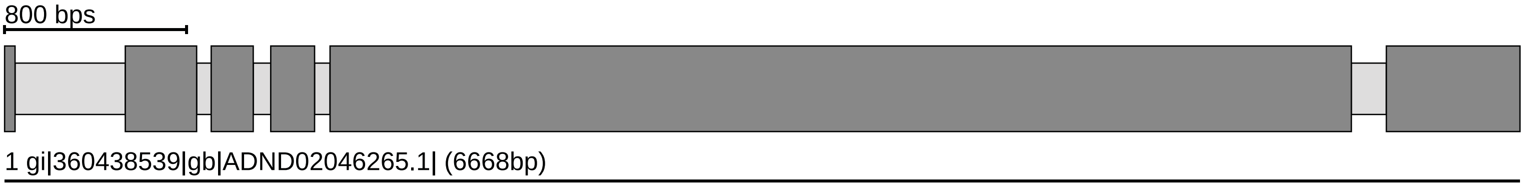

Lepeophtheirus salmonis Mhc9

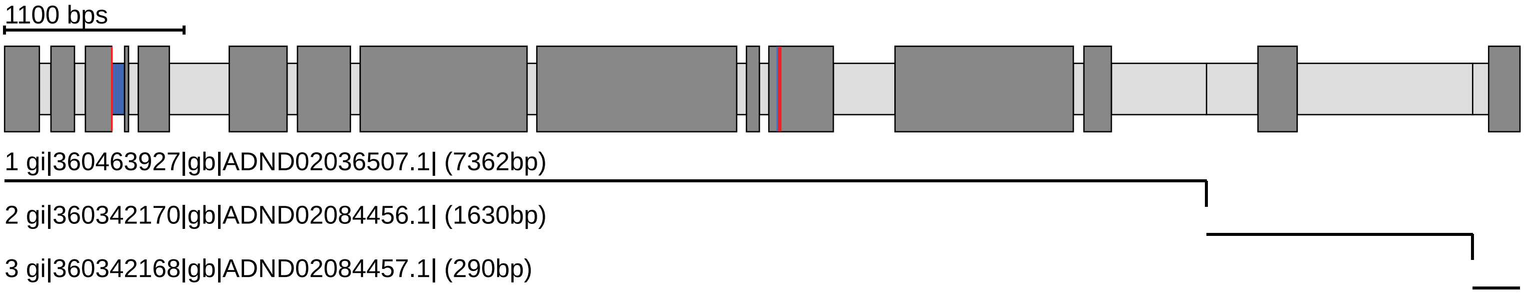

Lepeophtheirus salmonis Mhc10

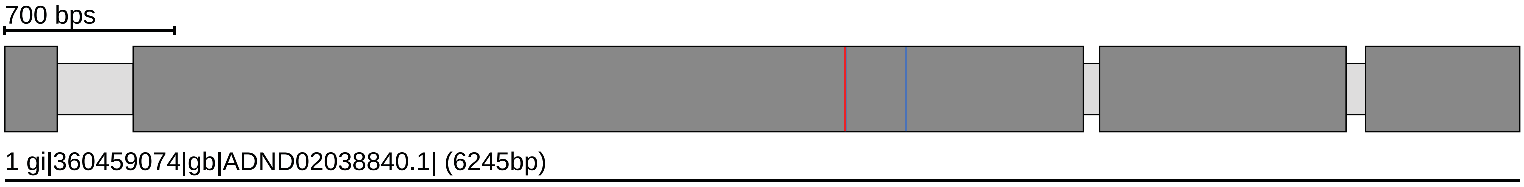

Lepeophtheirus salmonis Mhc11

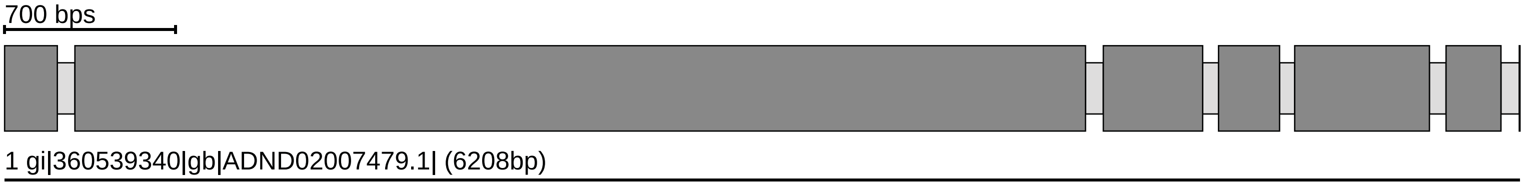

Lepeophtheirus salmonis Mhc12

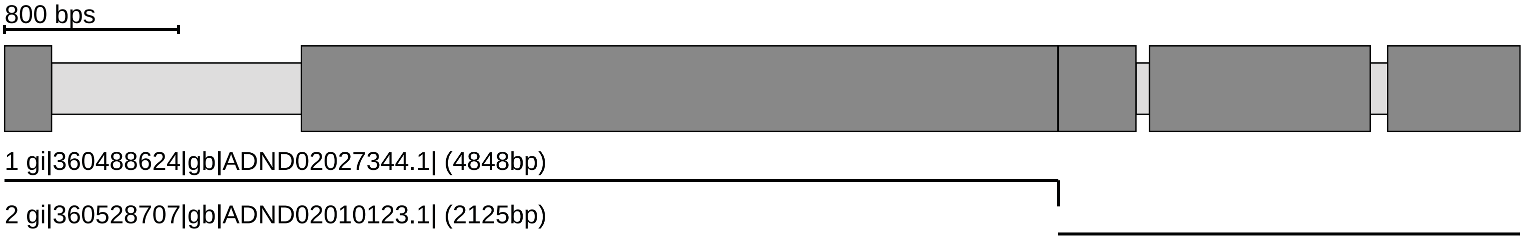

Lepeophtheirus salmonis Mhc13

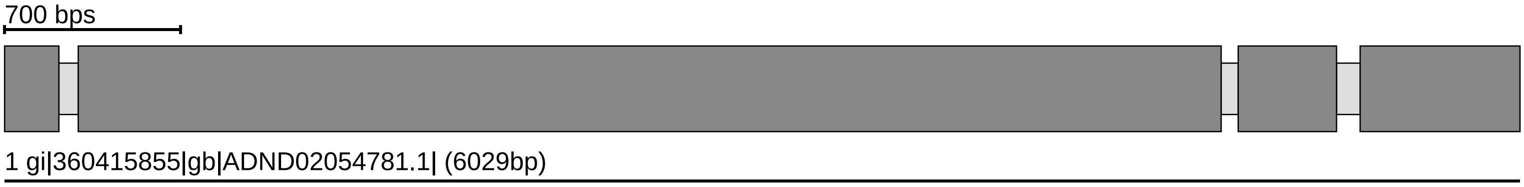

Lepeophtheirus salmonis Mhc14

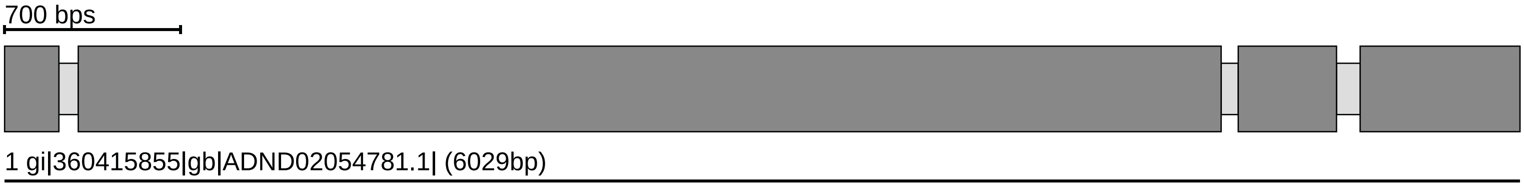

Lepeophtheirus salmonis Mhc15

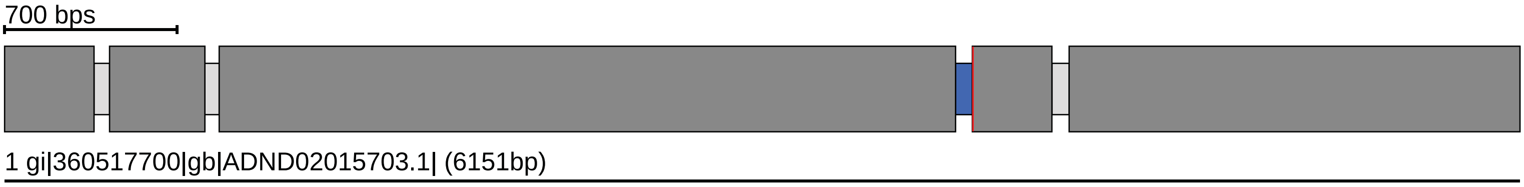

Lepeophtheirus salmonis Mhc16

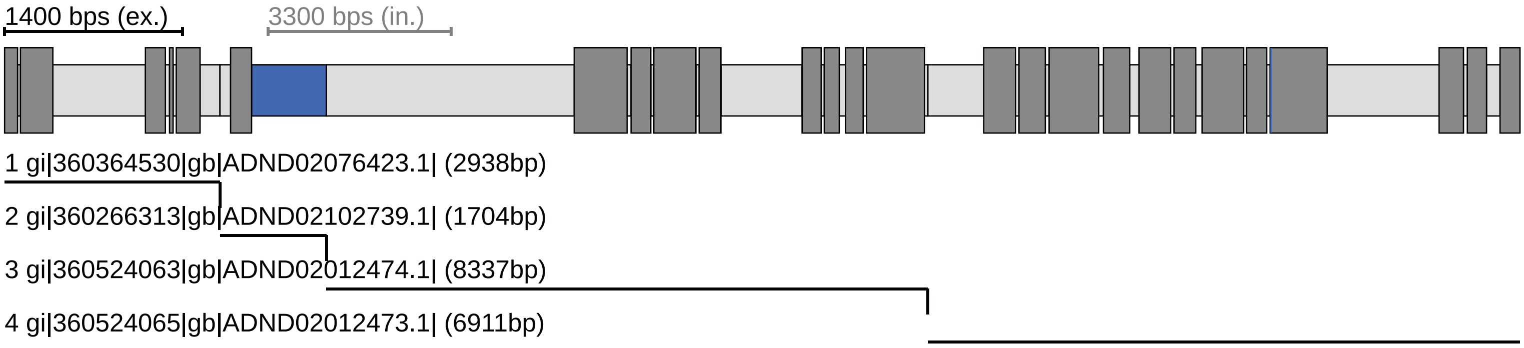

For clarity introns have been scaled down by a factor of 2.29

Lepeophtheirus salmonis Mhc17

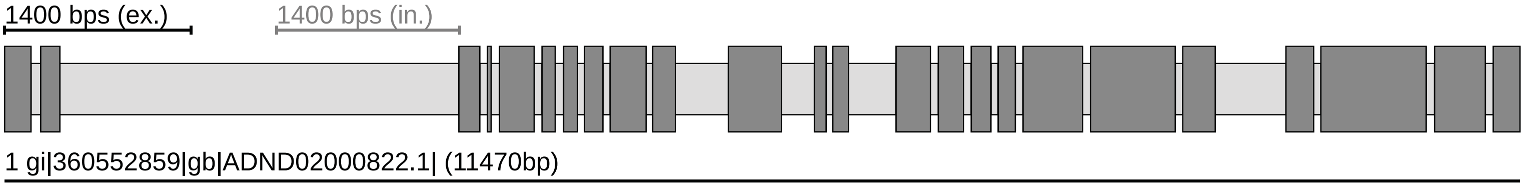

For clarity introns have been scaled down by a factor of 1.02

Palaeoptera

Ladona fulva

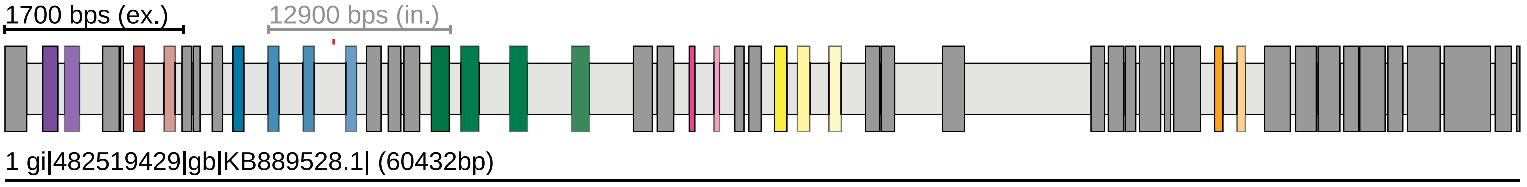

For clarity introns have been scaled down by a factor of 7.44

Ephemera danica

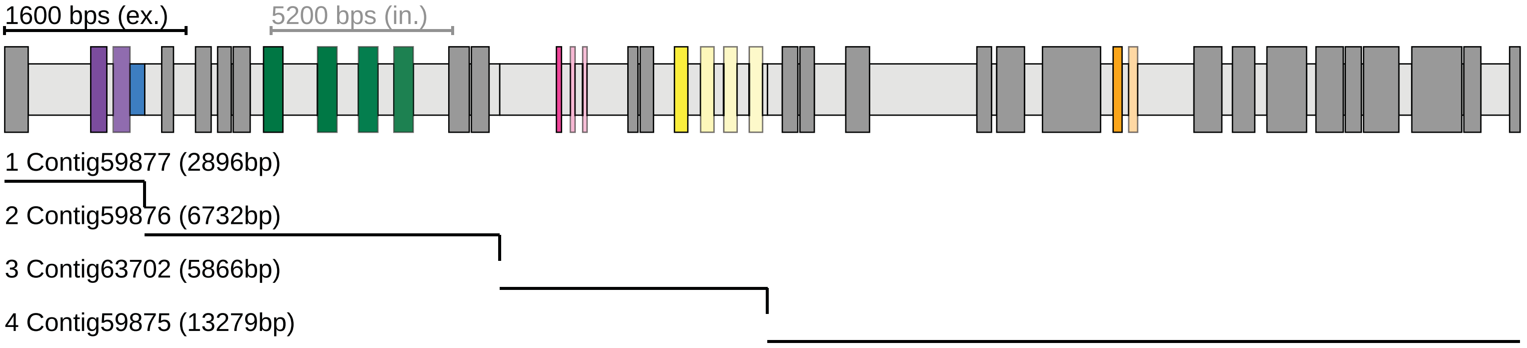

For clarity introns have been scaled down by a factor of 3.24

Paraneoptera

Homalodisca vitripennis

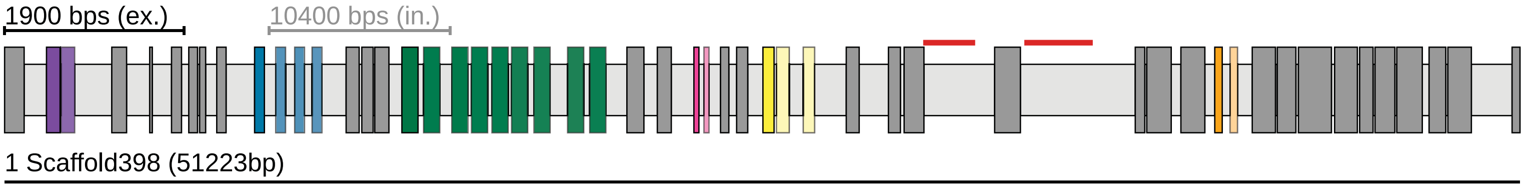

For clarity introns have been scaled down by a factor of 5.42

Cimex lectularius

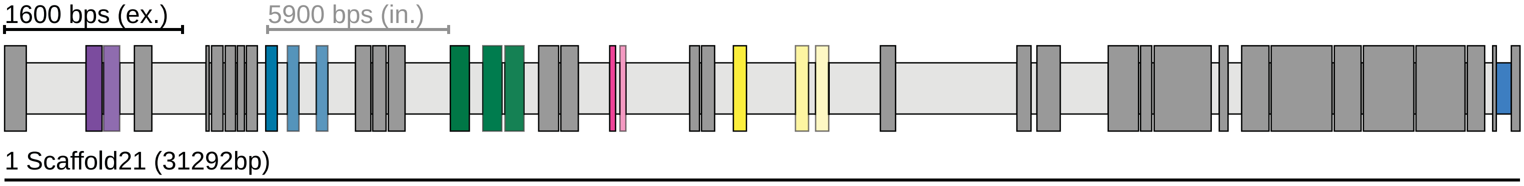

For clarity introns have been scaled down by a factor of 3.62

Rhodnius prolixus

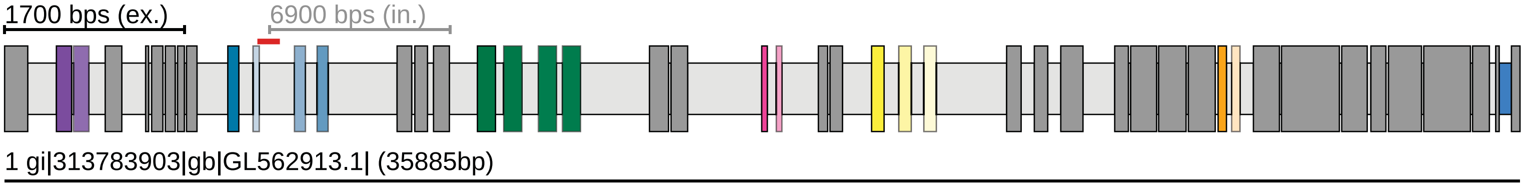

For clarity introns have been scaled down by a factor of 4.04

Acyrthosiphon pisum

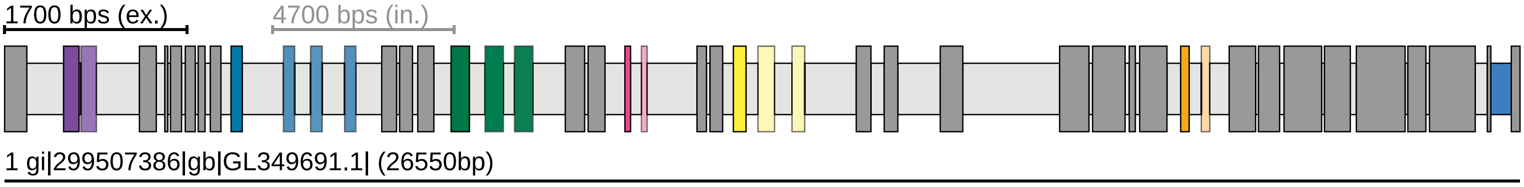

For clarity introns have been scaled down by a factor of 2.78

Pediculus humanus corporis

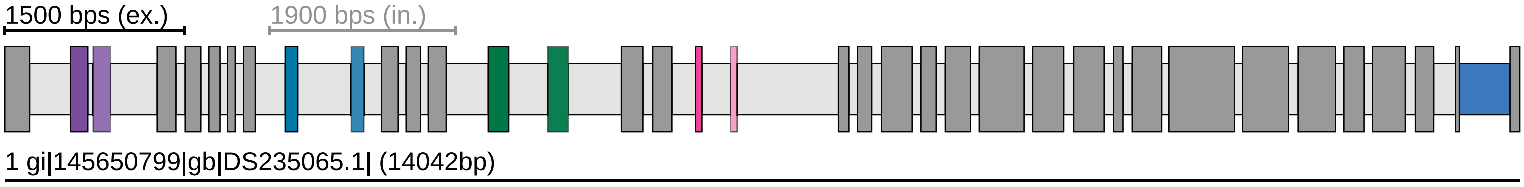

For clarity introns have been scaled down by a factor of 1.23

Coleoptera

Anoplophora glabripennis

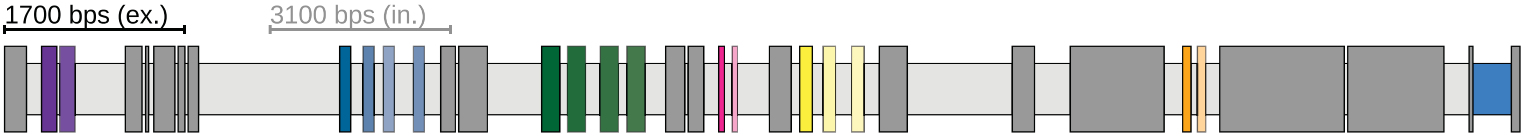

1 Scaffold60 (20071bp)

For clarity introns have been scaled down by a factor of 1.82

Leptinotarsa decemlineata

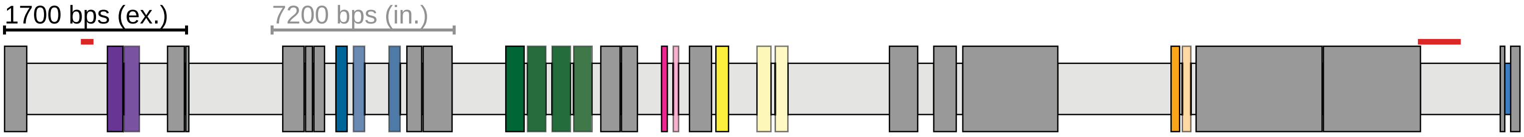

1 scaffold_905 (36717bp)

For clarity introns have been scaled down by a factor of 4.24

Dendroctonus ponderosae

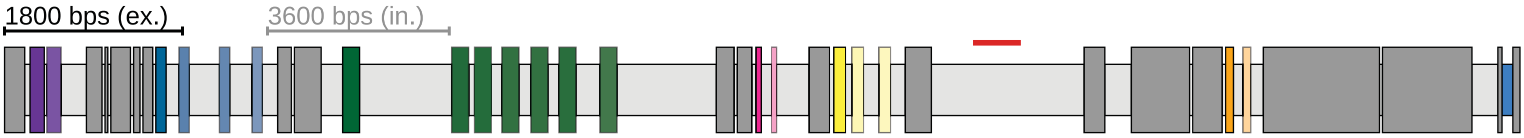

1 gi|459669322|gb|KB632028.1| (22607bp)

For clarity introns have been scaled down by a factor of 1.96

Tribolium castaneum

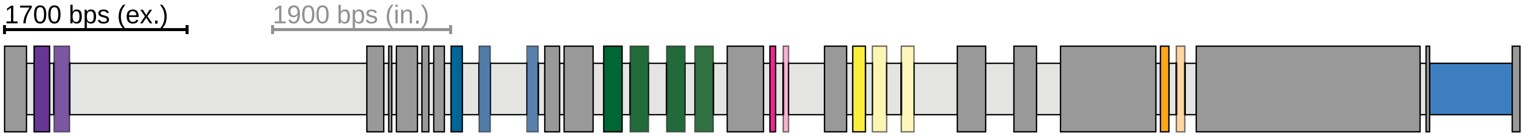

1 gi|158703262|gb|CM000283.2| (15112bp)

For clarity introns have been scaled down by a factor of 1.15

Agrilus planipennis

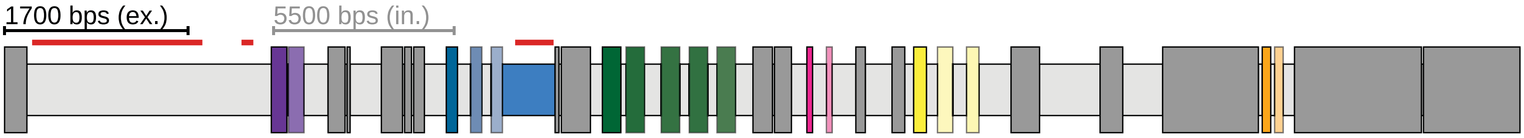

1 scaffold_11 (29858bp)

For clarity introns have been scaled down by a factor of 3.29

Strepsiptera

Mengenilla moldrzyki

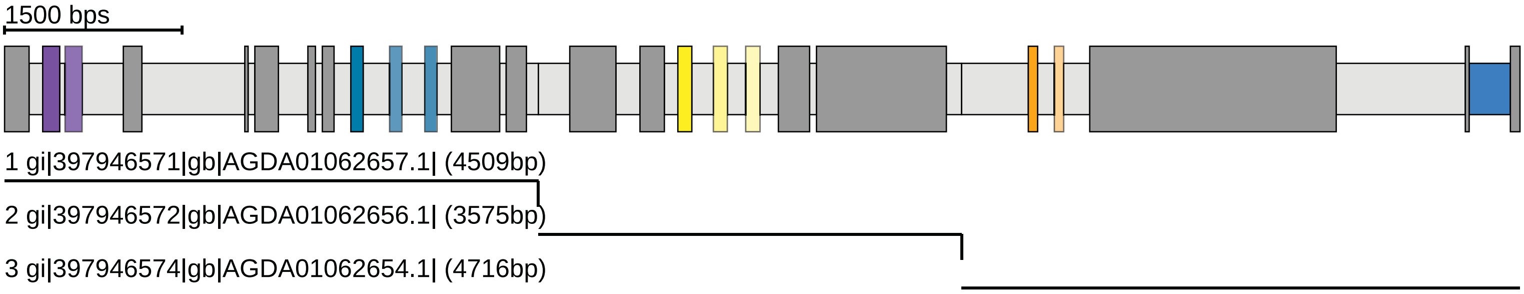

Hymenoptera

Apis florea

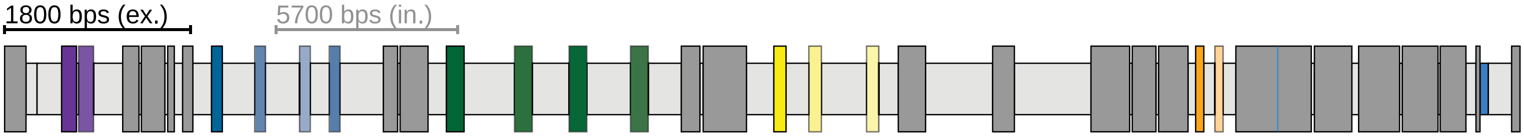

1 gi|313865953|gb|AEKZ01004571.1| (554bp)

2 gi|313861560|gb|AEKZ01008964.1| (29997bp)

3 gi|313861561|gb|AEKZ01008963.1| (813bp)

For clarity introns have been scaled down by a factor of 3.25

Apis mellifera

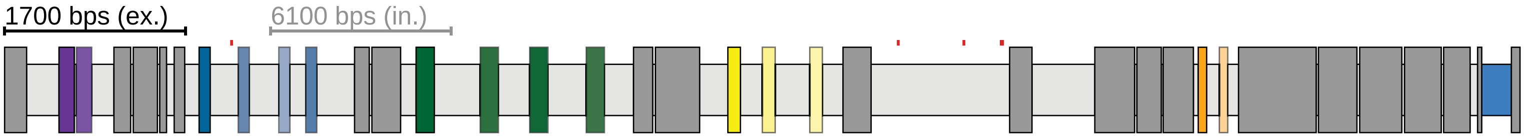

1 gi|318087629|gb|CM000061.5| (32516bp)

For clarity introns have been scaled down by a factor of 3.60

Bombus terrestris

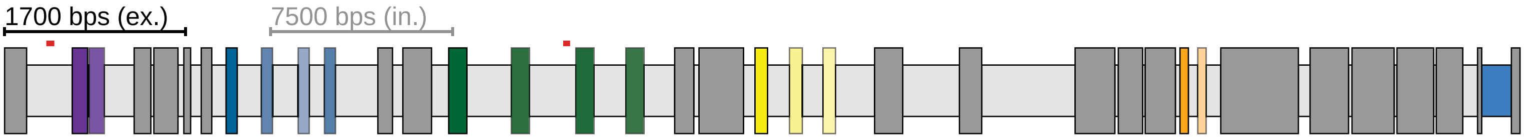

1 gi|334832617|gb|CM001177.1| (37985bp)

For clarity introns have been scaled down by a factor of 4.38

Bombus impatiens

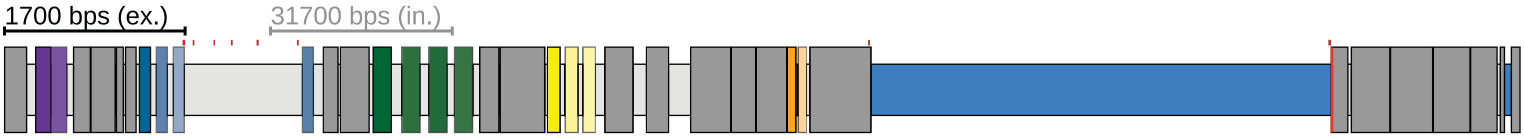

1 gi|322483879|gb|GL738842.1| (137618bp)

For clarity introns have been scaled down by a factor of 18.54

Lasioglossum albipes

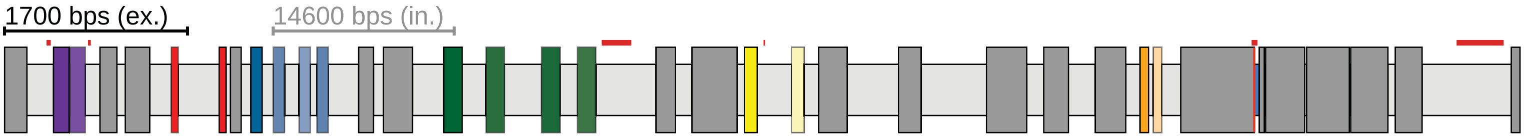

1 gi|469640073|gb|KB639309.1| (67354bp)

For clarity introns have been scaled down by a factor of 8.69

Megachile rotundata

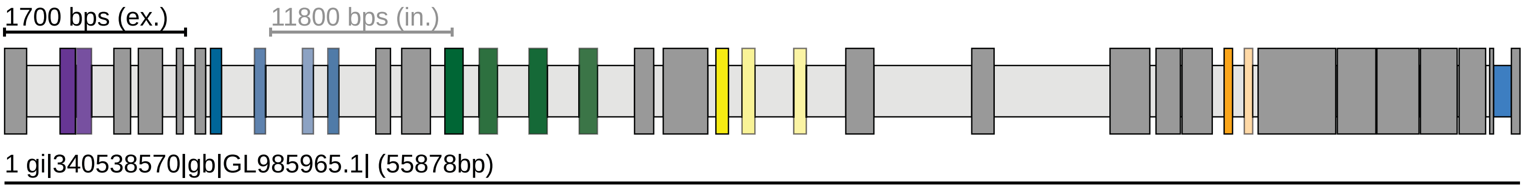

For clarity introns have been scaled down by a factor of 6.93

Linepithema humile

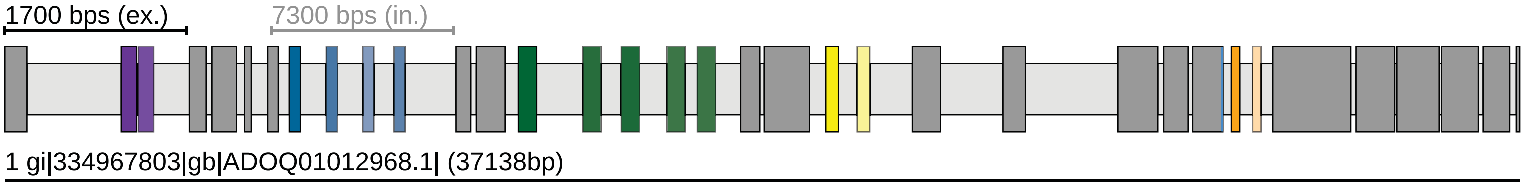

For clarity introns have been scaled down by a factor of 4.28

Camponotus floridanus

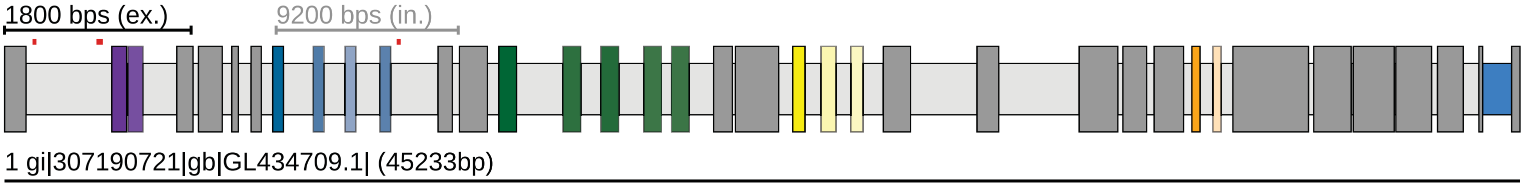

For clarity introns have been scaled down by a factor of 5.24

Acromyrmex echinatior

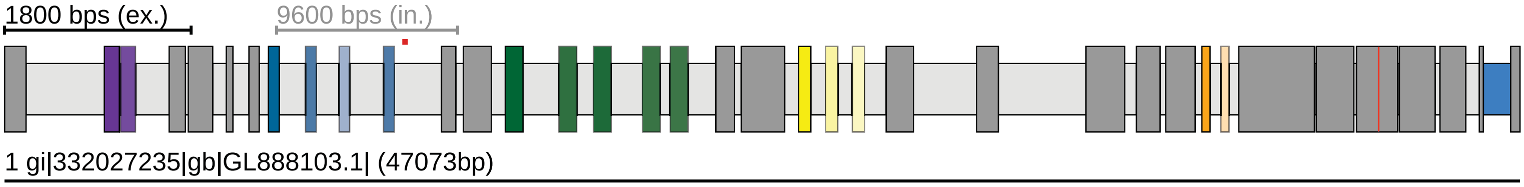

For clarity introns have been scaled down by a factor of 5.50

Atta cephalotes

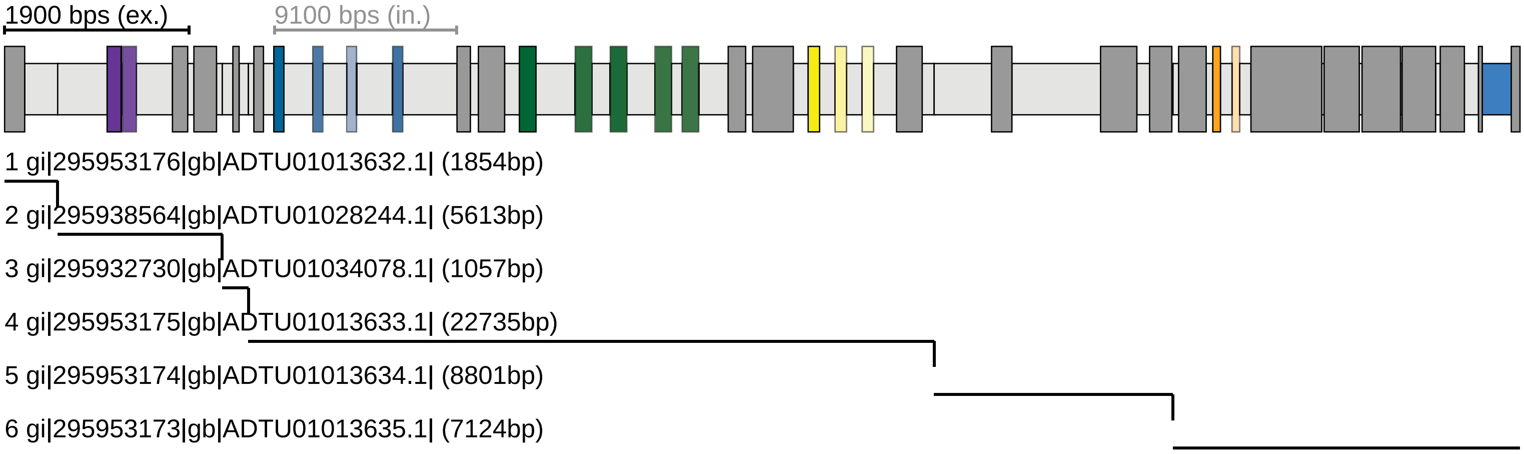

For clarity introns have been scaled down by a factor of 4.85

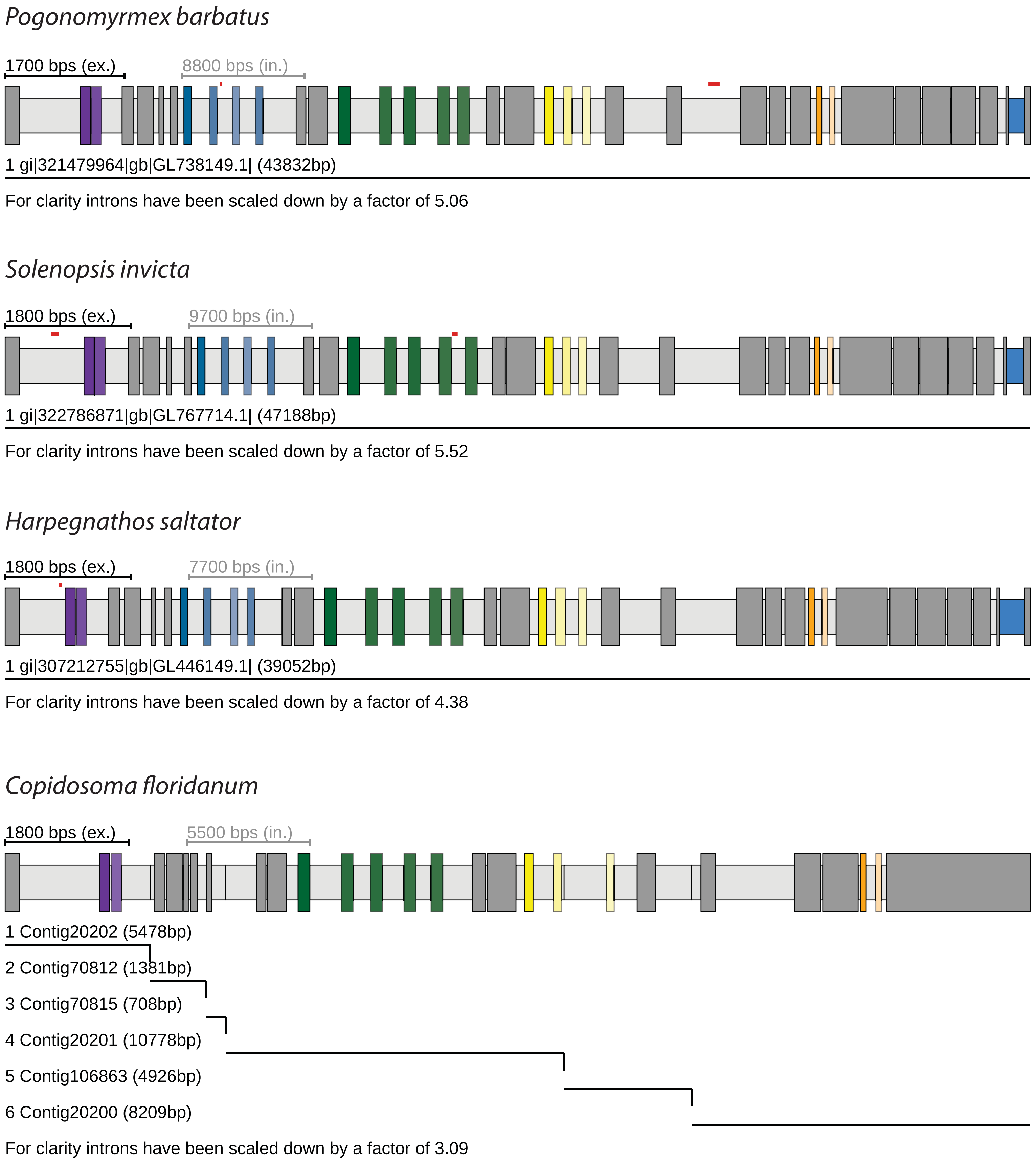

Pogonomyrmex barbatus
1700 bps (ex.)
8800 bps (in.)
1 gi|321479964|gb|GL738149.1| (43832bp)
For clarity introns have been scaled down by a factor of 5.06
Solenopsis invicta
1800 bps (ex.)
9700 bps (in.)
1 gi|322786871|gb|GL767714.1| (47188bp)
For clarity introns have been scaled down by a factor of 5.52
Harpegnathos saltator
1800 bps (ex.)
7700 bps (in.)
1 gi|307212755|gb|GL446149.1| (39052bp)
For clarity introns have been scaled down by a factor of 4.38
Copidosoma floridanum
1800 bps (ex.)
5500 bps (in.)
1 Contig20202 (5478bp)
2 Contig70812 (1381bp)
3 Contig70815 (708bp)
4 Contig20201 (10778bp)
5 Contig106863 (4926bp)
6 Contig20200 (8209bp)
For clarity introns have been scaled down by a factor of 3.09

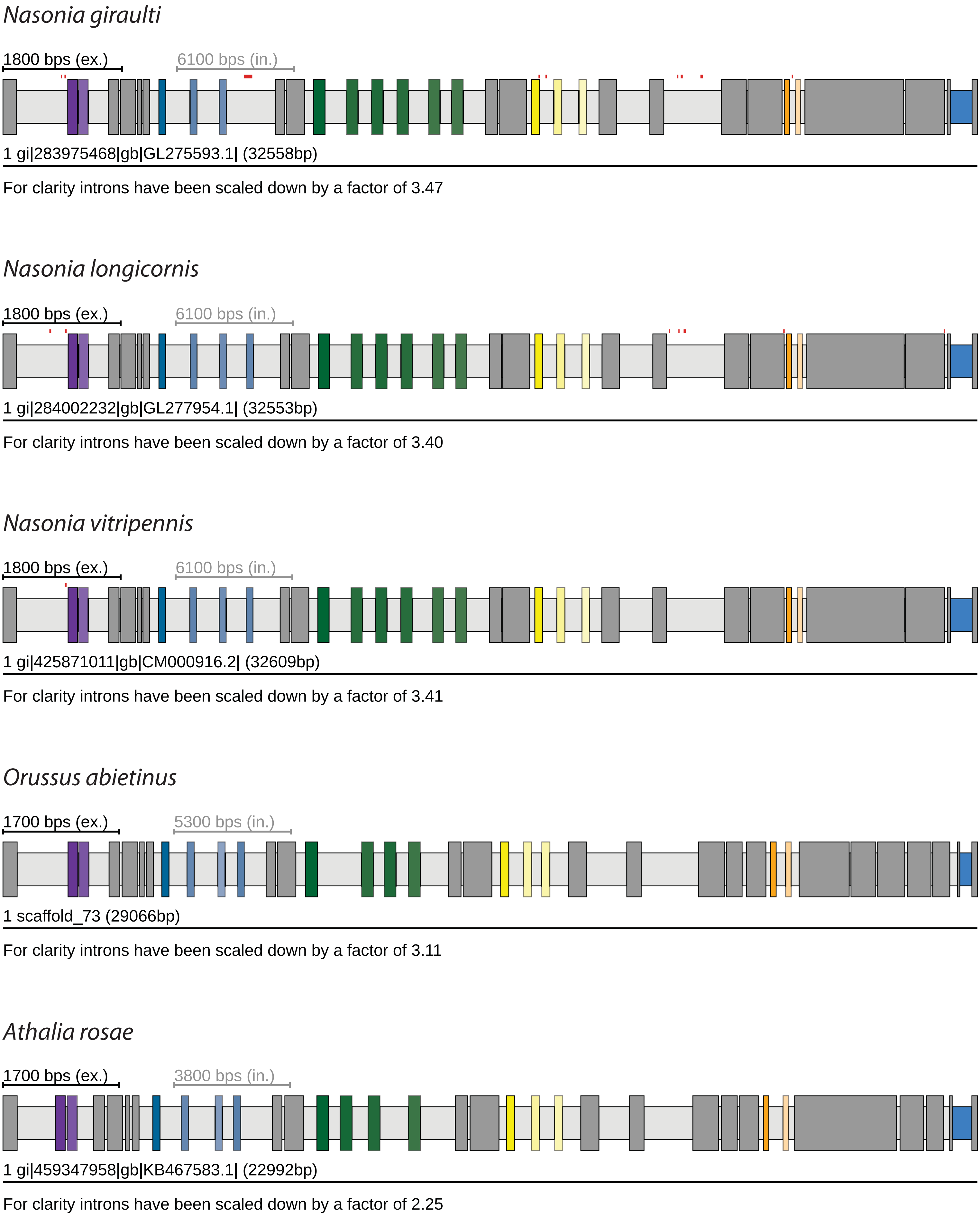
Nasonia giraulti
1800 bps (ex.)
6100 bps (in.)
1 gi|283975468|gb|GL275593.1| (32558bp)
For clarity introns have been scaled down by a factor of 3.47
Nasonia longicornis
1800 bps (ex.)
6100 bps (in.)
1 gi|284002232|gb|GL277954.1| (32553bp)
For clarity introns have been scaled down by a factor of 3.40
Nasonia vitripennis
1800 bps (ex.)
6100 bps (in.)
1 gi|425871011|gb|CM000916.2| (32609bp)
For clarity introns have been scaled down by a factor of 3.41
Orussus abietinus
1700 bps (ex.)
5300 bps (in.)
1 scaffold_73 (29066bp)
For clarity introns have been scaled down by a factor of 3.11
Athalia rosae
1700 bps (ex.)
3800 bps (in.)
1 gi|459347958|gb|KB467583.1| (22992bp)
For clarity introns have been scaled down by a factor of 2.25

Trichoptera / Limnephilidae

Limnephilus lunatus

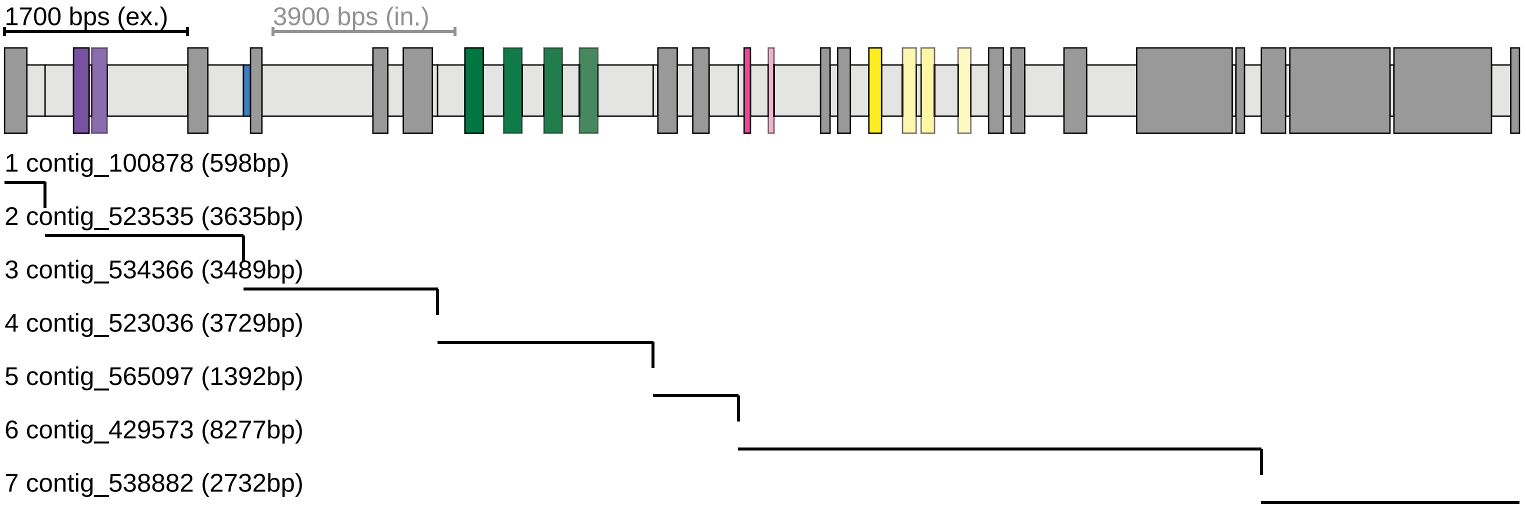

For clarity introns have been scaled down by a factor of 2.31

Lepidoptera

Bombyx mori

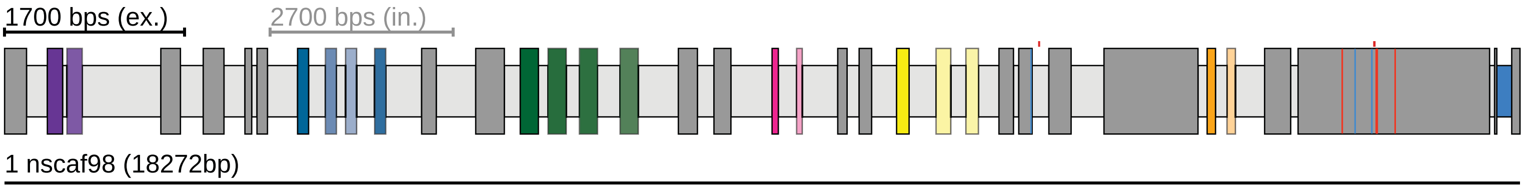

For clarity introns have been scaled down by a factor of 1.56

Bombyx mandarina

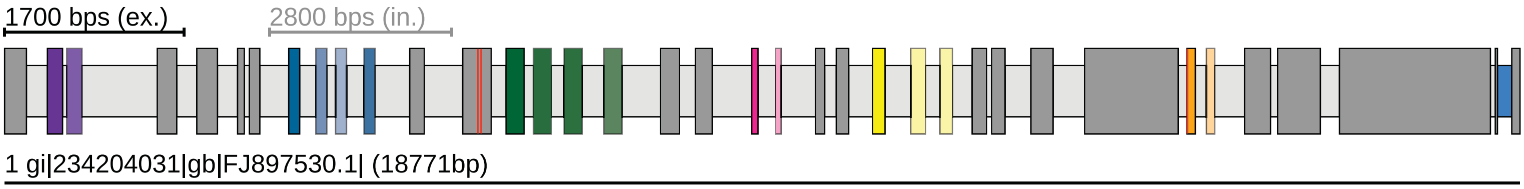

For clarity introns have been scaled down by a factor of 1.62

Manduca sexta

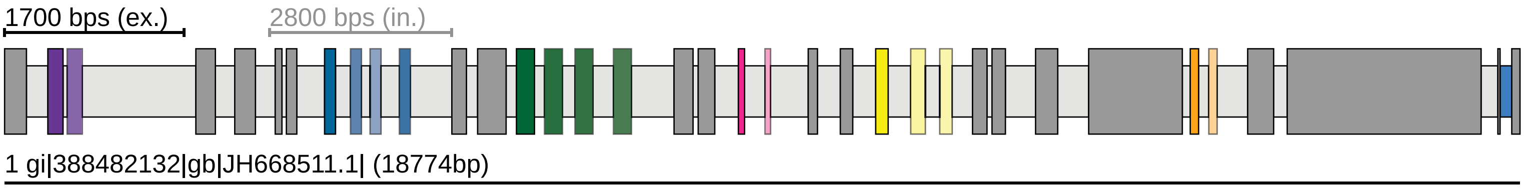

For clarity introns have been scaled down by a factor of 1.63

Helicoverpa punctigera

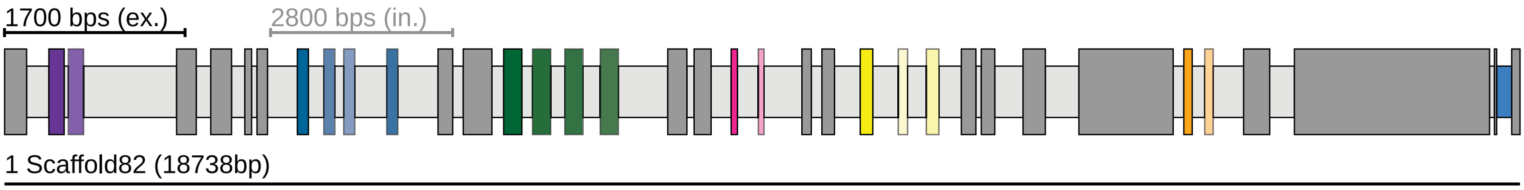

For clarity introns have been scaled down by a factor of 1.64

Danaus plexippus

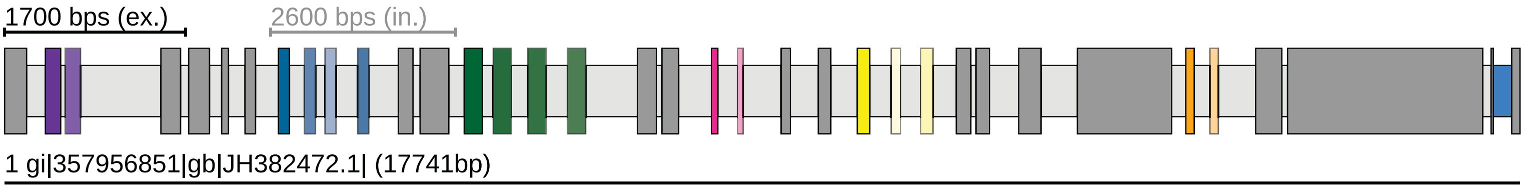

For clarity introns have been scaled down by a factor of 1.50

Heliconius melpomene melpomene

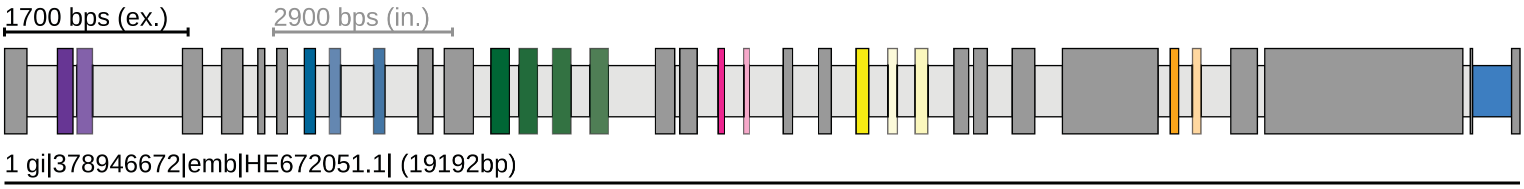

For clarity introns have been scaled down by a factor of 1.74

Plutella xylostella

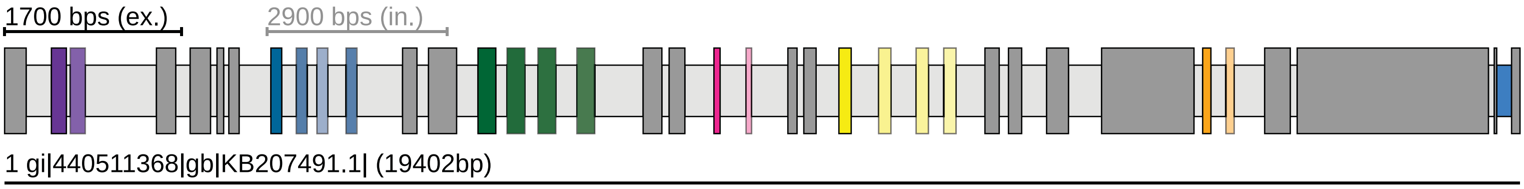

For clarity introns have been scaled down by a factor of 1.68

Cecidomyiidae

Mayetiola destructor

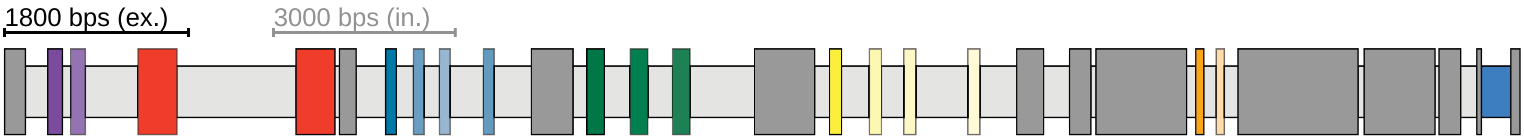

1 gi|309241391|gb|GL501433.1| (19873bp)

For clarity introns have been scaled down by a factor of 1.69

Culicidae

Anopheles funestus

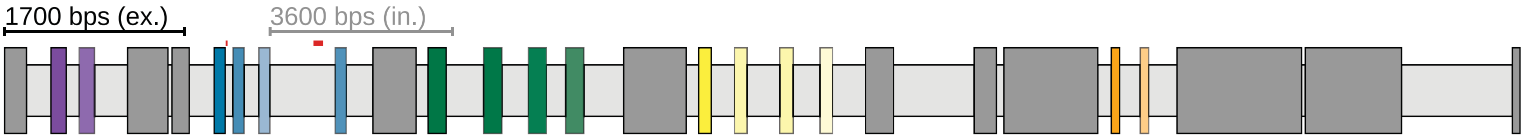

1 gi|471791571|gb|KB669070.1| (21930bp)

For clarity introns have been scaled down by a factor of 2.08

Anopheles minimus

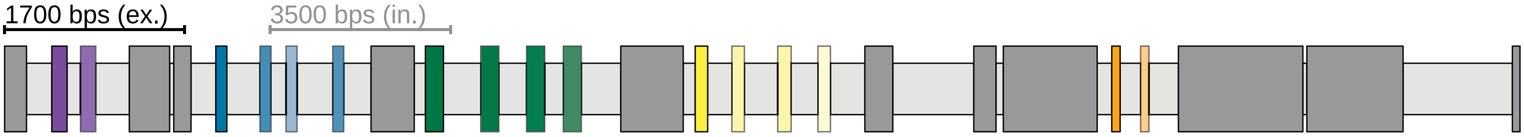

1 gi|471781521|gb|KB663821.1| (21697bp)

For clarity introns have been scaled down by a factor of 2.05

Anopheles stephensi

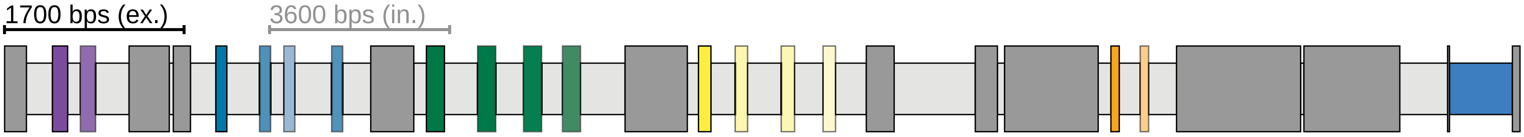

1 gi|407629776|gb|ALPR01041940.1| (22151bp)

For clarity introns have been scaled down by a factor of 2.10

Anopheles dirus

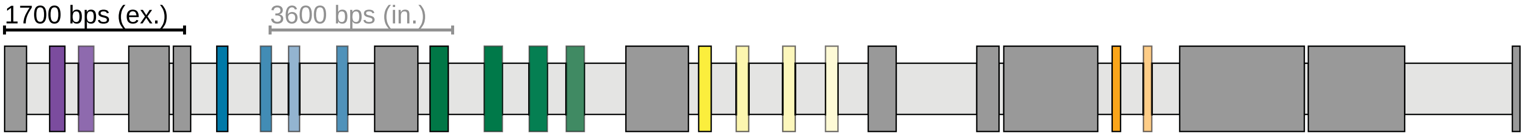

1 gi|471800525|gb|KB673290.1| (21964bp)

For clarity introns have been scaled down by a factor of 2.09

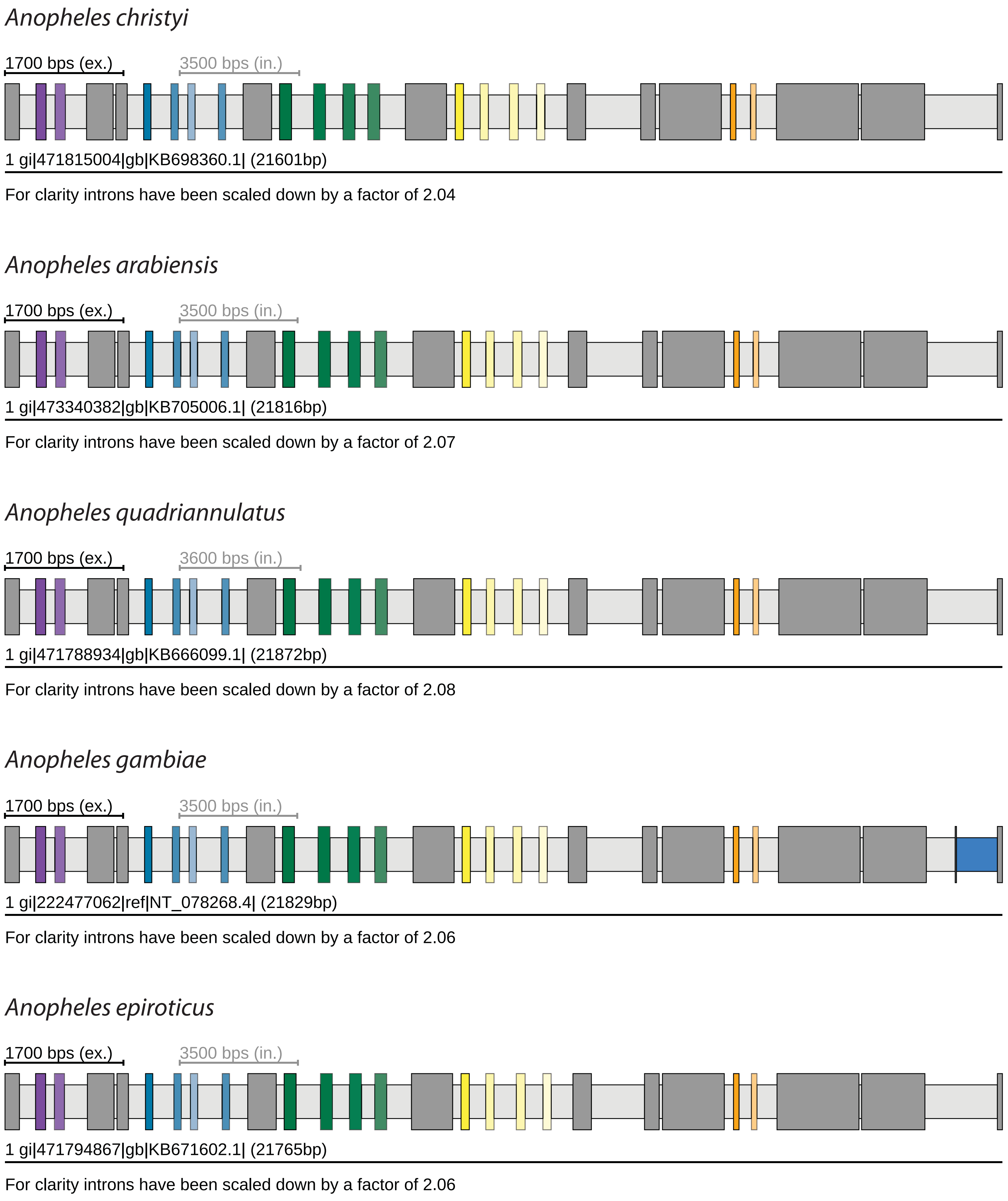
Anopheles christyi
1700 bps (ex.)
3500 bps (in.)
1 gi|471815004|gb|KB698360.1| (21601bp)
For clarity introns have been scaled down by a factor of 2.04
Anopheles arabiensis
1700 bps (ex.)
3500 bps (in.)
1 gi|473340382|gb|KB705006.1| (21816bp)
For clarity introns have been scaled down by a factor of 2.07
Anopheles quadriannulatus
1700 bps (ex.)
3600 bps (in.)
1 gi|471788934|gb|KB666099.1| (21872bp)
For clarity introns have been scaled down by a factor of 2.08
Anopheles gambiae
1700 bps (ex.)
3500 bps (in.)
1 gi|222477062|ref|NT_078268.4| (21829bp)
For clarity introns have been scaled down by a factor of 2.06
Anopheles epiroticus
1700 bps (ex.)
3500 bps (in.)
1 gi|471794867|gb|KB671602.1| (21765bp)
For clarity introns have been scaled down by a factor of 2.06

Anopheles albimanus

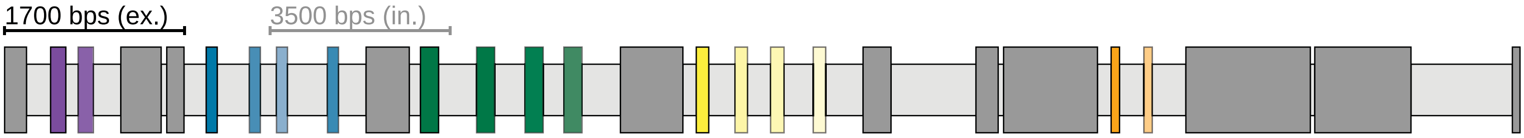

1 gi|471799144|gb|KB672435.1| (21737bp)

For clarity introns have been scaled down by a factor of 2.06

Anopheles darlingi

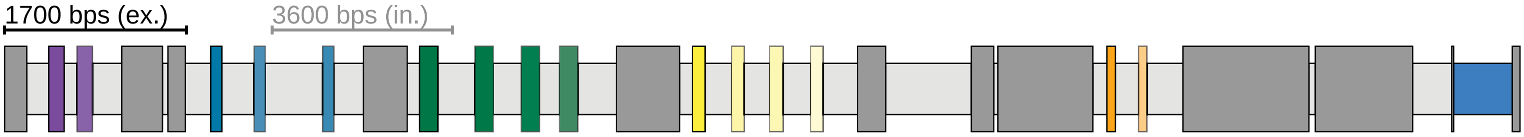

1 gi|312370895|gb|ADMH01003948.1| (22031bp)

For clarity introns have been scaled down by a factor of 2.14

Aedes aegypti Mhc1

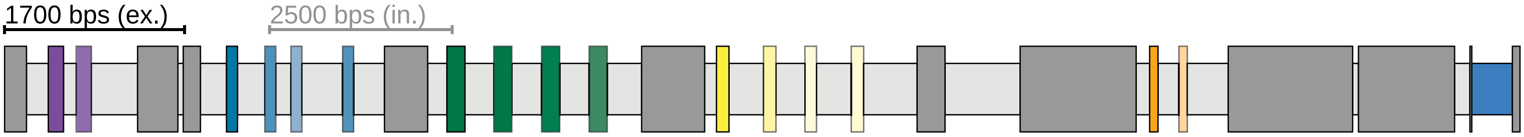

1 gi|78156411|gb|AAGE02009209.1| (17486bp)

For clarity introns have been scaled down by a factor of 1.45

Aedes aegypti Mhc3

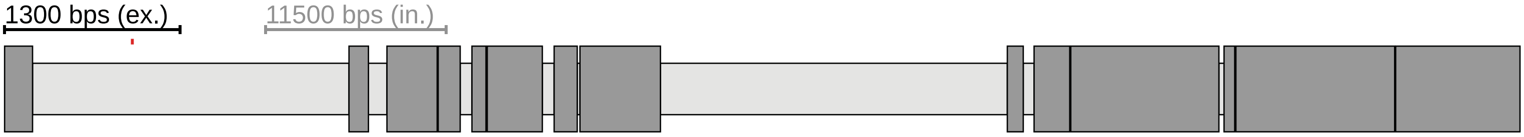

1 gi|78215711|gb|CH477353.1| (52255bp)

For clarity introns have been scaled down by a factor of 8.59

Culex pipiens quinquefasciatus Mhc1

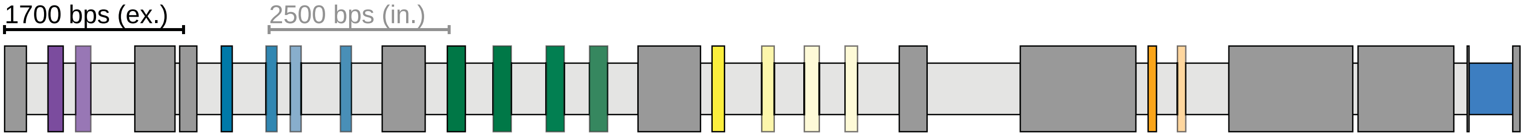

1 gi|145648990|gb|DS231821.1| (17639bp)

For clarity introns have been scaled down by a factor of 1.46

Culex pipiens quinquefasciatus Mhc3

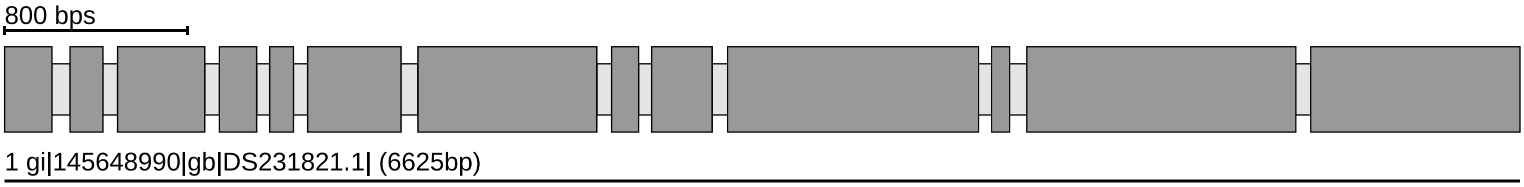

Culex pipiens quinquefasciatus Mhc4

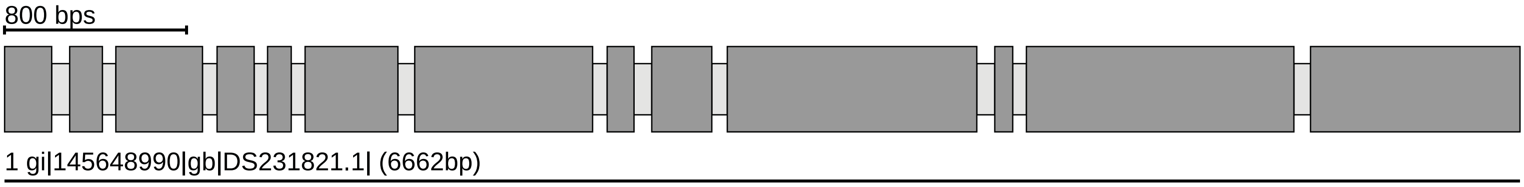

Psychodidae

Lutzomyia longipalpis

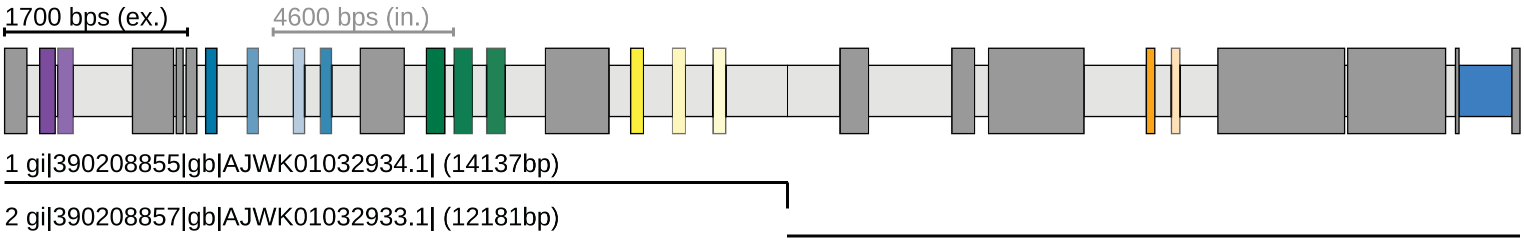

For clarity introns have been scaled down by a factor of 2.74

Phlebotomus papatasi

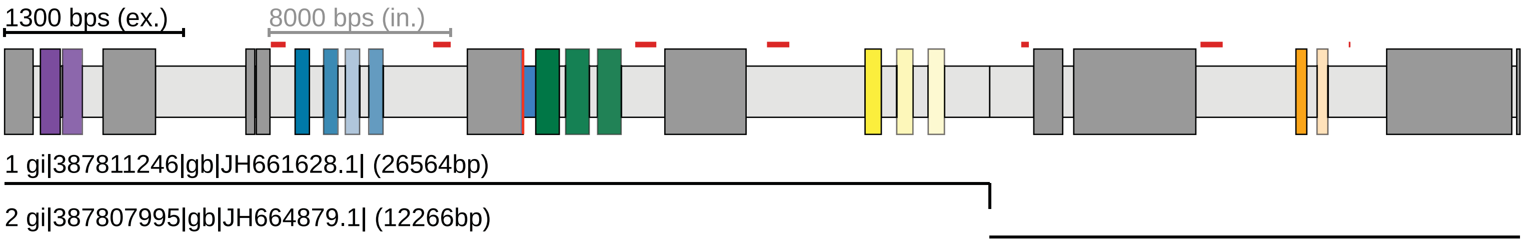

For clarity introns have been scaled down by a factor of 6.05

Brachycera

Ceratitis capitata

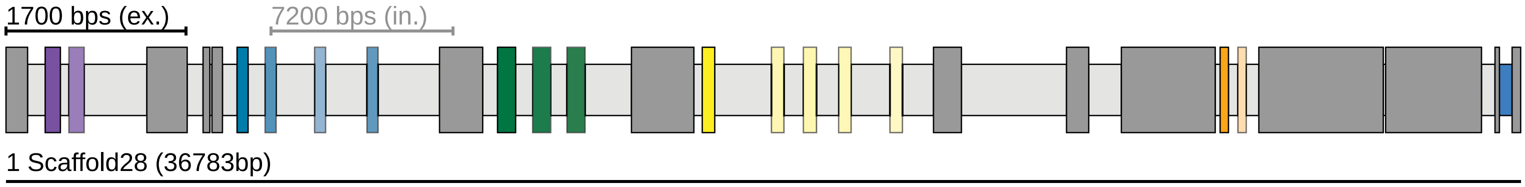

For clarity introns have been scaled down by a factor of 4.20

Glossina morsitans morsitans

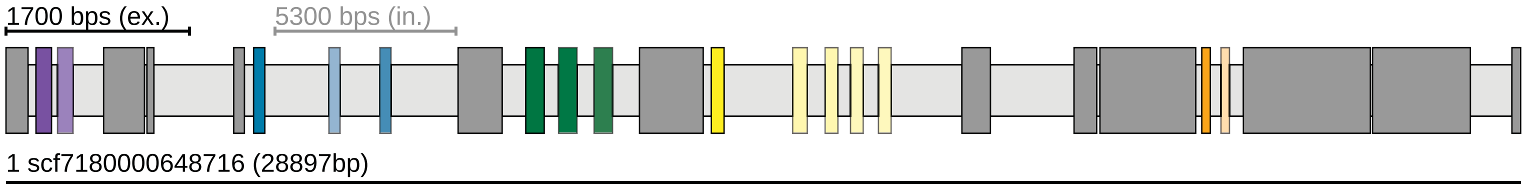

For clarity introns have been scaled down by a factor of 3.16

Musca domestica

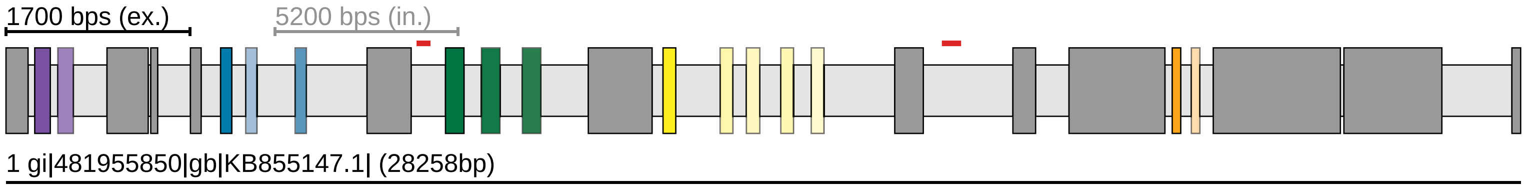

For clarity introns have been scaled down by a factor of 3.07

Drosophila albomicans

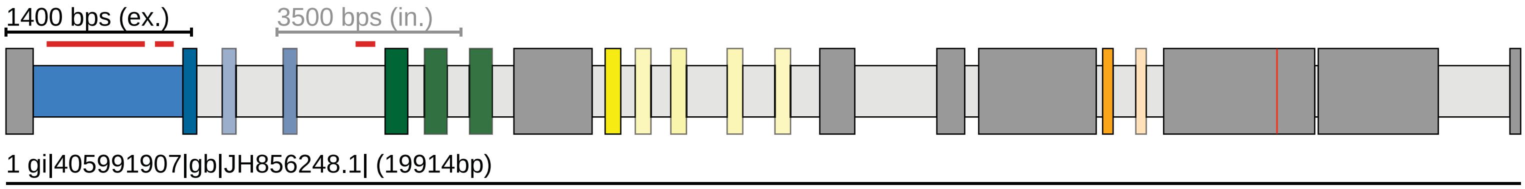

For clarity introns have been scaled down by a factor of 2.52

Drosophila hydei

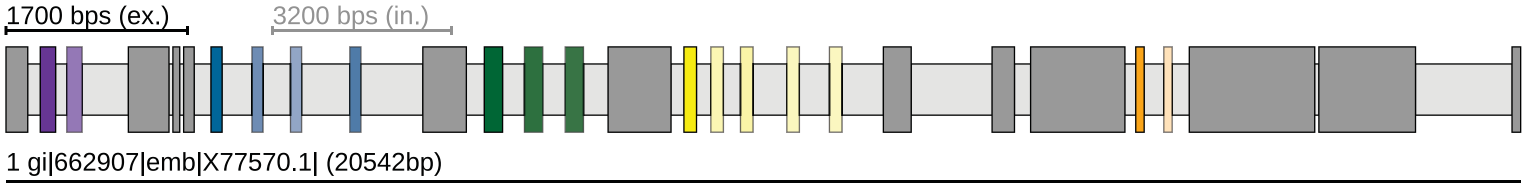

For clarity introns have been scaled down by a factor of 1.91

Drosophila mojavensis

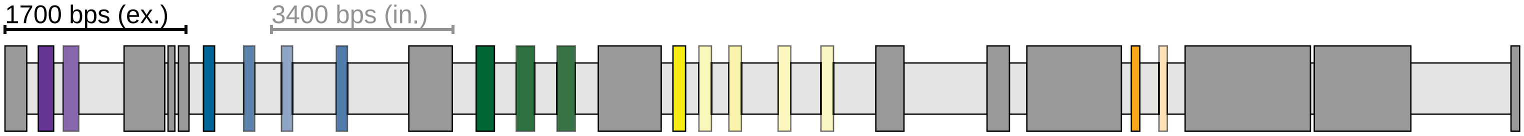

1 gi|109939711|gb|CH933807.1| (21180bp)

For clarity introns have been scaled down by a factor of 2.00

Drosophila virilis

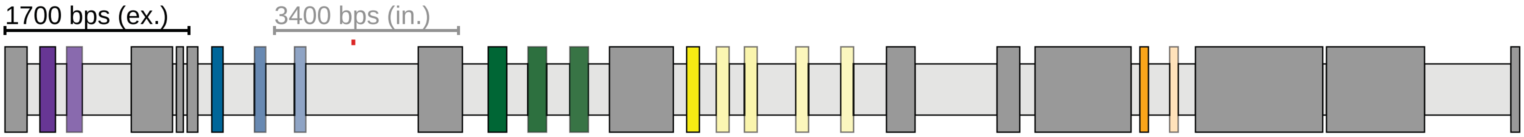

1 gi|110308853|gb|CH940649.1| (20861bp)

For clarity introns have been scaled down by a factor of 2.00

Drosophila grimshawi

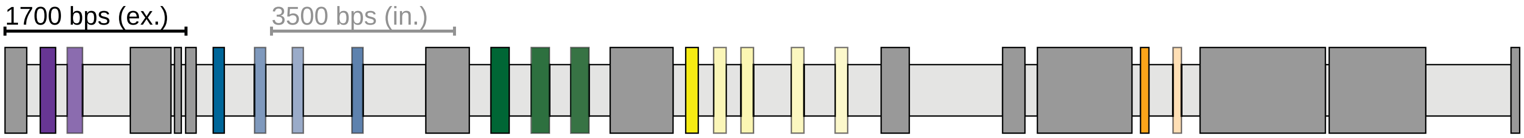

1 gi|109932861|gb|CH916368.1| (21454bp)

For clarity introns have been scaled down by a factor of 2.04

Drosophila ananassae

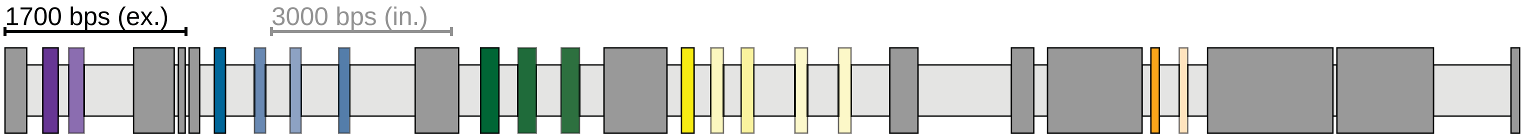

1 gi|109914392|gb|CH902620.1| (19615bp)

For clarity introns have been scaled down by a factor of 1.77

Drosophila bipectinata

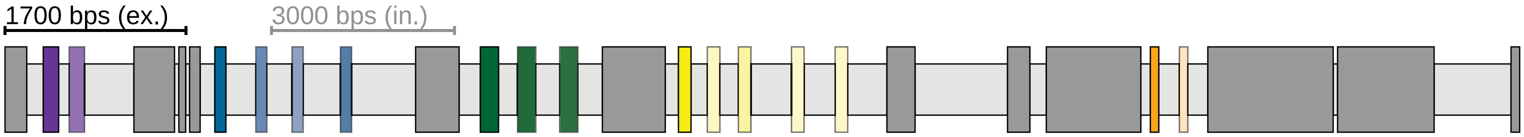

1 gi|459198958|gb|KB464239.1| (19426bp)

For clarity introns have been scaled down by a factor of 1.75

Drosophila elegans

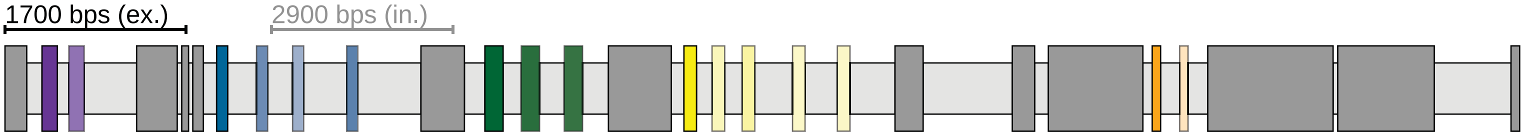

1 gi|459200709|gb|KB458274.1| (19123bp)

For clarity introns have been scaled down by a factor of 1.70

Drosophila eugracilis

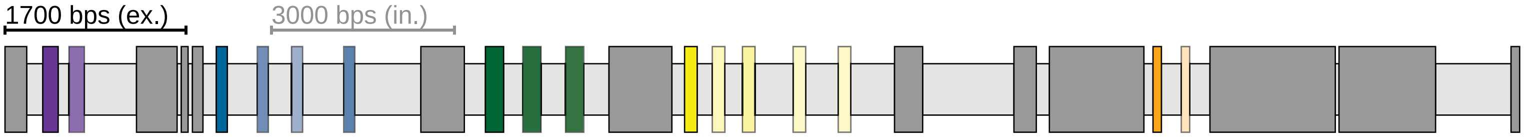

1 gi|459206219|gb|KB464922.1| (19415bp)

For clarity introns have been scaled down by a factor of 1.75

Drosophila ficusphila

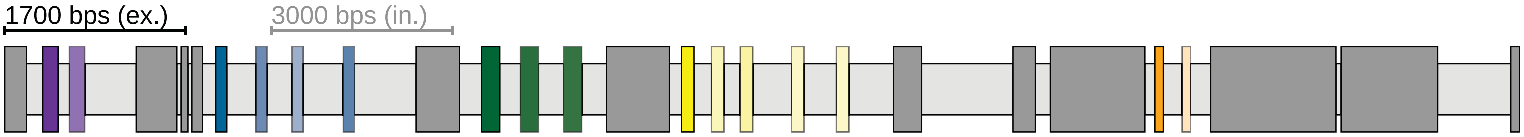

1 gi|459201459|gb|KB457528.1| (19538bp)

For clarity introns have been scaled down by a factor of 1.76

Drosophila erecta

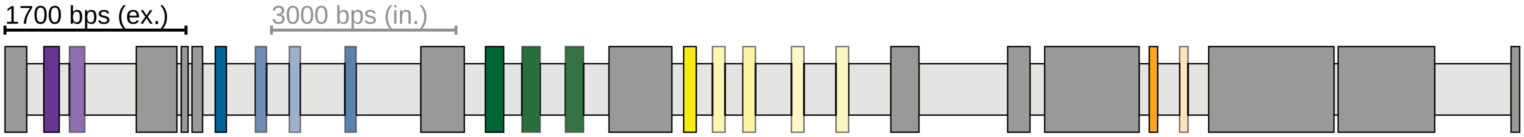

1 gi|110313977|gb|CH954179.1| (19339bp)

For clarity introns have been scaled down by a factor of 1.73

Drosophila melanogaster

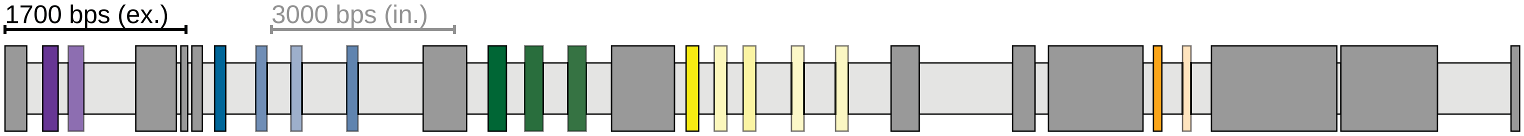

1 gi|116010444|ref|NT_033779.4| (19421bp)

For clarity introns have been scaled down by a factor of 1.75

Drosophila sechellia

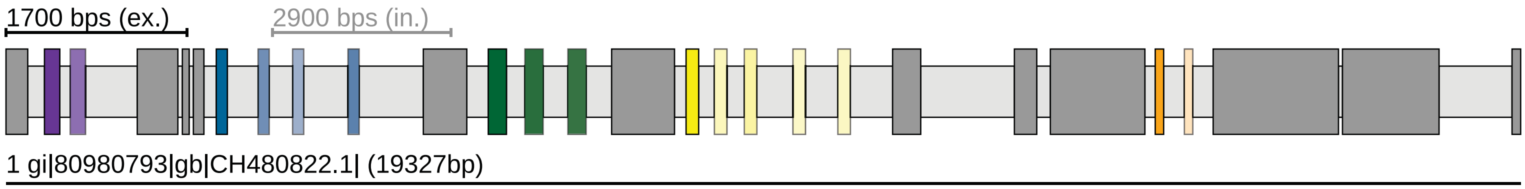

For clarity introns have been scaled down by a factor of 1.73

Drosophila yakuba

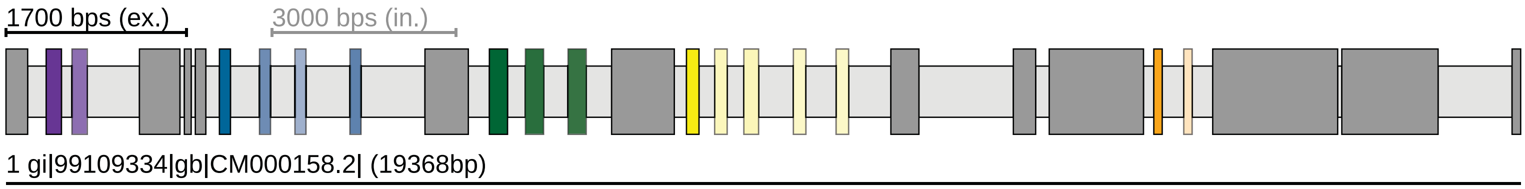

For clarity introns have been scaled down by a factor of 1.73

Drosophila kikkawai

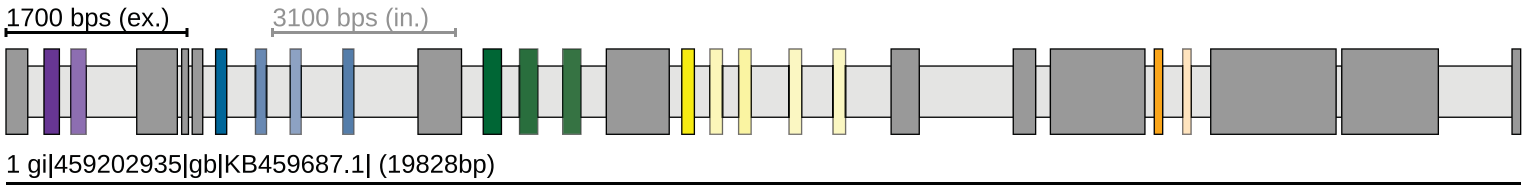

For clarity introns have been scaled down by a factor of 1.81

Drosophila rhopaloa

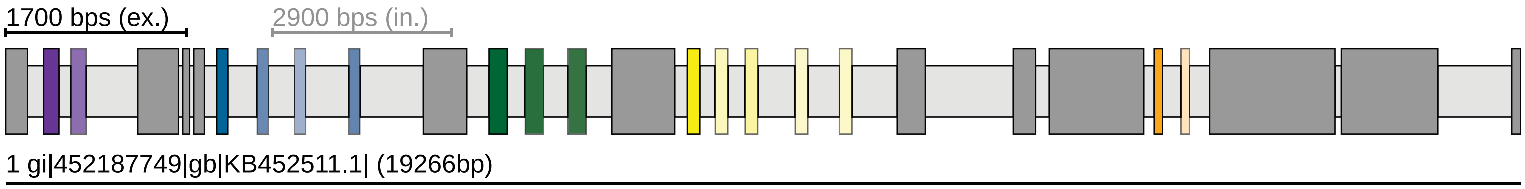

For clarity introns have been scaled down by a factor of 1.72

Drosophila biarmipes

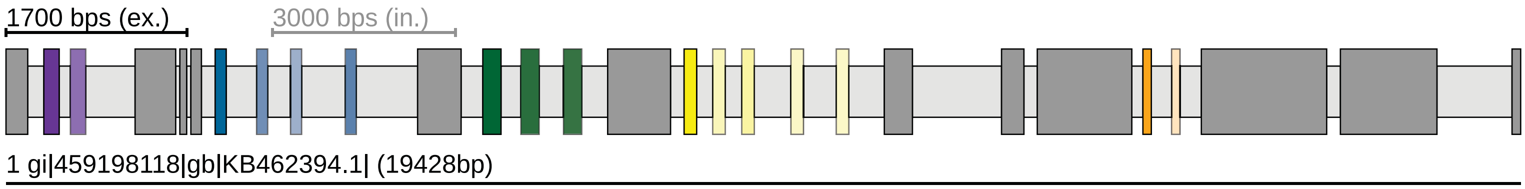

For clarity introns have been scaled down by a factor of 1.75

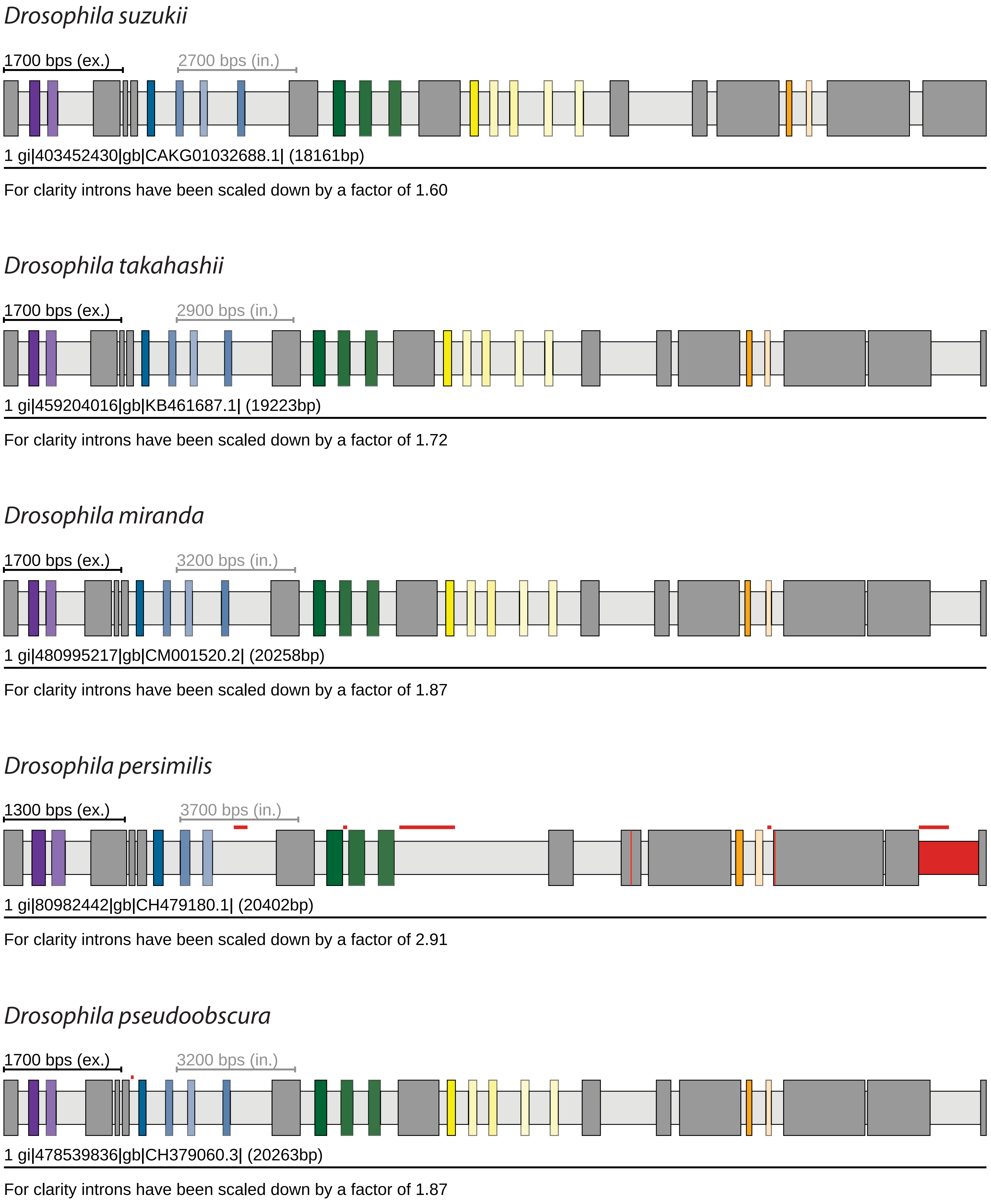
Drosophila suzukii
1700 bps (ex.)
2700 bps (in.)
1 gi|403452430|gb|CAKG01032688.1| (18161bp)
For clarity introns have been scaled down by a factor of 1.60
Drosophila takahashii
1700 bps (ex.)
2900 bps (in.)
1 gi|459204016|gb|KB461687.1| (19223bp)
For clarity introns have been scaled down by a factor of 1.72
Drosophila miranda
1700 bps (ex.)
3200 bps (in.)
1 gi|480995217|gb|CM001520.2| (20258bp)
For clarity introns have been scaled down by a factor of 1.87
Drosophila persimilis
1300 bps (ex.)
3700 bps (in.)
1 gi|80982442|gb|CH479180.1| (20402bp)
For clarity introns have been scaled down by a factor of 2.91
Drosophila pseudoobscura
1700 bps (ex.)
3200 bps (in.)
1 gi|478539836|gb|CH379060.3| (20263bp)
For clarity introns have been scaled down by a factor of 1.87

Drosophila willistoni

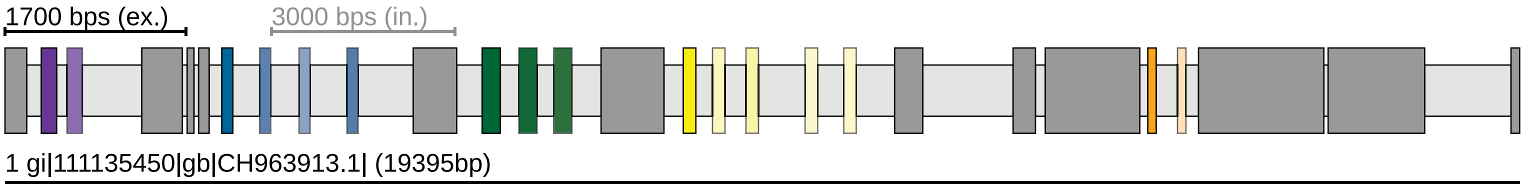

For clarity introns have been scaled down by a factor of 1.74
