# Supplementary material for: Shared Gene Structures and Clusters of Mutually Exclusive Spliced Exons within the Metazoan Muscle Myosin Heavy Chain Genes
Source: PLoS One. 2014 Feb 3;9(2):e88111. doi: 10.1371/journal.pone.0088111 (PMC3912159; doi:10.1371/journal.pone.0088111)
Supplement: Figure S3 — Detailed gene structure schemes of the lophotrochozoan Mhc genes. This file displays the gene structures including clusters of predicted MXEs and the alternative N-terminal exons leading to the Mhc genes for Crassostrea, Aplysia, Biomphalaria, and Lottia. Exons and introns are scaled, such that both exons and introns represent half of the total width of the scheme. Alternative gene start sites (methionines) and stop codons are indicated. (PDF) [file pone.0088111.s003.pdf]

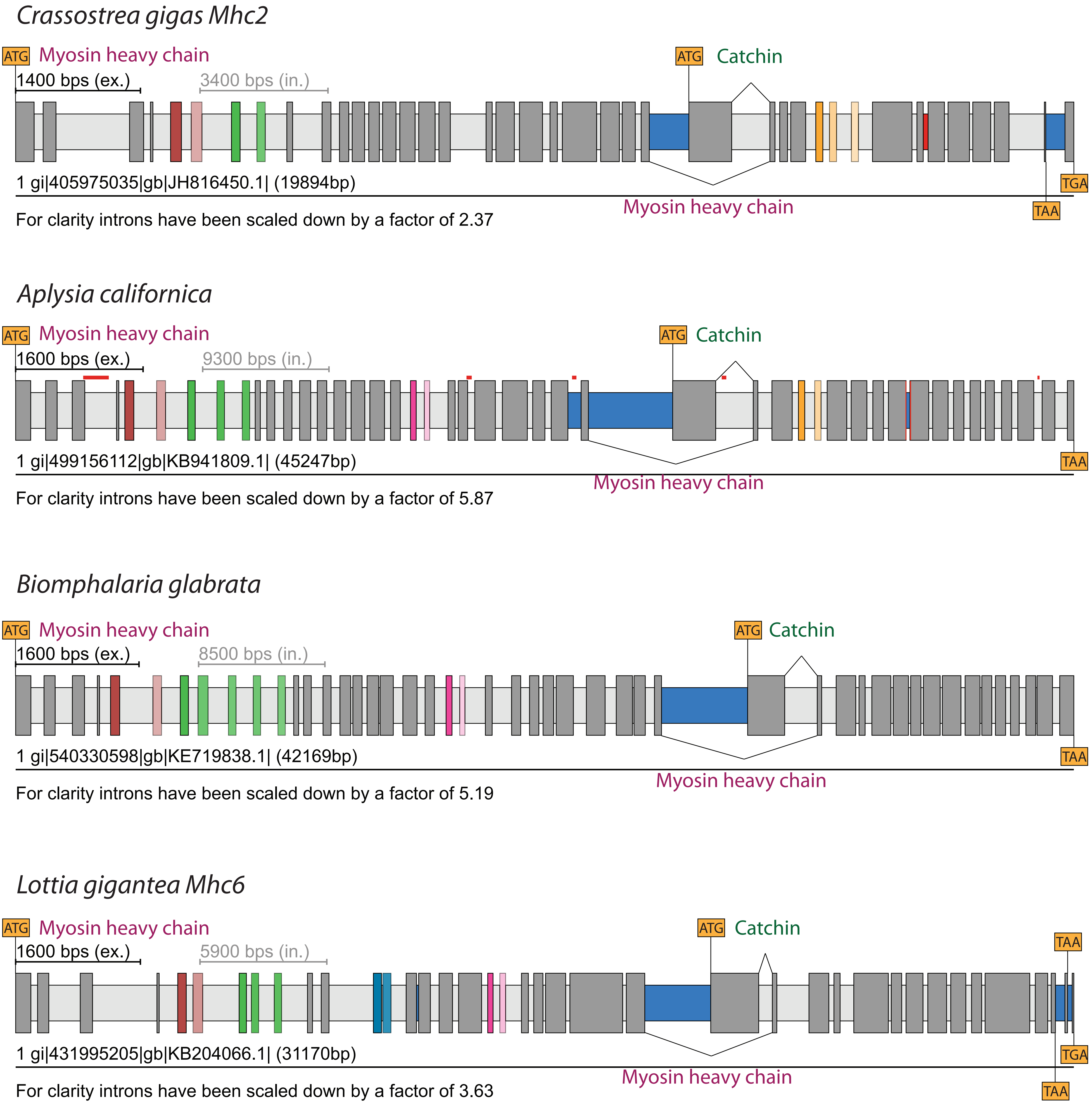
Crassostrea gigas Mhc2
ATG
Myosin heavy chain
1400 bps (ex.)
3400 bps (in.)
ATG
Catchin
1 gi|405975035|gb|JH816450.1| (19894bp)
TGA
Myosin heavy chain
TAA
For clarity introns have been scaled down by a factor of 2.37
Aplysia californica
ATG
Myosin heavy chain
1600 bps (ex.)
9300 bps (in.)
ATG
Catchin
1 gi|499156112|gb|KB941809.1| (45247bp)
TAA
Myosin heavy chain
For clarity introns have been scaled down by a factor of 5.87
Biomphalaria glabrata
ATG
Myosin heavy chain
1600 bps (ex.)
8500 bps (in.)
ATG
Catchin
1 gi|540330598|gb|KE719838.1| (42169bp)
TAA
Myosin heavy chain
For clarity introns have been scaled down by a factor of 5.19
Lottia gigantea Mhc6
ATG
Myosin heavy chain
1600 bps (ex.)
5900 bps (in.)
ATG
Catchin
TAA
1 gi|431995205|gb|KB204066.1| (31170bp)
TGA
Myosin heavy chain
TAA
For clarity introns have been scaled down by a factor of 3.63

```
                     10        20        30        40        50        60
             ....|....|....|....|....|....|....|....|....|....|....|....|
LgMhc6b      MATNGETTAPDSSDVKEESNDNTSSPQVVKSAVESVQPQEEIRDIPPPVQATPAPMESSE
CagMhc2b     -------------------------------------------MADQQPLKMTATVDVHE
AiCatchin    ---------------------------------------------------------MSD
ApcMhc1b     -------------------------------MASTEEEVPIAEAPAPSSSETVEVVQEQI
BigMhc1b     -------------------------------MASTEDEVPLVEVSAPRS---DEVIEEAI
MygMhc1b     -------------------------------MSDTKITVTSTISTEEKP-----VVVKEE
LgMhc8       ------------------------------------------------------------

                     70        80        90       100       110       120
             ....|....|....|....|....|....|....|....|....|....|....|....|
LgMhc6b      ADTTKDQIVIKRSVESQPPAAPVSVPPKVTPAIKRPEEPEPETRPRTKKPSSTTSSSASR
CagMhc2b     TSKAEEVSTPAPTAATVTAVRSTVKANATKNEPQKIKEAAAQPTPTKKSSITTSSSSTTS
AiCatchin    GEQLKVEFSSSVVETSKDESPQPPPLDTPSPQAASVIEVKSQVKATSNPAPKSEIAPAET
ApcMhc1b     TV-KRSEPAPEPPKPVVRVEEDFVETRPRNPPAPAPAPAKSSSSSSNSKKQSSSSSSKVK
BigMhc1b     IVTKKTEP-KKPSASPVPRDENLEETRPRQ---------------SSSKQASSSSSSKHK
MygMhc1b     SATVER---IPTP---------DIISE----PPAPVAAKVTTVTEKSTSKASSSQLSPKM
LgMhc8       ------------------------------------------------------------

                    130       140       150       160       170       180
             ....|....|....|....|....|....|....|....|....|....|....|....|
LgMhc6b      VVSSSSSSRART-------SSDYQYGDYLGALSREFRGTSPAVLENIATHPLLYSKAYEP
CagMhc2b     SGKKTVSTSKKT--------VQYQSDDILGDLTRNYRGTSPAVLEGIAHHPALFSRAYEP
AiCatchin    RKTTTTTTTSSKKSTKSSTSRSSGYSDYLGDISRDYRGVSPAVIESIATHPVLFSRSYQY
ApcMhc1b     SSSSSSSSSRTKSYHTYSQPREYGDRLGIASLARDFRGTSPSVIENIATHPLLYSPGYEP
BigMhc1b     SSSSHKSSTKSYHS--SSQPRDYSDRLGIGGFSREFRGMSPSVVENIATHPLLFNKGYEP
MygMhc1b     SSSQTTTRTTKTS---VGRSSDYES--TIGQLTRDYRGTSPAVLEGIASHPILYSKTFDS
LgMhc8       ------------------------------------------------------------

                    190       200       210       220       230       240
             ....|....|....|....|....|....|....|....|....|....|....|....|
LgMhc6b      TYGS-KLSARSKKVIRDTAELATSCPGLKALLEARIEELEEELEAERQARAKVEKQRNEL
CagMhc2b     VNP--RLSAKSKKIIRETSDLAIFSPGLKNLLEARIEELEEELEAERAARTKVEKQRAEV
AiCatchin    EPRSTRISARSKRVLREASDLSLTSPGLKGLLEARIEELEEELEAERNARAKVEKQRAEL
ApcMhc1b     LRNP-HLSARSKKILRDTSDLGVVAPGLKAMLEARIEELEEELEAERQSRAKVEKQRTEL
BigMhc1b     LINP-ILSAKSKKVIRDTSDLGVYAPGLKALLEARIEELEEELEAERQARAKVEKQRTEL
MygMhc1b     KIGRQKLSARSKKILRDTSDLAIVAPGLKNLLEARIEELEEELEAERAARTKVDKQRAEI
LgMhc8       --------------------------MTARALEARVEELELDLETERQAKVKSEKQRVDL
```

Catchin

Catchin /Mhc
